# Green Synthesis of Tetrahydropyrazino[2,1-a:5,4-a′]diisoquinolines as SARS-CoV-2 Entry Inhibitors

**DOI:** 10.1021/acsomega.4c08640

**Published:** 2024-12-20

**Authors:** Sowndarya Palla, Srinivasa Rao Palla, Jia-Jin Liu, Tai-Ling Chao, Ting-Hui Lee, Veerababurao Kavala, I-Chen Liu, Lily Hui-Ching Wang, Sui-Yuan Chang, Ching-Fa Yao, Po-Huang Liang

**Affiliations:** † Institute of Biological Chemistry, Academia Sinica, Taipei 11529, Taiwan; ‡ Institute of Biochemical Sciences, National Taiwan University, Taipei 10617, Taiwan; § Taiwan International Graduate Program, Academia Sinica, Taipei 11529, Taiwan; ∥ Department of Chemistry, National Taiwan Normal University, Taipei 11677, Taiwan; ⊥ Department of Clinical Laboratory Sciences and Medical Biotechnology, National Taiwan University, Taipei 10048, Taiwan; # Department of Life Science, National Tsing Hua University, Hsinchu 30013, Taiwan; ∇ Institute of Molecular and Cellular Biology, National Tsing Hua University, Hsinchu 30013, Taiwan; ○ Department of Laboratory Medicine, National Taiwan University Hospital, Taipei 10002, Taiwan

## Abstract

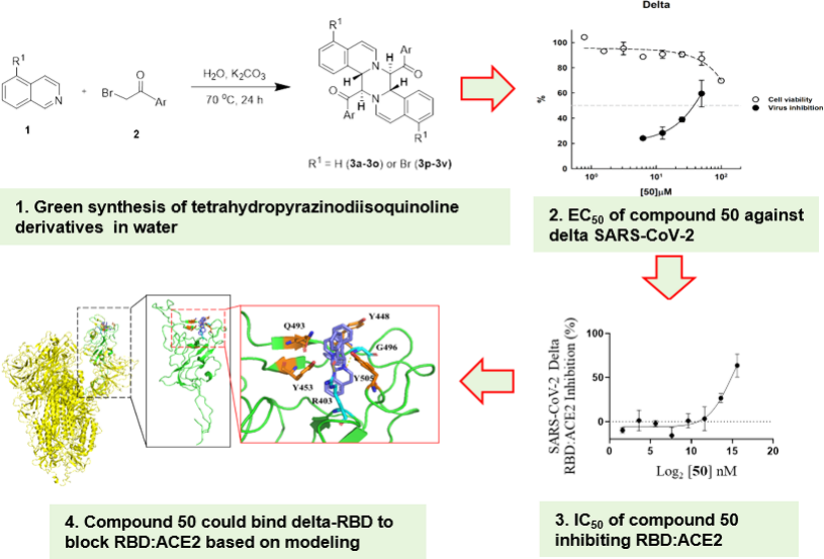

A class of tetrahydropyrazino[2,1-a:5,4-a′]diisoquinoline derivatives were synthesized under environmentally friendly conditions using water as the solvent. The 3-D structures of some synthesized compounds were determined by X-ray diffraction. Since naturally occurring isoquinoline alkaloids have significant antiviral activities against a wide range of viruses, including coronaviruses, the synthesized compounds were assayed for their inhibitory activities against SARS-CoV-2. Our results showed that the active compounds **50** and **96** blocked the delta SARS-CoV-2 entry into VeroE6 cells to display EC_50_ of 26.5 ± 6.9 and 17.0 ± 3.7 μM, respectively, by inhibiting the interaction between SARS-CoV-2 Spike’s receptor binding domain (RBD) and human receptor angiotensin-converting enzyme 2 (ACE2), and CC_50_ greater than 100 μM. This study provides a green synthesis method of tetrahydropyrazinodiisoquinoline for antiviral or other applications.

## 1 Introduction

Since December 2019, coronavirus disease 2019 (COVID-19) has caused a worldwide pandemic with an approximately 1% case mortality rate.^1−3^ The pathogen is human severe acute respiratory syndrome-coronavirus-2 (SARS-CoV-2) with a genome highly similar (79.6% identity) to that of the previously (2002–2003) emerged SARS-CoV.^4,5^ Unlike another CoV called Middle East Respiratory Syndrome-CoV (MERS-CoV) that employs dipeptidyl peptidase-4 (DPP4) as the human receptor to anchor the virus, SARS-CoV and SARS-CoV-2 utilize human angiotensin-converting enzyme 2 (ACE2) to bind with the virus surface Spike glycoprotein through the receptor binding domain (RBD).^6^ The virus entry after RBD:ACE2 interaction is initiated by the cleavage of Spike at S1/S2 and S2′ sites by two human proteases, Furin and transmembrane protease serine 2 (TMPRSS2), respectively. The primed Spike exposes the membrane fusion peptide to fuse with the host cell membrane and the virus’s RNA enters into the cell.^7^ TMPRSS2 could replace the function of Furin so that the irreversible serine protease inhibitors Camostat and Nafamost antagonize SARS-CoV-2 by inhibiting TMPRSS2 as off-target.^8,9^ Alternatively to the above pathway, another human protease Cathepsin L is involved in the endosomal virus entry, mainly found in SARS-CoV-2 omicron strain.^10^

After entry into host cell, the 29.7-kb SARS-CoV-2 positive-sense single-strand RNA is translated by human cell machinery to form two polyproteins pp1a and pp1ab that can be processed by two virus-encoded proteases, 3C-like protease (3CL^pro^) as homologous to the picornavirus 3C protease and papain-like protease (PL^pro^), into 16 functional nonstructural proteins (NSPs).^11−13^ Mature NSPs, including NSP7, 8, 10, 12, and 13 then form a replication-transcription complex with RNA-dependent RNA polymerase (RdRp), helicase, exonuclease, and other activities for making subgenomic RNAs of the structural proteins, Envelop (E), Nucleocapsid (N), Membrane (M), and Spike (S) proteins. The structural proteins are assembled with the RNA into new virus particles.^14^ Therefore, these two viral proteases and RdRp are attractive drug targets, and many covalent or noncovalent inhibitors have been discovered. To date, Pfizer Co. has developed Paxlovid, a nitrile-based covalent 3CL^pro^ peptidomimetic inhibitor nirmatrelvir combined with ritonavir for inhibiting CYP3A4 as a market drug.^15^ A noncovalent nonpeptidomimetic 3CL^pro^ inhibitor ensitrelvir has been approved for use in Japan.^16^ In China, leritrelvir^17^ and simnotrelvir^18,19^ have been conditionally approved. Remdesivir, a nucleotide analog prodrug developed by Gilead Co., was found to inhibit SARS-CoV-2 RdRp.^20,21^ It showed promising efficacy in clinical trials and thus was approved for COVID-19 treatment.^22,23^ Another nucleotide analog, molnupiravir, a prodrug of the nucleoside analog N4-hydroxycytidine developed by Merck Co. has also been approved for anti-COVID-19.^24,25^ For preventing virus entry, several antibodies have been developed as approved drugs.^26^ However, no small-molecule drug to inhibit virus entry has been approved, although some small-molecule inhibitors of RBD:ACE2 interaction and/or human proteases have been identified.^27−32^ However, resistance problems have occurred on various anti-COVID-19 drugs.^33−37^ Therefore, continuing to search for drug candidates is still necessary.

**Figure 1:**
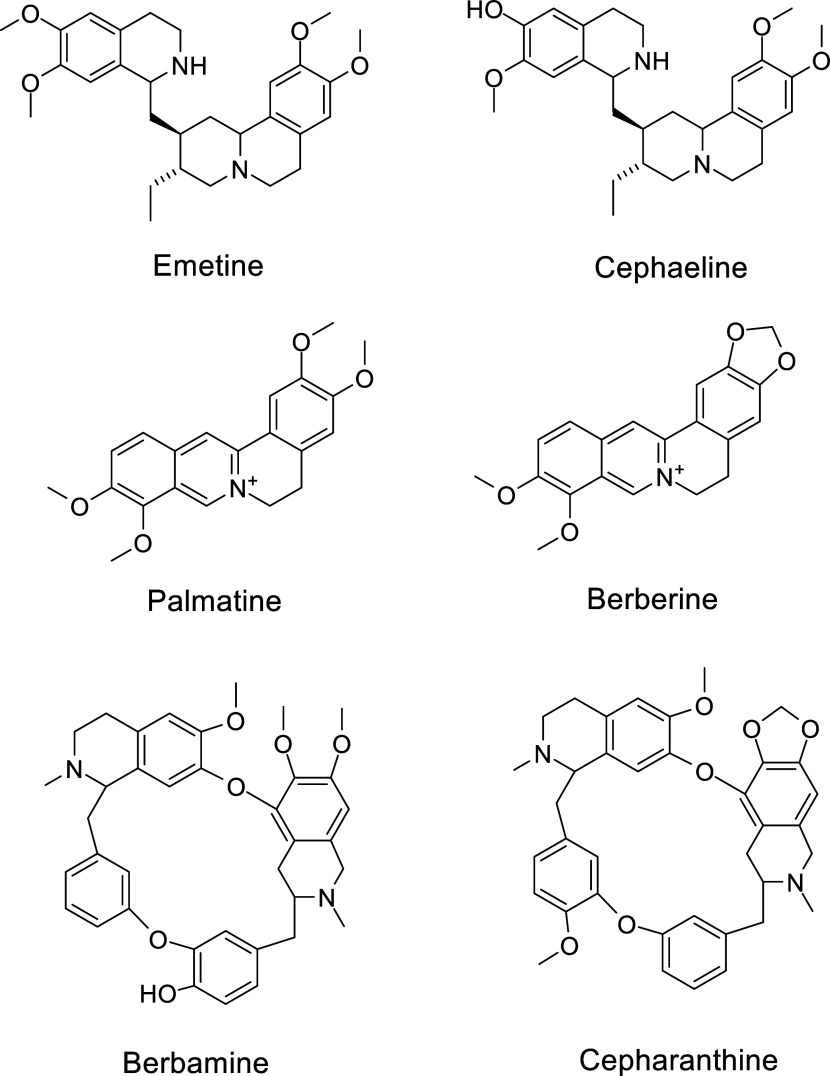
Chemical structures of some biologically active isoquinoline derivatives. These include emetine and its OCH_3_ group reduced analogue cephaline that shows lower toxicity, palmatine, and berberine containing a nitrogen cation, as well as berbamine, and cepharanthine in cyclic forms.

Isoquinoline alkaloids are a class of naturally occurring molecules that have significant antiviral activities against a wide range of viruses, including CoVs.^38^ Isoquinoline is a unique nucleus in chemistry, which structurally is composed of two rings of benzene and pyridine in such a way that the nitrogen of pyridine is placed away from the benzene ring. Isoquinoline alkaloids usually include all alkaloids that have various isoquinoline analogs, for example, 1,2,3,4-tetrahydroisoquinoline (containing a saturated pyridine ring with a secondary amine) such as tylophorine, dicentrine, corydaline, discretine, lycorine, emetine, argemonine, etc. (the chemical structures of emetine and cephaline with the OCH_3_ being reduced to OH to decrease cytotoxicity, are shown in Figure 1 as examples). In addition, an important class of isoquinoline alkaloids have interconnected rings, in which the nitrogen atom has become a quaternary cation (the chemical structures of palmatine and berberine are shown in Figure 1 as examples). The isoquinoline alkaloids such as emetine, palmatine, and berberine could inhibit SARS-CoV-2 by multiple-targeting S protein, RdRp, eIF4A, N, and/or orf6 proteins.^39^ Moreover, a cyclic bis-benzylisoquinoline alkaloid berbamine (structure shown in Figure 1) could inhibit SARS-CoV-2 by binding to the postfusion core of Spike’s S2 subunit.^40^ From a library containing 188 natural products, cyclic bis-benzylisoquinoline alkaloids such as cepharanthine (structure shown in Figure 1) were identified as SARS-CoV-2 entry inhibitors.^41^

Similar to cyclic bis-benzylisoquinoline alkaloids, our *in silico* prestudy revealed that the extended structures of diisoquinoline derivatives may block RBD:ACE2 interaction, thereby inhibiting SARS-CoV-2 entry. As presented herein, we developed a green synthesis protocol using water as solvent for tetrahydropyrazino[2,1-a:5,4-a′]diisoquinoline derivatives from isoquinoline or bromoisoquinoline and various phenacyl bromides in water and optimized their yields. Since regulations are becoming increasingly severe regarding production and use of organic solvents, forcing chemists to find greener and safer alternatives, water as the main if not exclusive reaction medium for organic transformations represents a safe, nontoxic, cheap, and environmentally friendly alternative (42 for a review of the reactions using water as solvent). The synthesized compounds were characterized for their three-dimensional (3D) structures by X-ray diffraction and evaluated for antagonizing SARS-CoV-2 through inhibiting RBD:ACE2 interaction. The green methodology developed here is useful to synthesize tetrahydropyrazinodiisoquinoline for antiviral or other applications.

## 2 Experimental Methods

### 2.1 General Methods

Reactions for synthesizing compounds were monitored by using thin-layer chromatography (TLC) on silica gel. Flash chromatography was performed on silica gel of 60–200 μm particle size for compound purification. Yields were reported for spectroscopically pure compounds. Melting points were recorded on the Fargo MP-1D Melting Point Apparatus. NMR spectra were recorded on a Bruker AVIIIHD 400 MHz FT-NMR instrument in the Department of Chemistry, National Taiwan Normal University. Chemical shifts were given in δ values relative to tetramethylsilane (TMS); coupling constants *J* were given in Hz. Internal standards were CDCl_3_ (δ_H_ = 7.24) or DMSO-*d*_6_ (δ_H_ = 2.49) for ^1^H NMR spectra, and CDCl_3_ (δ_C_ = 77.0) or DMSO-*d*_6_ (δ_C_ = 39.5) for ^13^C NMR spectra. The splitting patterns were reported as s (singlet), d (doublet), t (triplet), q (quartet), m (multiplet), br (broad), and dd (double of doublets). High-resolution mass spectra were measured by a Bruker UPLC-MS instrument in the TechComm core facility, Department of Chemistry, National Taiwan Normal University.

### 2.2 Synthesis of Tetrahydropyrazino[2,1-a:5,4-a′]diisoquinoline Derivatives

A mixture of isoquinoline **1** (1 mmol), 2-bromoacetophenone **2** (1 mmol), K_2_CO_3_ (1 mmol), and H_2_O (0.5 mL) was stirred at 70 °C for 24 h. Upon the consumption of isoquinoline **1** (monitored by TLC), the reaction was cooled to room temperature, filtered, concentrated, and purified by silica gel flash chromatography (Hex/EtOAc) to afford compound **3a**. Other compounds were synthesized from isoquinoline or bromoisoquinoline and various phenacyl bromides under the same condition.

#### 2.2.1 Compound **3a** (**50**)

Light yellow solid, 86% yield. Mp: 178–179 °C. ^1^H NMR (400 MHz, CDCl_3_): δ 7.75 (dd, *J* = 6.84 Hz, 4H), 7.47–7.42 (m, 2H), 7.35–7.31 (m, 4H), 7.06–7.01 (m, 2H), 6.90–6.83 (m, 6H), 6.37 (dd, *J* = 7.56 Hz, 2H), 5.59 (d, *J* = 7.28 Hz, 2H), 5.43 (d, *J* = 7.44 Hz, 2H), 5.01 (d, *J* = 8.9 Hz, 2H), ^13^C NMR (100 MHz, CDCl_3_): δ 194.6, 138.1, 136.2, 133.2, 131.9, 131.6, 128.6, 128.4, 128.3, 127.3, 126.2, 125.6, 123.2, 101.2, 65.1, 50.2. HRMS (ESI): *m*/*z* calculated for C_34_H_27_N_2_O_2_^+^ [M + H]^+^: 495.2067, found 495.2051.

#### 2.2.2 Compound **3b** (**105**)

Yellow solid, 53% yield. Mp: 136–137 °C. ^1^H NMR (400 MHz, CDCl_3_): δ 7.29 (d, *J* = 6.84 Hz, 2H), 7.25 (s, 5H), 7.20–7.16 (m, 2H), 7.13 (dd, *J* = 6.24 Hz, 1H), 7.09 (dd, *J* = 4.72 Hz, 2H), 6.99 (td, *J* = 7.44 Hz, 2H), 6.89 (d, *J* = 7.56 Hz, 2H), 6.01 (d, *J* = 7.28 Hz, 2H), 5.42 (d, *J* = 7.44 Hz, 2H), 5.26 (d, *J* = 8.88 Hz, 2H), 4.81 (d, *J* = 8.84 Hz, 2H). ^13^C NMR (100 MHz, CDCl_3_): δ 198.1, 152.5, 138.2, 137.8, 131.8, 131.6, 130.9, 130.2, 129.3, 128.1, 126.7, 125.7, 123.5, 120.5, 100.9, 68.7, 49.9. HRMS (ESI): *m*/*z* calculated for C_34_H_24_Cl_2_^(35)^N_2_O_2_^+^ [M]^+^: 564.1180, found 564.1101 and for C_34_H_24_Cl_2_^(37)^N_2_O_2_^+^ [M]^+^: 566.1150, found 566.1160.

#### 2.2.3 Compound **3c** (**110**)

Light yellow solid, 61% yield. Mp: 172–173 °C. ^1^H NMR (400 MHz, CDCl_3_): δ 7.62 (dd, *J* = 5.96 Hz, 3H), 7.37–7.33 (m, 2H), 7.08–7.04 (m, 2H), 6.96–6.84 (m, 8H), 6.75 (d, *J* = 8.36 Hz, 2H), 6.19 (d, *J* = 7.48 Hz, 2H), 5.44 (d, *J* = 7.44 Hz, 2H), 5.30 (d, *J* = 9.24 Hz, 2H), 5.16 (d, *J* = 8.92 Hz, 2H), 3.44 (s, 6H). ^13^C NMR (100 MHz, DMSO-*d*_6_) δ: 199.0, 158.0, 139.0, 133.5, 132.4, 130.7, 127.5, 127.0, 123.0, 122.9, 120.8, 111.3, 99.2, 68.9, 55.2, 51.1. HRMS (ESI): *m*/*z* calculated for C_36_H_31_N_2_O_4_ [M + H]^+^: 555.2278, found 555.2269.

#### 2.2.4 Compound **3d** (**103**)

Light orange solid, 53% yield. Mp: 177–178 °C. ^1^H NMR (400 MHz, CDCl_3_): δ 7.23 (d, *J* = 1.28 Hz, 1H), 7.16–7.06 (m, 9H), 6.96–6.89 (m, 6H), 6.12 (t, *J* = 7.52 Hz, 2H), 5.45 (d, *J* = 7.56 Hz, 2H), 5.43 (d, *J* = 7.56 Hz, 2H), 4.79 (d, *J* = 9.08 Hz, 2H), 2.38 (s, 6H). ^13^C NMR (100 MHz, CDCl_3_): δ 198.4, 138.5, 138.1, 137.6, 132.0, 131.7, 131.0, 128.1, 127.7, 127.5, 126.6, 125.5, 125.3,123.5, 100.6, 67.4, 49.9, 20.7. HRMS (MALDI-TOF): *m*/*z* calculated for C_36_H_30_N_2_O_2_^+^ [M]^+^: 522.2307, found 522.2301.

#### 2.2.5 Compound **3e** (**74**)

Whitish yellow solid, 60% yield. Mp: 140–141 °C. ^1^H NMR (400 MHz, CDCl_3_): δ = 7.79 (dd, *J* = 7.76 Hz, 4H), 7.10–6.86 (m, 12H), 6.37 (d, *J* = 7.44 Hz, 2H), 5.62 (d, *J* = 7.4 Hz, 2H), 5.40 (d, *J* = 8.8 Hz, 2H), 4.96 (d, *J* = 8.84 Hz, 2H). ^13^C NMR (100 MHz, DMSO-*d*_6_): δ = 194.4, 166.9, 164.4, 138.6, 132.5, 132.4, 132.3, 128.4, 127.5, 126.3, 125.9, 123.8, 116.3, 116.1, 101.0, 64.3, 50.2. HRMS (ESI): *m*/*z* calculated for C_34_H_25_F_2_N_2_O_2_^+^ [M + H]^+^: 531.1879, found 531.1885.

#### 2.2.6 Compound **3f** (**96**)

Yellow solid, 54% yield. Mp: 160–161 °C. ^1^H NMR (400 MHz, CDCl_3_): δ 7.68–7.66 (m, 4H), 7.32–7.28 (m, 4H), 7.09–7.05 (m, 2H), 6.93 (d, *J* = 7.48 Hz, 2H), 6.89–6.84 (m, 4H), 6.34 (dd, *J* = 7.48 Hz, 2H), 5.62 (d, *J* = 7.48 Hz, 2H), 5.36 (dd, *J* = 8.8 Hz, 2H), 4.92 (d, *J* = 8.8 Hz, 2H). ^13^C NMR (100 MHz, CDCl_3_): δ 193.7, 139.7, 137.7, 134.4, 131.7, 130.9, 130.0, 128.8, 128.1, 127.6, 127.2, 126.7, 125.8, 123.7, 101.5, 65.2, 50.2. HRMS (ESI): *m*/*z* calculated for C_17_H_13_ClNO^2+^ [M/2 + H]^2+^: 282.0680, found 282.0686.

#### 2.2.7 Compound **3g** (**68**)

Light yellow solid, 30% yield. Mp: 173–174 °C. ^1^H NMR (400 MHz, CDCl_3_): δ = 7.66 (d, *J* = 7.80 Hz, 4H), 7.14 (d, *J* = 7.84 Hz, 4H), 7.05 (t, *J* = 6.68 Hz, 2H), 6.91–6.81 (m, 6H), 6.38 (d, *J* = 7.36 Hz, 2H), 5.58 (d, *J* = 7.36 Hz, 2H), 5.44 (d, *J* = 8.84 Hz, 2H), 4.99 (d, *J* = 8.84 Hz, 2H), 2.32 (s, 6H). ^13^C NMR (100 MHz, CDCl_3_): δ = 194.3, 144.0 138.1, 133.7, 131.9, 129.1, 128.8, 128.2, 127.9, 127.8, 127.6, 126.8, 125.5, 123.4, 100.8, 65.0, 50.2, 21.5. HRMS (ESI): *m*/*z* calculated for C_36_H_31_N_2_O_2_^+^ [M + H]^+^: 523.2380, found 523.2383.

#### 2.2.8 Compound **3h** (**72**)

Light orange solid, 21% yield. Mp: 152–153 °C. ^1^H NMR (400 MHz, CDCl_3_): δ 7.74 (d, *J* = 8.84 Hz, 4H), 7.06–7.02 (m, 2H) 6.91 (d, *J* = 7.4 Hz, 2H), 6.86–6.79 (m, 8H), 6.38 (d, *J* = 7.4 Hz, 2H), 5.57 (d, *J* = 7.44 Hz, 2H), 5.47 (d, *J* = 8.9 Hz, 2H), 4.98 (d, *J* = 8.9 Hz, 2H), 3.79 (s, 6H). ^13^C (100 MHz, CDCl_3_): δ 193.4, 163.5, 138.1, 132.0, 131.0, 129.2, 127.9, 127.8, 126.8, 125.5, 123.3, 113.7, 100.6, 64.9, 60.3, 55.4, 50.4, 14.1. HRMS (ESI): *m*/*z* calculated for C_36_H_31_N_2_O_4_^+^ [M + H]^+^: 555.2278, found 555.2279.

#### 2.2.9 Compound **3i** (**115**)

Light yellow solid, 57% yield. Mp: 163–164 °C. ^1^H NMR (400 MHz, CDCl_3_): δ 7.88 (d, *J* = 8.4 Hz, 3H), 7.60–7.47 (m, 8H), 7.47–7.39 (m, 8H), 7.11 (td, *J* = 7.16 Hz, 2H), 6.97–6.87 (m, 5H), 6.47 (d, *J* = 7.5 Hz, 2H), 5.66 (d, *J* = 7.44 Hz, 2H), 5.51 (d, *J* = 8.9 Hz, 2H), 5.08 (d, *J* = 8.9 Hz, 2H). ^13^C NMR (100 MHz, DMSO-*d*_6_) δ: 194.2, 145.9, 139.7, 138.1, 131.9, 130.4, 129.3, 129.0, 128.9, 128.2, 128.0, 127.8, 127.6, 127.2, 127.1, 126.8, 126.6, 126.4, 125.7, 123.5, 101.2, 65.2, 50.2. HRMS (ESI): *m*/*z* calculated for C_46_H_35_N_2_O_2_^+^ [M + H]^+^: 647.2693, found 647.2660.

#### 2.2.10 Compound **3j** (**106**)

Light orange solid, 60% yield. Mp: 167–168 °C. ^1^H NMR (400 MHz, CDCl_3_): δ 8.10 (s, 2H), 7.87–7.80 (m, 2H), 7.81–7.80 (m, 5H), 7.56–7.51 (m, 2H), 7.49–7.46 (m, 3H), 7.05–6.99 (m, 2H), 6.94 (d, *J* = 6.84 Hz, 2H), 6.88 (d, *J* = 7.12 Hz, 2H), 6.79 (td, *J* = 6.32 Hz, 2H), 6.48 (d, *J* = 7.52 Hz, 2H), 5.65 (d, *J* = 7.44 Hz, 2H), 5.57 (d, *J* = 8.9 Hz, 2H), 5.22 (d, *J* = 8.9 Hz, 2H). ^13^C NMR (100 MHz, CDCl_3_): 195.1, 152.4, 142.8, 138.0, 135.5, 133.6, 132.2, 132.0, 130.6, 129.6, 127.6, 127.6, 126.9, 126.6, 125.6, 124.2, 123.5, 120.5, 101.0, 65.3, 50.8. HRMS (ESI): *m*/*z* calculated for C_21_H_16_NO^2+^ [M/2 + H]^2+^: 298.1226, found 298.1232.

#### 2.2.11 Compound **3k** (**111**)

Light orange solid, 70% yield. Mp: 174–175 °C. ^1^H NMR (400 MHz, CDCl_3_): δ 8.37 (d, *J* = 8.08 Hz, 2H), 7.89 (d, *J* = 8.08 Hz, 2H), 7.82 (d, *J* = 7.4 Hz, 3H), 7.54–7.46 (m, 7H), 7.34–7.28 (m, 4H), 7.10 (td, *J* = 6.32 Hz, 2H), 7.01 (t, *J* = 6.96 Hz, 2H), 6.91–6.85 (m, 3H), 6.12 (t, *J* = 6.68 Hz, 2H), 5.71 (d, *J* = 9.4 Hz, 2H), 5.44 (d, *J* = 7.48 Hz, 2H), 5.04 (d, *J* = 9.2 Hz, 2H). ^13^C NMR (100 MHz, CDCl_3_); δ 198.8, 138.1, 135.4, 133.8, 132.7, 132.1, 130.5, 128.4, 128.2, 128.0, 127.6, 127.4, 126.8, 126.5, 125.6, 125.6, 124.2, 123.6, 100.7, 67.8, 50.7. HRMS (ESI): *m*/*z* calculated for C_42_H_31_N_2_O_2_^+^ [M + H]^+^: 595.2380, found 595.2386.

#### 2.2.12 Compound **3l** (**88**)

Yellow solid, 22% yield. Mp: 148–149 °C. ^1^H NMR (400 MHz, CDCl_3_): δ 7.41 (d, *J* = 1.92 Hz, 2H), 7.26–7.23 (m, 2H), 7.07–7.03 (m, 2H) 6.92 (d, *J* = 7.5 Hz, 2H), 6.85 (d, *J* = 4.40 Hz, 4H), 6.72 (d, *J* = 8.48 Hz, 2H), 6.37 (d, *J* = 6.92 Hz, 2H), 5.57 (d, *J* = 7.44 Hz, 2H), 5.48 (d, *J* = 8.84 Hz, 2H), 4.99 (d, *J* = 8.96 Hz, 2H), 3.86 (s, 12H). ^13^C (100 MHz, CDCl_3_): δ 193.5, 153.4, 149.0, 138.0, 131.9, 129.3, 127.9, 127.8, 126.9, 125.5, 123.4, 123.2, 111.0, 109.9, 100.6, 64.9, 56.0, 55.8, 50.7. HRMS (ESI): *m*/*z* calculated for C_38_H_35_N_2_O_6_^+^ [M + H]^+^: 615.2490, found 615.2497.

#### 2.2.13 Compound **3m** (**98**)

Yellow solid, 78% yield. Mp: 176–177 °C. ^1^H NMR (400 MHz, CDCl_3_): δ = 7.27–7.24 (m, 3H), 7.07–7.02 (m, 2H), 6.92 (d, *J* = 7.48 Hz, 2H), 6.86–6.82 (m, 5H), 6.69 (d, *J* = 8.0 Hz, 2H), 6.34 (d, *J* = 7.52 Hz, 2H), 5.97–5.96 (m, 4H), 5.57 (d, *J* = 7.48 Hz, 2H), 5.43 (d, *J* = 8.92 Hz, 2H), 4.92 (d, *J* = 8.96 Hz, 2H). ^13^C NMR (100 MHz, CDCl_3_): δ = 192.9, 151.9, 148.1, 137.9, 131.9, 131.0, 127.9, 127.7, 126.8, 125.5, 125.0, 123.4, 108.4, 107.7, 101.8, 100.8, 64.9, 50.5. HRMS (ESI): *m*/*z* calculated for C_36_H_27_N_2_O_6_^+^ [M + H]^+^: 583.1864, found 583.1867.

#### 2.2.14 Compound **3n** (**114**)

Light orange solid, 30% yield. Mp: 153–154 °C. ^1^H NMR (400 MHz, CDCl_3_): δ 7.64–7.61 (m, 4H), 7.51–7.49 (m, 4H), 7.12–7.08 (m, 2H), 6.96 (d, *J* = 7.48 Hz, 2H), 6.92–6.87 (m, 3H), 6.37 (d, *J* = 7.52 Hz, 1H), 5.65 (d, *J* = 7.48 Hz, 2H), 5.37 (t, *J* = 8.76 Hz, 2H), 4.94 (d, *J* = 8.84 Hz, 2H). ^13^C NMR (100 MHz, CDCl_3_); δ 193.8, 152.3, 142.6, 137.7, 135.8, 134.8, 132.2, 131.8, 130.1, 129.4, 126.5, 125.8, 123.7, 120.5, 101.6, 65.2, 50.1. HRMS (EI): *m*/*z* calculated for C_17_H_11_Br_2_NOK^+^ [M/2 + K]^+^: 441.8833, found 441.0895.

#### 2.2.15 Compound **3o** (**122**)

Light orange solid, 74% yield. Mp: 148–149 °C. ^1^H NMR (400 MHz, CDCl_3_): δ 7.58–7.57 (m, 2H), 7.54–7.53 (m, 2H), 7.13–7.09 (m, 2H), 7.02–6.91 (m, 8H), 6.38 (d, *J* = 7.24 Hz, 2H), 5.66 (d, *J* = 7.48 Hz, 2H), 5.40 (d, *J* = 8.72 Hz, 2H), 4.74 (d, *J* = 8.8 Hz, 2H). ^13^C NMR (100 MHz, CDCl_3_): δ 188.3, 152.4, 142.6, 138.0, 134.3, 132.9, 131.7, 128.0, 127.1, 125.7, 123.5, 101.1, 67.6, 50.7, 29.7. HRMS (ESI): *m*/*z* calculated for C_30_H_23_N_2_O_2_S_2_^+^ [M + H]^+^: 507.1195, found 507.1201.

#### 2.2.16 Compound **3p** (**50Br**)

Light yellow solid, 94% yield. Mp: 115–116 °C. ^1^H NMR (400 MHz, CDCl_3_): δ 7.82 (d, *J* = 7.52 Hz, 4H), 7.55 (t, *J* = 7.52 Hz, 2H), 7.42 (t, *J* = 7.64 Hz, 4H), 7.32 (d, *J* = 7.88 Hz, 2H), 6.85 (d, *J* = 7.36 Hz, 2H), 6.74 (t, *J* = 7.76 Hz, 2H), 6.55 (d, *J* = 7.56 Hz, 2H), 5.95 (d, *J* = 7.64 Hz, 2H), 5.44 (d, *J* = 9 Hz, 2H), 5.07 (d, *J* = 8.8 Hz, 2H). ^13^C NMR (100 MHz, CDCl_3_): δ 193.9, 189.5, 172.1, 139.6, 135.8, 133.5, 132.1, 131.4, 129.1, 128.7, 128.7, 126.6, 126.0, 119.3, 100.0, 64.6, 50.1. HRMS (MALDI-TOF): *m*/*z* calcd for C_34_H_24_Br_2_N_2_O_2_ [M]^+^: 650.0205, found 650.0285.

#### 2.2.17 Compound **3q** (**103Br**)

Yellow solid, 34% yield. Mp: 177–178 °C. ^1^H NMR (400 MHz, CDCl_3_): δ 7.37–7.29 (m, 4H), 7.21–7.18 (m, 4H), 7.15 (t, *J* = 7.2 Hz, 2H), 6.92 (d, *J* = 7.3 Hz, 2H), 6.82–6.77 (m, 2H), 6.29 (d, *J* = 8.0 Hz, 2H), 5.81 (d, *J* = 7.6 Hz, 2H), 5.45 (m, *J* = 8.0 Hz, 2H) 4.87 (d, *J* = 9 Hz, 2H), 2.43 (s, 6H). ^13^C NMR (100 MHz, CDCl_3_): δ 197.7, 139.8, 138.7, 137.1, 132.2, 131.9, 131.6, 131.4, 128.8, 127.8, 126.5, 125.8, 125.5, 119.3, 99.5, 66.9, 50.1, 20.8. HRMS (MALDI-TOF): *m*/*z* calculated for C_36_H_28_Br_2_N_2_O_2_^+^ [M]^+^: 678.0518, found 678.0512.

#### 2.2.18 Compound **3r** (**91Br**)

Yellow solid, 21% yield. Mp: 145–146 °C. ^1^H NMR (400 MHz, CDCl_3_): δ 7.38 (s, 2H), 7.32 (t, *J* = 7.4 Hz, 6H), 7.08–7.05 (m, 2H), 6.84 (d, *J* = 7.36 Hz, 2H), 6.75 (t, *J* = 7.76 Hz, 2H), 6.53 (d, *J* = 7.72 Hz, 2H), 5.94 (d, *J* = 7.64 Hz, 2H), 5.43 (d, *J* = 8.84 Hz, 2H), 5.05 (d, *J* = 8.92 Hz, 2H), 3.83 (s, 6H). ^13^C NMR (100 MHz, CDCl_3_); δ 193.7, 159.9, 139.6, 137.1, 132.1, 129.6, 126.6, 126.0, 121.2, 120.2, 112.9, 100.0, 64.7, 58.4, 55.4, 50.2, 30.9,18.4. HRMS (ESI): *m*/*z* calculated for C_36_H_29_Br_2_N_2_O_4_^+^ [M + H]^+^: 711.0489, found 711.0494.

#### 2.2.19 Compound **3s** (**68Br**)

Yellow solid, 60% yield. Mp: 169–170 °C. ^1^H NMR (400 MHz, CDCl_3_): δ 7.71 (d, *J* = 8.16 Hz, 3H), 7.31 (d, *J* = 8.0 Hz, 3H), 7.20 (d, *J* = 8.1 Hz, 4H), 6.84 (d, *J* = 7.44 Hz, 2H), 6.73 (t, *J* = 7.7 Hz, 2H), 6.54 (d, *J* = 7.6 Hz, 2H), 5.92 (d, *J* = 7.6 Hz, 2H), 5.44 (d, *J* = 8.8 Hz, 2H), 5.04 (d, *J* = 8.8 Hz, 2H), 2.36 (s, 6H). ^13^C NMR (100 MHz, CDCl_3_): δ 193.5, 144.5, 139.7, 133.2, 132.0, 131.4, 129.3, 129.2, 128.8, 126.5, 126.0, 119.2, 99.8, 64.5, 50.1, 21.6. HRMS (ESI): *m*/*z* calculated for C_36_H_29_Br_2_N_2_O_2_^+^ [M + H]^+^: 679.0590, found 679.0596.

#### 2.2.20 Compound **3t** (**70Br**)

Pale yellow solid, 44% yield. Mp: 181–182 °C. ^1^H NMR (400 MHz, CDCl_3_): δ 7.79 (d, *J* = 8.8 Hz, 4H), 7.31 (d, *J* = 8 Hz, 2H), 6.87–6.81 (m, 6H) 6.73 (t, *J* = 7.7 Hz, 2H), 6.53 (d, *J* = 7.68 Hz, 2H), 5.91 (d, *J* = 7.56 Hz, 2H), 5.46 (d, *J* = 8.92 Hz, 2H), 5.03 (d, *J* = 8.8 Hz, 2H), 3.83 (s, 6H). ^13^C NMR (100 MHz, CDCl_3_): δ 192.5, 163.8, 148.0, 139.8, 132.0, 131.5, 131.1, 129.2, 128.8, 126.5, 126.1, 119.1, 113.8, 99.0, 64.5, 55.5, 50.4. HRMS (ESI): *m*/*z* calculated for C_18_H_15_BrNO_2_^2+^ [M/2 + H]^2+^: 356.0275, found 356.0294.

#### 2.2.21 Compound **3u** (**122Br**)

Light yellow solid, 44% yield. Mp: 176–177 °C. ^1^H NMR (400 MHz, CDCl_3_): δ 7.62 (d, *J* = 4.8 Hz, 2H), 7.58 (d, *J* = 3.8 Hz, 2H), 7.35 (d, *J* = 7.9 Hz, 2H) 7.06 (t, *J* = 4.4 Hz, 2H), 6.96 (d, *J* = 7.4 Hz, 2H), 6.80 (t, *J* = 7.7 Hz, 2H), 6.50 (d, *J* = 7.6 Hz, 2H), 5.98 (d, *J* = 7.6 Hz, 2H), 5.39 (d, *J* = 8.7 Hz, 2H), 4.78 (d, *J* = 8.7 Hz, 2H). ^13^C NMR (100 MHz, CDCl_3_): δ 187.5, 142.2, 139.6, 134.7, 133.2, 132.2, 131.3, 129.0, 128.2, 126.7, 126.3, 119.3, 100.1, 67.1, 50.7. HRMS (MALDI-TOF): *m*/*z* calcd for C_30_H_20_Br_2_N_2_O_2_S_2_^+^ [M]^+^: 661.9333, found 661.9306.

#### 2.2.22 Compound **3v** (**115Br**)

Light orange solid, 56% yield. Mp: 156–157 °C. ^1^H NMR (400 MHz, CDCl_3_): δ 7.91 (d, *J* = 8.3 Hz, 3H), 7.63 (d, *J* = 8.4 Hz, 4H), 7.59 (t, *J* = 7.1 Hz, 4H), 7.48–7.38 (m, 7H), 7.33 (t, *J* = 7.3 Hz, 2H), 6.88 (d, *J* = 7.4 Hz, 2H), 6.76 (t, *J* = 7.7 Hz, 2H), 6.60 (d, *J* = 7.6 Hz, 2H), 5.98 (d, *J* = 7.6 Hz, 2H), 5.48 (d, *J* = 8.8 Hz, 2H), 5.11 (d, *J* = 8.8 Hz, 2H). ^13^C NMR (100 MHz, CDCl_3_): δ 193.4, 146.3. 139.7, 139.6, 134.4, 132.1, 131.4, 130.5, 129.3, 129.1, 128.9, 128.3, 128.0, 127.3, 127.2, 127.0, 126.7, 126.1, 119.3, 100.1, 64.8, 50.2. HRMS (EI): *m*/*z* calculated for C_46_H_33_Br_2_N_2_O_2_^+^ [M + H]^+^: 803.0898, found 803.0909.

### 2.3 X-ray Analysis of the Synthesized Compounds

Single-crystal X-ray data were acquired through a slow evaporation technique utilizing dichloromethane with ethyl acetate as the solvent system at room temperature. X-ray reflections were recorded at 200 K on the single crystals using Mo Kα X-radiation (λ = 0.71073 Å) with a Bruker Kappa APEX-II diffractometer. The crystal structures were solved and refined using SHELX-97. Additional information regarding the data collection and refinement parameters for the crystals can be found in the Supporting Information Tables S2–S6.

### 2.4 Antivirus EC_50_ and Cytotoxicity CC_50_ Measurements

The antiviral EC_50_ values of the synthesized compounds against SARS-CoV-2 were determined by using the plaque reduction assay as previously described.^26,43^ For the assay, VeroE6 cells were seeded into a 24-well culture plate in Dulbecco’s modified Eagle’s medium (DMEM) with 10% fetal bovine serum (FBS) and antibiotics 1 day before the infection. VeroE6 cells were infected by SARS-CoV-2 delta virus (NTU92) at 50–100 pfu for 1 h at 37 °C. After removal of virus inoculum, the cells were washed once with phosphate-buffered saline (PBS) and overlaid with 1 mL overlay medium containing 1% methylcellulose for 5 days at 37 °C. After 5 days, the cells were fixed with 10% formalin overnight. After removal of the overlay medium, the cells were stained with 0.5% crystal violet, and the plaques were counted. The percentage of inhibition was calculated as [1 – (*V*_D_/*V*_C_)] × 100%, where *V*_D_ and *V*_C_ refer to the virus titer in the presence and absence of an inhibitor, respectively. The minimal concentration of an antiviral required to reduce the plaque number by 50% (EC_50_) was calculated by regression analysis of the dose–response curve generated from the plaque assay.

Cytotoxicity of the inhibitors was determined by using acid phosphatase assay. Briefly, VeroE6 cells were seeded onto a 96-well culture plate at a concentration of 2 × 10^4^ cells per well. Next day, the medium was removed, and each well was washed once with PBS before adding DMEM containing 2% FBS and different concentrations of an inhibitor. Next, DMEM containing 2 μg/mL TPCK-trypsin was added. After 1 h of incubation at 37 °C, the medium was removed, and cells were washed by PBS. Then, DMEM containing 2% FBS and different concentrations of an antiviral was added. After 3 days of incubation at 37 °C, the medium was removed, and each well was washed once with PBS. Next, buffer containing 0.1 M sodium acetate (pH = 5.0), 0.1% Triton X-100, and 5 mM *p*-nitrophenyl phosphate was added. After the mixture was incubated at 37 °C for 2 h, 1 N NaOH was added to stop the reaction. The absorbance was then determined by an ELISA reader (VERSAmax, Molecular Devices, Sunnyvale, CA) at a wavelength of 405 nm. The percentage of cytotoxicity was calculated using the following formula: cytotoxicity % = [1 – (*A*_t_/*A*_s_) × 100]%, where *A*_t_ and *A*_s_ refer to the absorbance of a tested substance and solvent control, respectively. The 50% cytotoxicity concentration (CC_50_) was defined as the concentration reducing 50% of cell viability. For each data point, the measurements were repeated three times to yield the averaged CC_50_ values and the standard deviation.

### 2.5 Test of the Synthesized Compounds on Inhibiting RBD:ACE2

The active antivirals were tested for inhibiting RBD:ACE2 interaction using the NanoBiT technology commercial kit from Promega (WI, USA) as reported previously.^26^ The IC_50_ values of the synthesized compounds against the target were determined from the concentration-dependent inhibition curves fitted with the equation: *A*(*I*) = *A*(0) × {1 – [*I*/(*I* + IC_50_)]} using GraphPad Prism software (v.9.4.0).

### 2.6 Molecular Docking

Molecular docking studies were carried out using the iGEMDOCK software available at http://gemdock.life.nctu.edu.tw/dock/igemdock.php(44) to predict the binding interactions of compound **50** with RBD of the SARS-CoV-2 delta variant Spike protein. The three-dimensional (3D) structure of RBD (PDB ID: 7w92)^45^ was obtained from the RCSB Protein Data Bank (PDB, https://www.rcsb.org/). Before docking, all water molecules were removed from the structure. The RBD domain (residues 319 to 541) was isolated from the open state of the SARS-CoV-2 delta variant Spike protein. This binding site was prepared by assigning residue atom types and charges using the iGEMDOCK method. The 3D structure of compound **50** was created using the Molview Web site (https://molview.org/) and its structural data were converted to mol2 format with the Open Babel GUI software.^46^

For the molecular docking process, genetic algorithm (GA) settings were used with population sizes of 800, 80 generations, and 10 solutions. iGEMDOCK software was used to create profiles of protein–ligand interactions based on electrostatic (E), hydrogen-bonding (H), and van der Waals (V) forces. After docking, iGEMDOCK analyzed and ranked all poses based on their estimated binding energies. The binding energy scores were calculated as the sum of the interaction energies, and the pose with the lowest energy was considered the best fit for compound **50** in the target binding site.

### 2.7 Drug-likeness Analysis

The 3D structure of compound **50** was converted to an SMILES format using Open Babel GUI software. To evaluate the drug-likeness of compound **50**, the Lipinski rule of five was assessed using an online tool (http://www.scfbio-iitd.res.in/software/drugdesign/lipinski.jsp).^47,48^ The Absorption, Distribution, Metabolism, Excretion, and Toxicity (ADMET) profile of **50** was predicted through the pkCSM Web site (http://biosig.unimelb.edu.au/pkcsm/).^49^

## 3 Results

### 3.1 Synthesis of Tetrahydropyrazino[2,1-a:5,4-a′]diisoquinoline Derivatives from Isoquinoline and Various Phenacyl Bromides

**Scheme 1:**
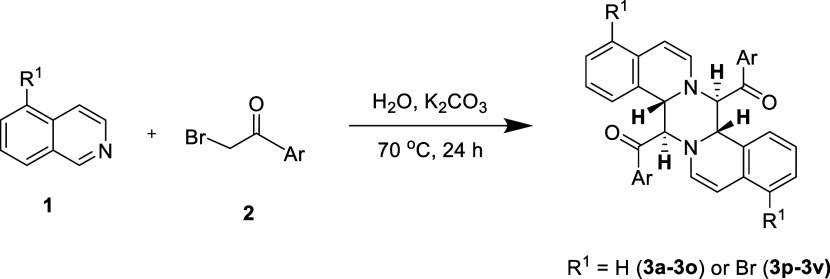
Synthesis of Tetrahydropyrazino[2,1-a:5,4-a′]diisoquinoline Derivatives from Isoquinoline or Bromoisoquinoline and Various Phenacyl Bromides under the Optimized Condition

To synthesize the target molecules, in the beginning, a mixture of isoquinoline **1** (1 mmol) and 2-bromoacetophenone **2** (1 mmol) with different solvents and different bases under different temperatures was tried. EtOH as solvent and K_2_CO_3_ as base at the temperature of 40 °C gave the yield of 86% for compound **3a**. For further optimization, we found using K_2_CO_3_ as base and H_2_O as solvent, at 70 °C was sufficient to give the product with a higher yield of 96% (Table 1). Therefore, our general synthetic scheme (Scheme 1) became a mixture of isoquinoline **1** (1 mmol) with R^1^ substituent H or Br, phenacyl bromide **2** (1 mmol), K_2_CO_3_ (1 mmol), and H_2_O (0.5 mL), and stirred at 70 °C for 24 h. Upon the consumption of isoquinoline **1** (monitored by TLC), the reaction mixtures were cooled to room temperature, filtered, concentrated, and purified by silica gel flash chromatography (Hex/EtOAc) to afford compounds **3a**–**3v**, each as a single diastereomer.

**Table 1:** Yields of **3a** Using the Reaction Conditions of 1 (1 mmol), 2 (1 mmol), K_2_CO_3_ (1 mmol), and H_2_O (0.5 mL), at Various Temperatures and Time Periods

entry	solvent	base	TEMP (°C)	*t* (h)	yield (%)
1	H_2_O	K_2_CO_3_	sonication	2	20
2	H_2_O	K_2_CO_3_	rt	12	30
3	H_2_O	K_2_CO_3_	40 °C	28	42
4	H_2_O	K_2_CO_3_	50 °C	28	58
5	H_2_O	K_2_CO_3_	70 °C	24	96
6	H_2_O	K_2_CO_3_	90 °C	24	96
7	H_2_O	K_2_CO_3_	100 °C	24	96

**Figure 2:**
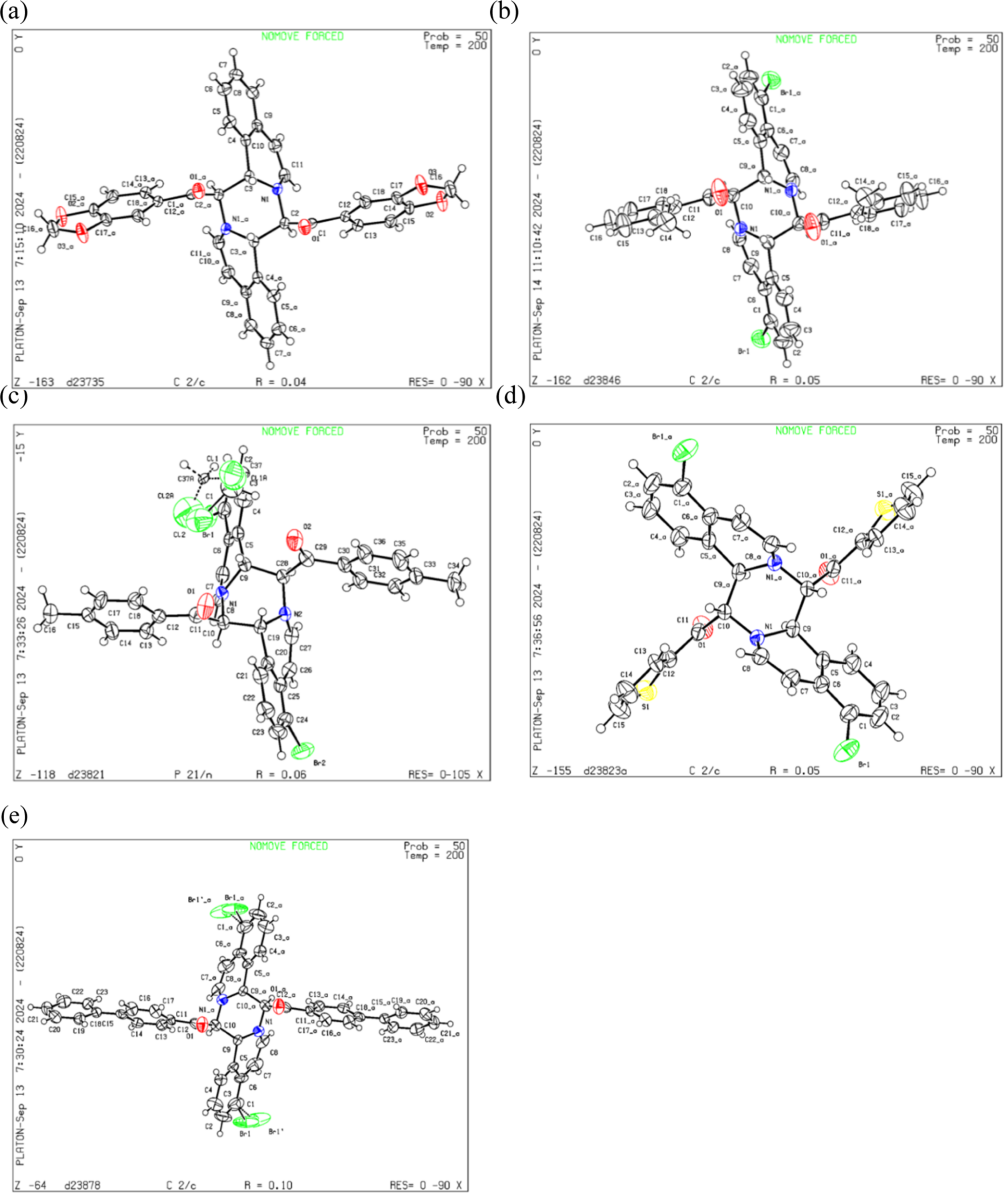
3-D structures of (A) **98**, (B) **103Br**, (C) **68Br**, (D) **122Br**, and (E) **115Br** determined from the diffraction data are summarized in Tables S2–S6. Their chemical structures are shown in Tables 2 and 3.

The chemical structures of the synthesized compounds were confirmed by NMR and MS spectral data. The NMR assignment was made on the substituent additivity rules, spectral characteristics of structurally related compounds, signal intensities, and multiplicities. ^13^C NMR spectra were used to prove the interpretation of the carbon resonances. The ^1^H and ^13^C NMR spectra for structural characterization are presented in Figure S1. The 3-D structures of five selected compounds were characterized by X-ray diffraction (the statistics are summarized in Tables S2–S6) and are shown in Figure 2.

The chemical structures and the yields of the synthesized compounds are summarized in Tables 2 and 3. The reaction of unsubstituted phenacyl bromide gave an excellent yield (96%) of the product (**3a**). However, *ortho*- or *para*-substituted phenacyl bromide substrates containing a halogen atom or methyl group produced the desired products in moderate yields (50–68%). On the other hand, the substrates containing bulky diphenyl or naphthyl groups afforded the corresponding tetrahydropyrazino[2,1-a:5,4-a′]diisoquinoline derivatives in lower yields (40–50%). Further, the substrate bearing methylenedioxy moiety furnished an excellent yield of the product **3m** (98%). The method was efficient, involving the reaction of the phenacyl bromide possessing thiophene group to produce the desired product **3o** in a good yield (74%). Next, we explored the methodology by replacing isoquinoline with bromoisoquinoline and various phenacyl bromides to obtain the corresponding tetrahydropyrazino[2,1-a:5,4-a′]diisoquinoline derivatives (**3p**–**3v**) in good to moderate yields. The substrate without a substituent or with a phenyl group on the benzene ring gave the best yield (94%).

**Table 2:**
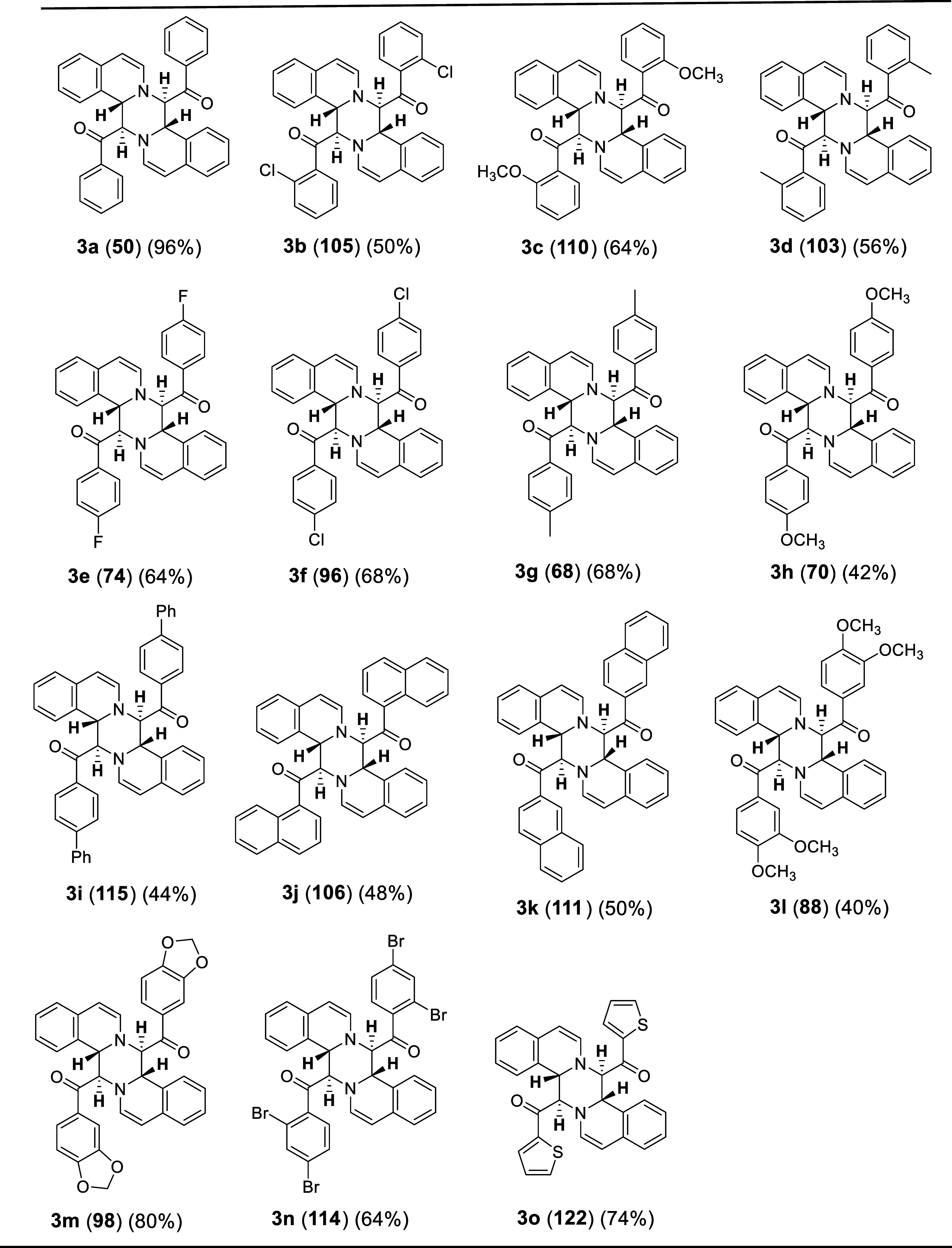
Structures and Yields of Tetrahydropyrazino[2,1-a:5,4-a′]diisoquinoline Derivatives Synthesized from Isoquinoline and Various Phenacyl Bromides^a^^,^^b^

a Reactions performed in 1 mmol scale.

b Isolated yields.

**Table 3:**
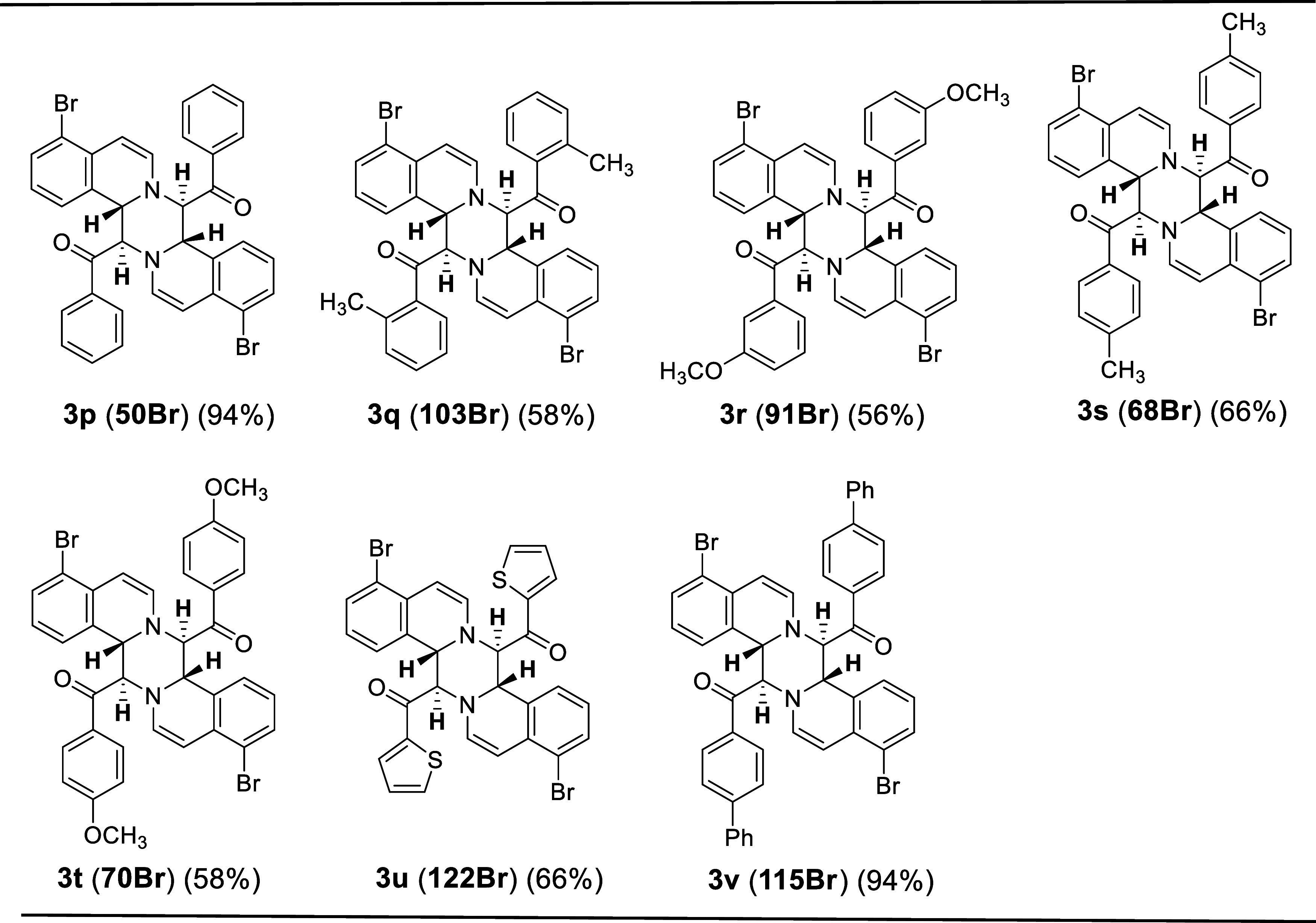
Structures and Yields of Tetrahydropyrazino[2,1-a:5,4-a′]dibromoisoquinoline Derivatives Synthesized from Bromoisoquinoline and Various Phenacyl Bromides^a^^,^^b^

a Reactions performed in 1 mmol scale.

b Isolated yields.

### 3.2 Antiviral Activities of Some Synthesized Compounds against SARS-CoV-2

**Figure 3:**
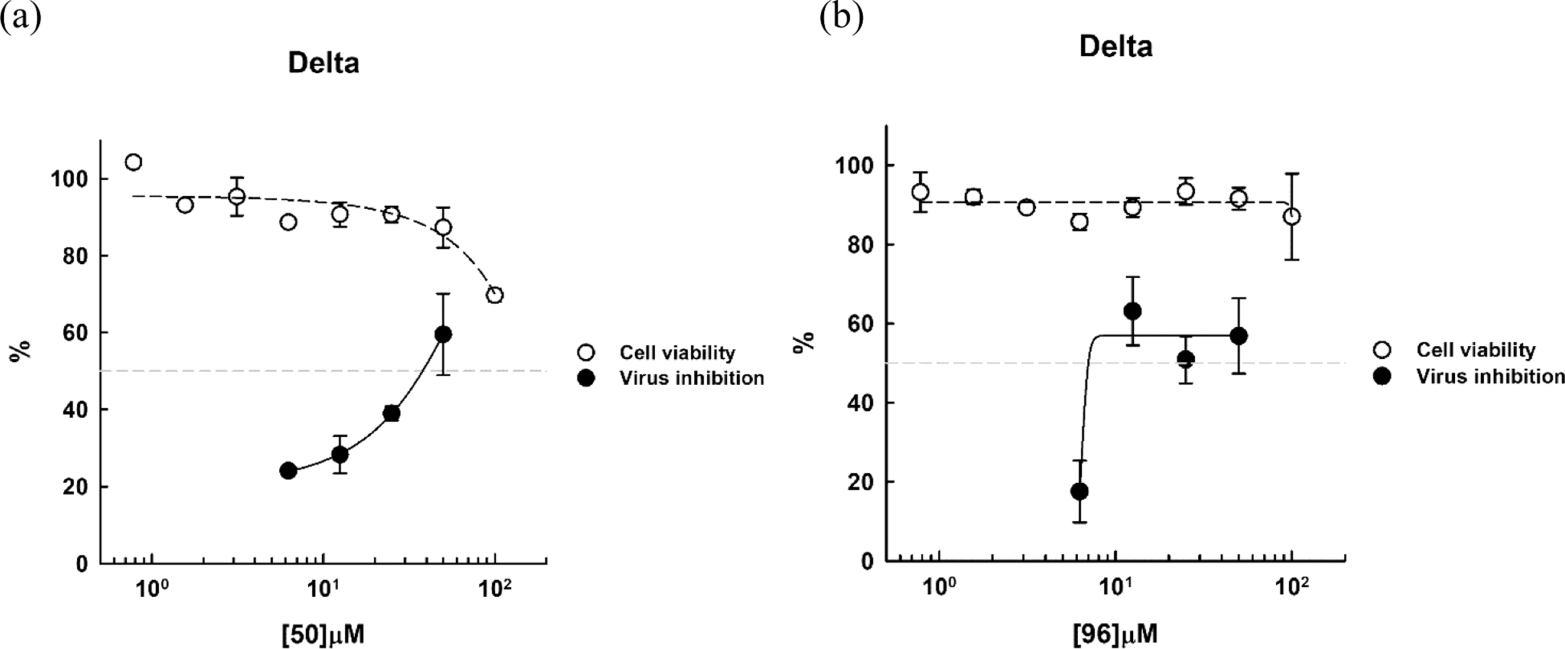
EC_50_ measurements of the antiviral activities of active compounds. (A, B) Inhibitor dose-dependent virus inhibition curves for measurements of EC_50_ values of the inhibitors, **50** and **96**, against the delta variant of SARS-CoV-2 infecting VeroE6 cells, are 26.5 ± 6.9 and 17.0 ± 3.7 μM, respectively. These curves were generated according to the plaque reduction assay data. Their CC_50_ values derived from the plots were >100 μM. All of the measurements were performed in triplicate to yield the averaged EC_50_ and standard deviations.

The synthesized compounds were screened for inhibiting infection of delta SARS-CoV-2 on VeroE6 host cells. Due to their weak anti-SARS-CoV-2 activities, EC_50_ measurements of some compounds could not be achieved. However, we found that **50** and **96** were repeatedly active under the entry treatment (preincubating with the virus). Their dose-dependent inhibition profiles shown in Figure 3 gave EC_50_ of 26.5 ± 6.9 and 17.0 ± 3.7 μM, respectively, without cytotoxicity (CC_50_ > 100 μM). The compounds were not active when added to the veroE6 cells after the cells were infected with SARS-CoV-2 (the postentry treatment) so that the compounds should inhibit the target(s) outside the cells for virus entry.

### 3.3 Evaluation of the Active Antivirals against RBD:ACE2 Involved in Virus Entry

**Figure 4:**
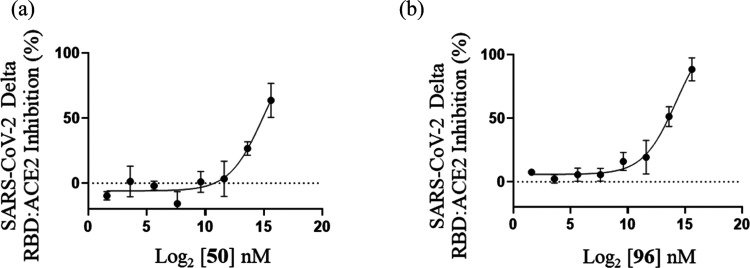
Inhibition of RBD:ACE2 by the compounds. (A, B) Percentages of RBD:ACE2 binding inhibition by increasing compound concentrations were measured with RBD from delta SARS-CoV-2 to yield IC_50_ values of 32.7 ± 8.9 and 10.4 ± 2.1 μM for compounds **50** and **96**, respectively. All of the measurements were performed in triplicate to yield the averaged IC_50_ values and standard deviations.

To understand the target(s) of the active antivirals, assays of the RBD:ACE2 interaction were carried out. The active compounds **50** and **96** displayed IC_50_ values of 32.7 ± 8.9 and 10.4 ± 2.1 μM, respectively, based on the dose-dependent inhibition profiles shown in Figure 4. These two antivirals showed no inhibition of the human proteases Furin, TMPRSS2, and Cathepsin L, which are also involved in virus entry (data not shown).

### 3.4 Binding Mode of the Inhibitor with RBD

**Figure 5:**
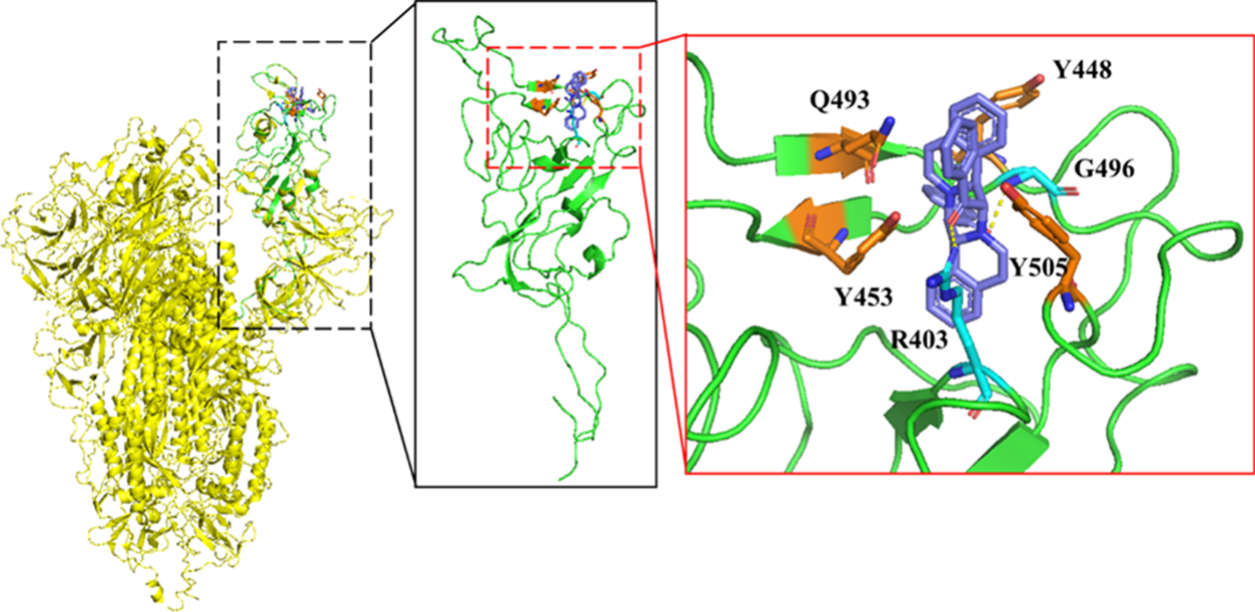
Binding mode of active antiviral compound **50** with RBD. Compound **50** was docked into the RBD of the delta-strain Spike protein (PDB: 7w92). Compound **50** is colored purple, and the residues involved in hydrogen bonding are highlighted in cyan, while those involved in van der Waals interactions are in orange. The trimeric Spike protein is depicted in yellow and the RBD is in green. In this model, compound **50** interacts with residues at the RBD:ACE2 interface, potentially interfering with the binding of the RBD to ACE2.

To elucidate the inhibition mechanism of compound **50** in virus entry, a docking study was conducted. **50** was docked into RBD of the SARS-CoV-2 delta variant Spike protein (PDB: 7w92), as shown in Figure 5. The binding energy of compound **50** with the RBD of the delta-strain Spike protein was estimated to be −93.3 kcal/mol. The analysis revealed that compound **50** forms van der Waals interactions with Tyr448, Tyr453, Gln493, and Tyr505 of the RBD. In the delta RBD:ACE2 complex, Tyr453, Gln493, and Tyr505 of RBD form hydrogen bonds with His34, Lys31, and Glu37 of ACE2, respectively. Additionally, an oxygen atom in one of the acetophenone groups forms a hydrogen bond with Gly496 of the RBD, which also forms a hydrogen bond with Lys353 of ACE2 in the delta RBD:ACE2 complex. Consequently, through interactions with these pivotal residues on the RBD, compound **50** potentially disrupts the RBD:ACE2.

### 3.5 Drug-likeness of the Inhibitor as Judged from Lipinski Rule of Five and ADMET Properties

According to the Lipinski rule of five, compound **50** fits several key criteria: its log *P* value is 5, the number of hydrogen bond acceptors is 4, fewer than 10, and the number of hydrogen bond donors is 0, fewer than 5. The molecular weight of compound **50** is 494 Da, which is smaller than the ideal limit of 500 Da. However, its molar refractivity is 146.607559, which is relatively surpassing the standard (between 40 and 130). These parameters suggested that compound **50** could be a promising candidate for drug development.

Moreover, the ADMET properties predicted *in silico* provided valuable insights into the potential of compounds for therapeutic use. These properties encompass absorption, distribution, metabolism, excretion, and toxicity, as summarized in Table S1 for compound **50**. For absorption, the Caco-2 permeability and human intestinal absorption (HIA) scores for compound **50** are relatively high, suggesting effective absorption in the human intestine. Regarding distribution, the ability of compound **50** to cross the blood-brain barrier (BBB) and penetrate the central nervous system (CNS), was supported by the predicted permeability values. Additionally, the volume of distribution at steady state (VDss) indicated a higher likelihood of tissue distribution. Metabolism prediction revealed that compound **50** may act as a substrate for CYP2D6 or CYP3A4. However, it was not predicted to inhibit CYP1A2, CYP2C19, CYP2C9, CYP2D6, or CYP3A4. In terms of excretion, compound **50** was unlikely to be a substrate for renal organic cation transporter-2 (OCT2), suggesting minimal risk of contraindications related to renal transport. Toxicity predictions indicated that compound **50** is not mutagenic, does not inhibit the hERG I channel, and lacks a skin sensitization potential. Furthermore, the predicted maximum recommended tolerated dose (MRTD) of compound **50** exceeds the standard values, suggesting a favorable safety profile with no unacceptable side effects. Nonetheless, there was a potential concern for hepatotoxicity associated with compound **50**.

## 4 Discussion

Quinoline is an important building block found in biologically active natural products, pharmaceuticals, and materials science; hence, considerable efforts have been made to develop environmentally benign synthetic protocols, workup, and purification procedures for quinoline derivatives. The synthesis of tetrahydropyrazino[2,1-a:5,4-a′]diisoquinoline first appeared in early 1969 when authors observed this compound as a side product.^50^ Later, Kutsuma et al. synthesized tetrahydropyrazino[2,1-a:5,4-a′]diisoquinoline by the treatment of isoquinoline and phenacyl bromide in the presence of trimethylamine, but only a single compound was synthesized.^51^ Recently, Cui et al. have reported the synthesis of the hydrogenated pyrazino[2,1-*a*]isoquinoline derivatives from dihydroisoquinolines and various phenacyl bromides in the presence of base in acetonitrile solvent.^52^ To the best of our knowledge, there is no green methodology developed for accessing these derivatives. Further, the bioactivities of these derivatives have not been investigated. Hence, we developed a green synthetic protocol for tetrahydropyrazino[2,1-a:5,4-a′]diisoquinoline derivatives from isoquinoline and phenacyl bromides in water, as demonstrated here. Water is an inexpensive, naturally abundant, safe, nontoxic, and environmentally benign solvent; hence, the development of novel organic reactions in aqueous media has attracted tremendous attention from the chemical community.^42^ In addition, since many organic compounds are not soluble in water, the resulting products can be precipitated out from the aqueous reaction medium after the reactions. Consequently, the products can be easily isolated by simple filtration without tedious workup and purification procedures. Our initial studies focused on optimizing the reaction conditions to obtain better yields of the target molecules. In this regard, we chose isoquinoline and phenacyl bromides as model substrates and conducted the reactions at various temperatures in water. After obtaining the optimized conditions, we synthesized a variety of tetrahydropyrazino[2,1-a:5,4-a′]diisoquinoline derivatives from various substituted phenacyl bromides and isoquinoline substrates. Next, we explored the methodology by replacing isoquinoline with bromoisoquinoline and various phenacyl bromides to obtain the corresponding derivatives. In all reactions, the *trans*-diastereomer was obtained as a major product, and the other isomer was formed in trace amounts. However, a pure *trans*-isomer was isolated by column chromatography. This protocol has several additional advantages such as simple operation, broad substrate scope, good functional group tolerance, and easy product isolation.

The synthesized compounds were screened against SARS-CoV-2 targeting the RBD:ACE2 interaction as hinted from modeling. Our results indicate that the compounds **50** and **96** inhibiting RBD:ACE2 but not the human proteases showed anti-SARS-CoV-2 activities under the entry treatment (the compound was preincubated with the virus and included during virus infecting the host to avoid virus entry), but not under the postentry treatment (the compound was added after the host was infected with the virus to prevent viral replication). Therefore, the active compounds exert their antiviral activities by targeting RBD:ACE2 to block virus entry. As compared to berbamine hydrochloride, a cyclic bis-benzylisoquinoline alkaloid that could bind to the postfusion core of SARS-CoV-2 S2 subunit, thereby inhibiting SRAS-CoV-2 entry into VeroE6 host cells with an EC_50_ of 1.73 μM and a CC_50_ of 66.88 μM, respectively,^40^ our inhibitors **50** and **96** display lower efficacy (EC_50_ = 26.5 and 17.0 μM), but higher safety (CC_50_ > 100 μM). As isoquinoline and dihydroisoquinoline derivatives may have different reactivities and bioactivities, dihydroisoquinolines can be considered in future studies for the synthesis of natural product-like compounds, and the synthesized molecules could be further tested for drug discovery. Several reports on the synthesis of related hydrogenated products can be considered for future study.^53−55^

To understand how the compounds bind the RBD:ACE2 interface, computational modeling that plays a pivotal role in investigating the interactions between potential drug candidates and target proteins was utilized. Understanding these interactions is crucial, as it expedites the drug discovery process by identifying promising candidates with higher efficacy and lower side effects. Using compound **50** as an example, Figure 5 reveals the interactions between the compound with Spike’s RBD of SARS-CoV-2 delta strain. Based on X-ray analysis, all our synthesized compounds have a shape with a central tetrahydropyrazino ring and 4 extended edges containing different groups. The extended shape helps to block the interface of RBD and ACE2 as modeled.

Computational drug discovery, through methods such as molecular docking, is vital for investigating how molecules interact with protein targets. In this study, we employ *i*GEMDOCK software to facilitate steps such as design of inhibitors and postscreening analysis. *i*GEMDOCK is especially useful for postscreening analysis and inferring pharmacological interactions from the active inhibitors.^44^ Moreover, by examining the computation-based study of drug-likeliness and pharmacokinetics, compound **50** obeys the Lipinski rule of five and shows favorable ADMET profiles. However, further in vitro and in vivo studies are needed to validate its effectiveness and safety.

## Supporting Information Available

NMR spectra and X-ray statistics of the compounds as well as the predicted properties of **50** (PDF)

## Abbreviations

COVID-19: Coronavirus Disease 2019
SARS-CoV-2: Severe Acute Respiratory Syndrome Coronavirus-2
ACE2: angiotensin-converting enzyme 2
RBD: receptor binding domain
TMPRSS2: transmembrane protease serine 2
3CL^pro^: 3C-like protease
PL^pro^: papain-like protease
NSP: nonstructural protein
RdRp: RNA-dependent RNA polymerase
TLC: thin-layer chromatography
PBS: phosphate-buffered saline
DMEM: Dulbecco’s modified Eagle’s medium
FBS: fetal bovine serum
