# Supplementary material for: Green Synthesis of Tetrahydropyrazino[2,1-a:5,4-a′]diisoquinolines as SARS-CoV-2 Entry Inhibitors
Source: ACS Omega. 2024 Dec 20;10(1):1164–76. doi: 10.1021/acsomega.4c08640 (PMC11740144; doi:10.1021/acsomega.4c08640)
Supplement: Supplementary file 1 — ao4c08640_si_001.pdf [file ao4c08640_si_001.pdf]

# Green Synthesis of Tetrahydropyrazino[2,1-a:5,4-a']diisoquinolines as SARS-CoV-2 Entry Inhibitors

Sowndarya Palla[d], Srinivasa Rao Palla[b,c], Jia-Jin Liu[b], Tai-Ling Chao[e], Ting-Hui Lee[f], Veerababurao Kavala[d], I-Chen Liu[b], Lily Hui-Ching Wang[g], Sui-Yuan Chang[e,h], Ching-Fa Yao[d**], and Po-Huang Liang[a,b,c*]

[a]Institute of Biological Chemistry, Academia Sinica, Taipei 11529, Taiwan

[b]Institute of Biochemical Sciences, National Taiwan University, 10617, Taiwan

[c]Taiwan International Graduate Program, Academia Sinica, Taipei 11529, Taiwan

[d]Department of Chemistry, National Taiwan Normal University, Taipei 11677, Taiwan.

[e]Department of Clinical Laboratory Sciences and Medical Biotechnology, National Taiwan University, Taipei 10048, Taiwan

[f]Department of Life Science, National Tsing Hua University, Hsinchu 30013, Taiwan

[g]Institute of Molecular and Cellular Biology, National Tsing Hua University, Hsinchu 30013, Taiwan

[h]Department of Laboratory Medicine, National Taiwan University Hospital, Taipei 10002, Taiwan

*Corresponding author: E-mail: phliang@gate.sinica.edu.tw, Tel: +886-2-3366-4069, Fax: +886-2-2363-5038.

**Corresponding author. E-mail: cheyaocf@ntnu.edu.tw

## Supplementary Information

**Figure S1.** NMR spectra ($^1$H top and $^{13}$C bottom) of the synthesized compounds.

## 3a (50)

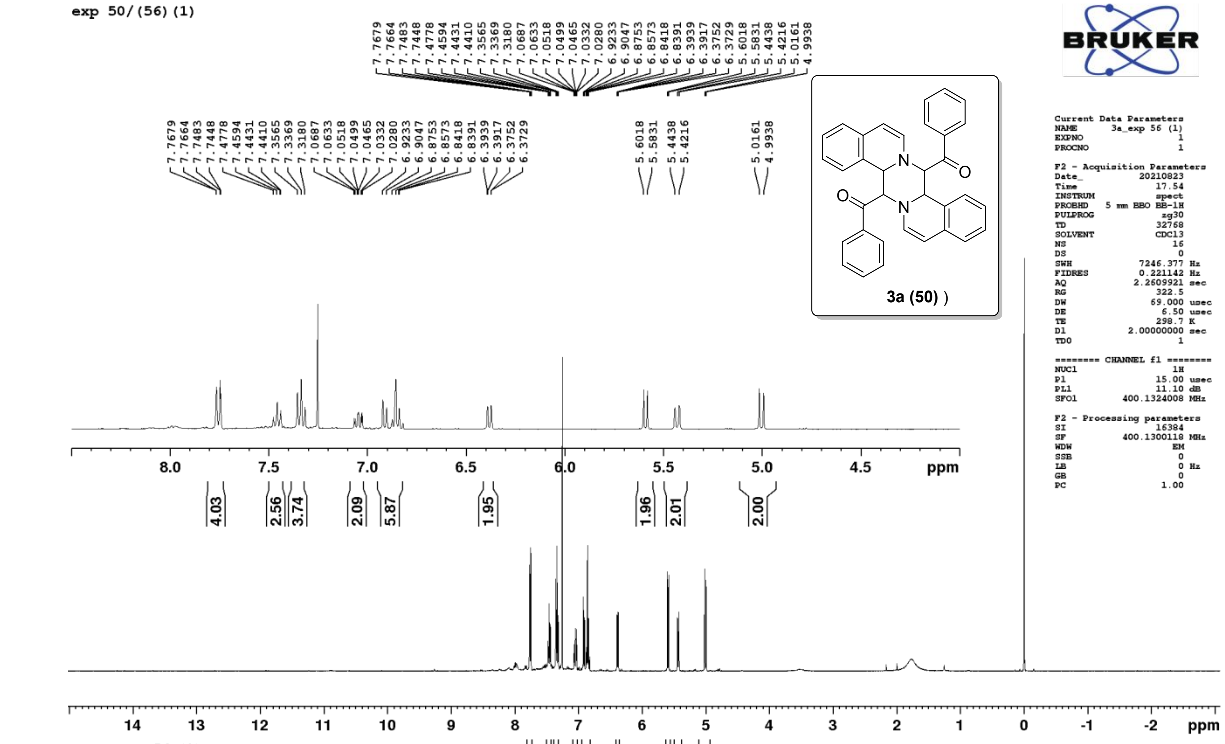


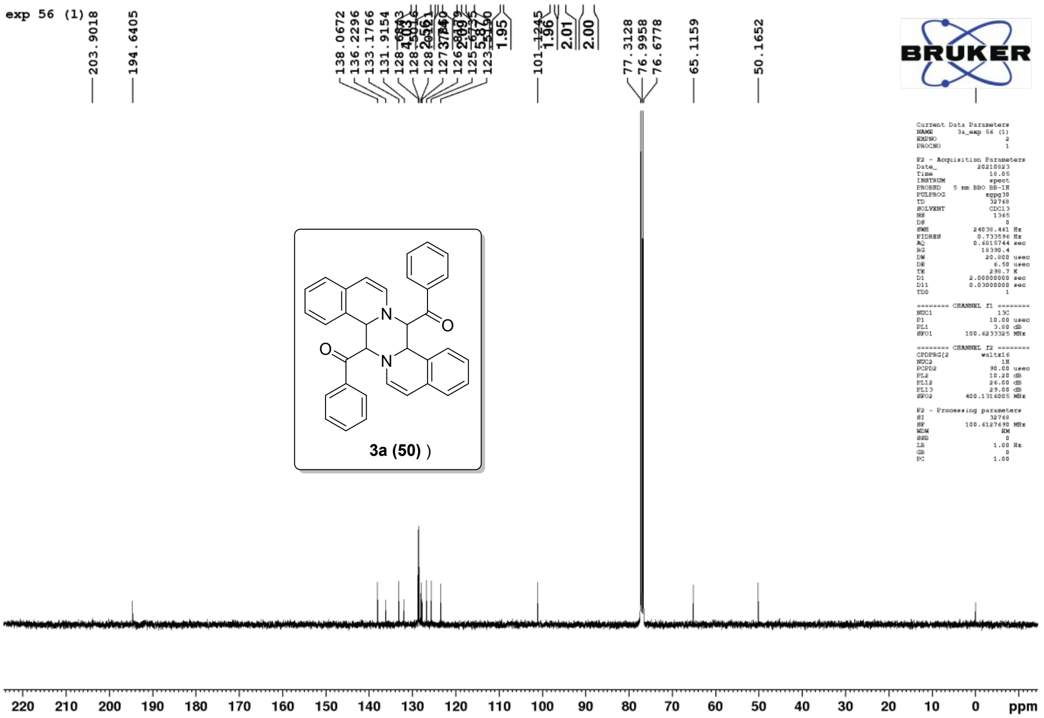

# 3b (105)

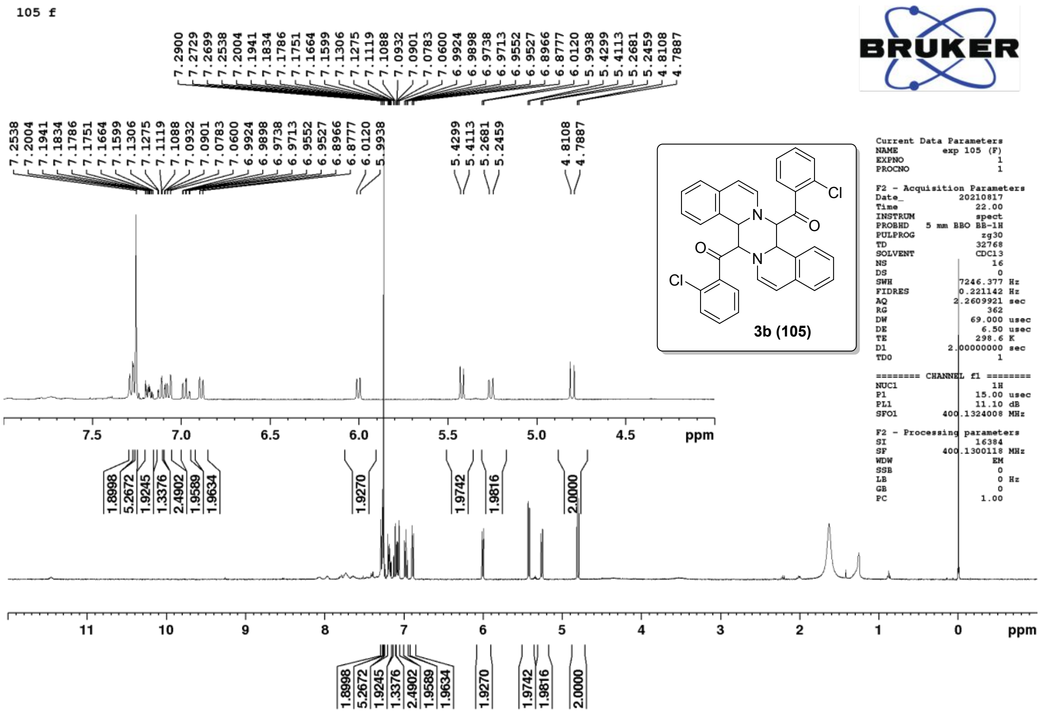
105 f
7.2900 7.2729 7.2699 7.2538 7.2004 7.1941 7.1834 7.1786 7.1751 7.1664 7.1599 7.1306 7.1275 7.1119 7.1088 7.0932 7.0901 7.0783 7.0600 6.9924 6.9898 6.9738 6.9713 6.9552 6.9527 6.8966 6.8777 6.0120 5.9938 5.4299 5.4113 5.2681 5.2459 4.8108 4.7887
3b (105)
ppm

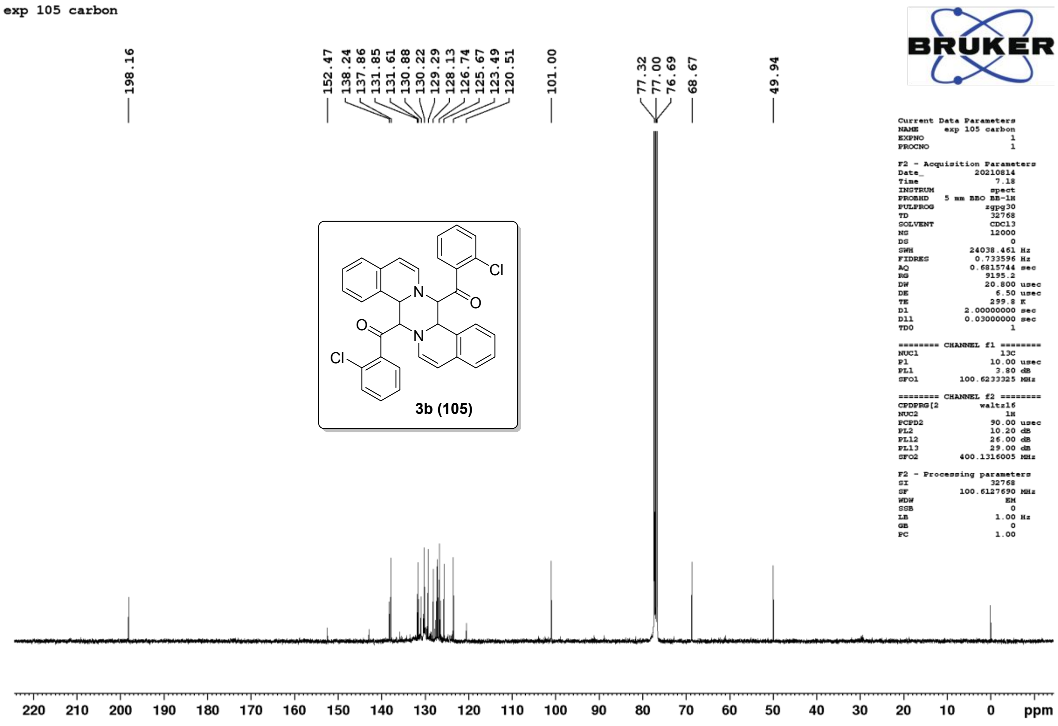
exp 105 carbon
198.16
152.47 138.24 137.86 131.85 131.61 130.88 130.22 129.29 128.13 126.74 125.67 123.49 120.51
101.00
77.32 77.00 76.69
68.67
49.94
3b (105)
ppm

# 3c (110)

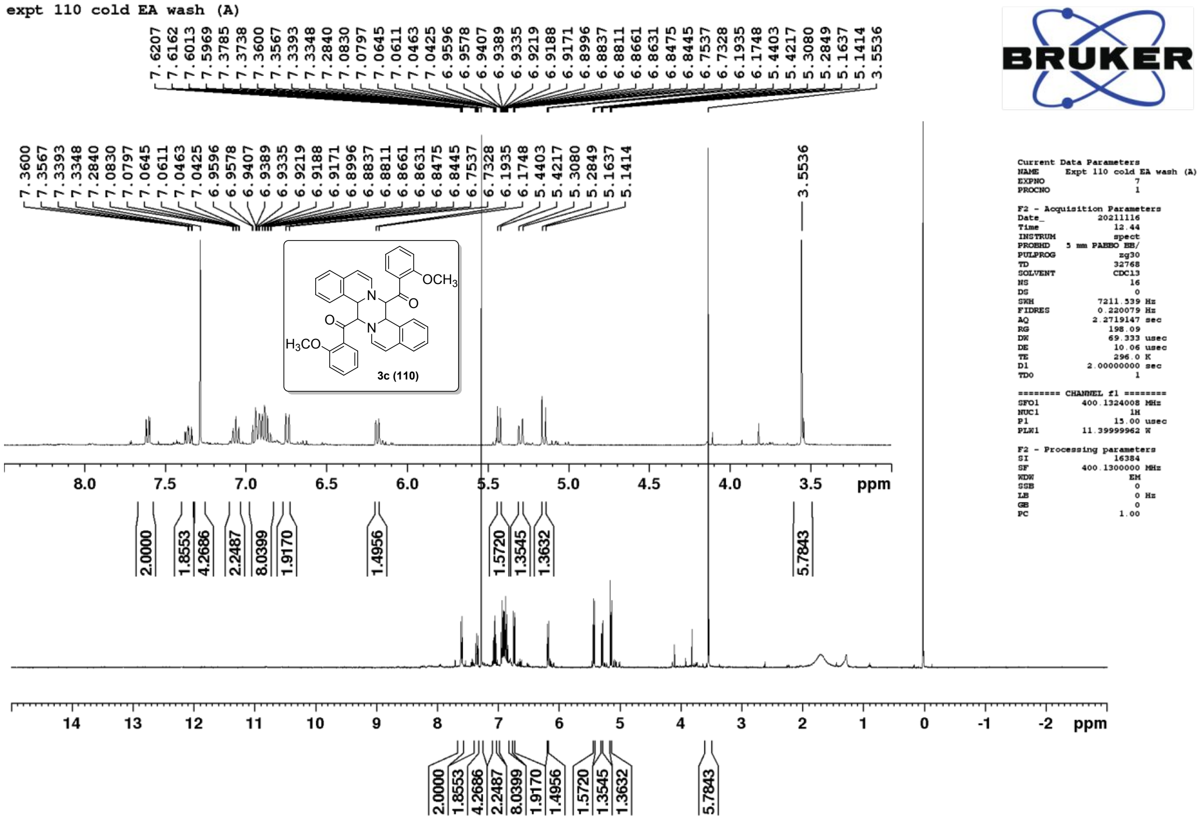
expt 110 cold EA wash (A)
7.6207 7.6162 7.6013 7.5969 7.3785 7.3738 7.3600 7.3567 7.3393 7.3348 7.2840 7.0830 7.0797 7.0645 7.0611 7.0463 7.0425 6.9596 6.9578 6.9407 6.9389 6.9335 6.9219 6.9188 6.9171 6.8996 6.8837 6.8811 6.8661 6.8631 6.8475 6.8445 6.7537 6.7328 6.1935 6.1748 5.4403 5.4217 5.3080 5.2849 5.1637 5.1414 3.5536
BRUKER
3c (110)
2.0000 1.8553 4.2686 2.2487 8.0399 1.9170 1.4956 1.5720 1.3545 1.3632 5.7843
ppm
Current Data Parameters
NAME Expt 110 cold EA wash (A)
EXPNO 7
PROCNO 1
F2 - Acquisition Parameters
Date_ 20211116
Time 12.44
INSTRUM spect
PROBHD 5 mm PABBO BB/
PULPROG zg30
TD 32768
SOLVENT CDCl3
NS 16
DS 0
SWH 7211.539 Hz
FIDRES 0.220079 Hz
AQ 2.2719147 sec
RG 198.09
DW 69.333 usec
DE 10.06 usec
TE 296.0 K
D1 2.00000000 sec
TD0 1
======== CHANNEL f1 ========
SFO1 400.1324008 MHz
NUC1 1H
P1 15.00 usec
PLW1 11.39999962 W
F2 - Processing parameters
SI 16384
SF 400.1300000 MHz
WDW EM
SSB 0
LB 0 Hz
GB 0
PC 1.00

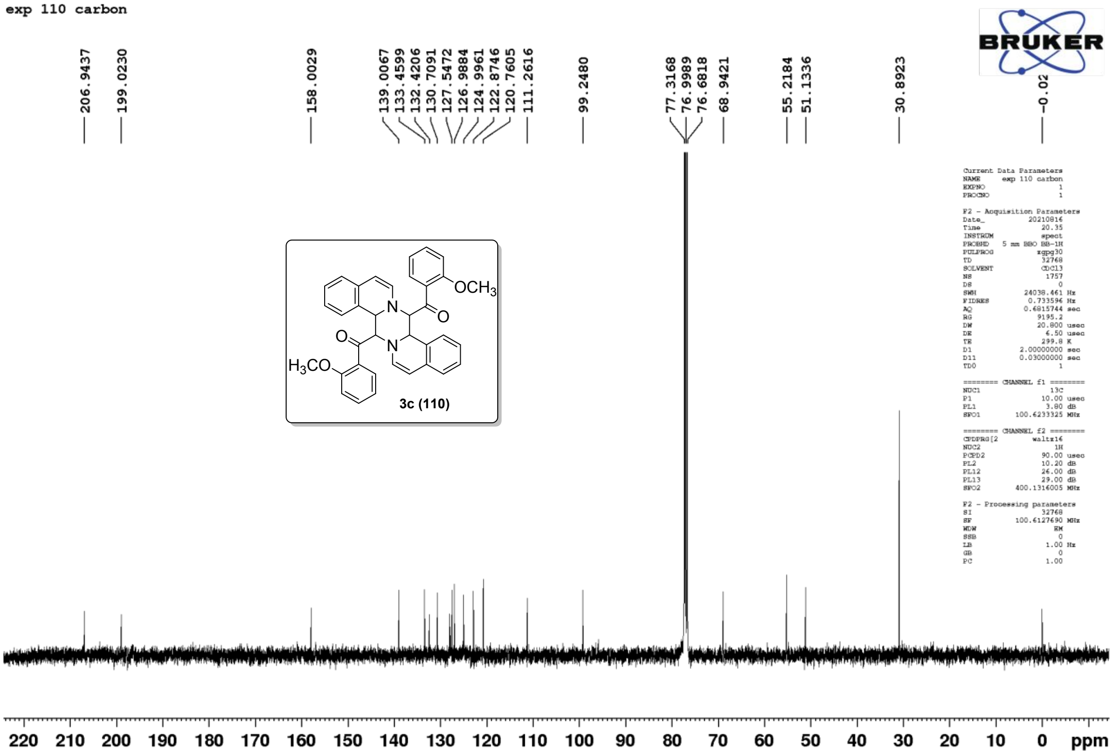
exp 110 carbon
206.9437 199.0230 158.0029 139.0067 133.4599 132.4206 130.7091 127.5472 126.9884 124.9961 122.8746 120.7605 111.2616 99.2480 77.3168 76.9989 76.6818 68.9421 55.2184 51.1336 30.8923 -0.02
BRUKER
3c (110)
ppm
Current Data Parameters
NAME exp 110 carbon
EXPNO 1
PROCNO 1
F2 - Acquisition Parameters
Date_ 20210816
Time 20.35
INSTRUM spect
PROBHD 5 mm BBO BB-1H
PULPROG zgpg30
TD 32768
SOLVENT CDCl3
NS 1757
DS 0
SWH 24038.461 Hz
FIDRES 0.733596 Hz
AQ 0.6815744 sec
RG 9195.2
DW 20.800 usec
DE 6.50 usec
TE 299.8 K
D1 2.00000000 sec
D11 0.03000000 sec
TD0 1
======== CHANNEL f1 ========
NUC1 13C
P1 10.00 usec
PL1 3.80 dB
SFO1 100.6233325 MHz
======== CHANNEL f2 ========
CPDPRG[2 waltz16
NUC2 1H
PCPD2 90.00 usec
PL2 10.20 dB
PL12 26.00 dB
PL13 29.00 dB
SFO2 400.1316005 MHz
F2 - Processing parameters
SI 32768
SF 100.6127690 MHz
WDW EM
SSB 0
LB 1.00 Hz
GB 0
PC 1.00

# 3d (103)

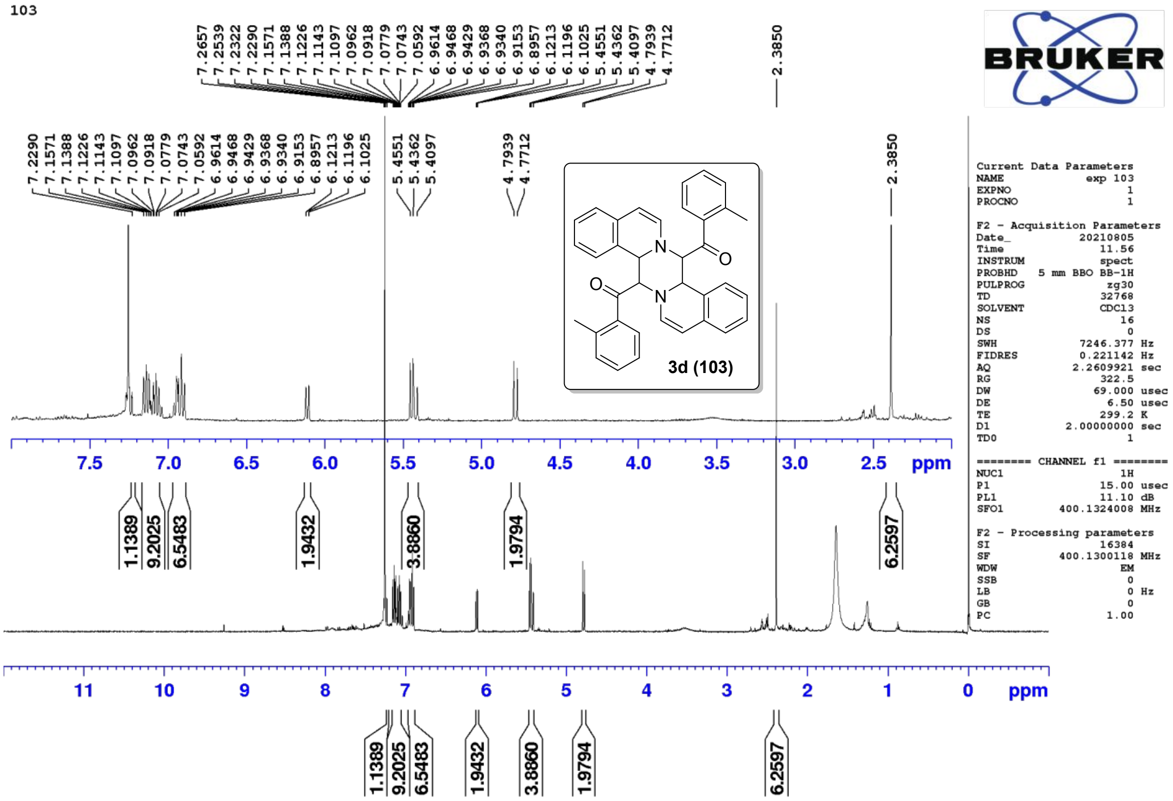
103
7.2657 7.2539 7.2322 7.2290 7.1571 7.1388 7.1226 7.1143 7.1097 7.0962 7.0918 7.0779 7.0743 7.0592 6.9614 6.9468 6.9429 6.9368 6.9340 6.9153 6.8957 6.1213 6.1196 6.1025 5.4551 5.4362 5.4097 4.7939 4.7712
2.3850
BRUKER
3d (103)
1.1389 9.2025 6.5483 1.9432 3.8860 1.9794 6.2597
ppm
Current Data Parameters
NAME exp 103
EXPNO 1
PROCNO 1
F2 - Acquisition Parameters
Date_ 20210805
Time 11.56
INSTRUM spect
PROBHD 5 mm BBO BB-1H
PULPROG zg30
TD 32768
SOLVENT CDCl3
NS 16
DS 0
SWH 7246.377 Hz
FIDRES 0.221142 Hz
AQ 2.2609921 sec
RG 322.5
DW 69.000 usec
DE 6.50 usec
TE 299.2 K
D1 2.00000000 sec
TD0 1
======== CHANNEL f1 ========
NUC1 1H
P1 15.00 usec
PL1 11.10 dB
SFO1 400.1324008 MHz
F2 - Processing parameters
SI 16384
SF 400.1300118 MHz
WDW EM
SSB 0
LB 0 Hz
GB 0
PC 1.00

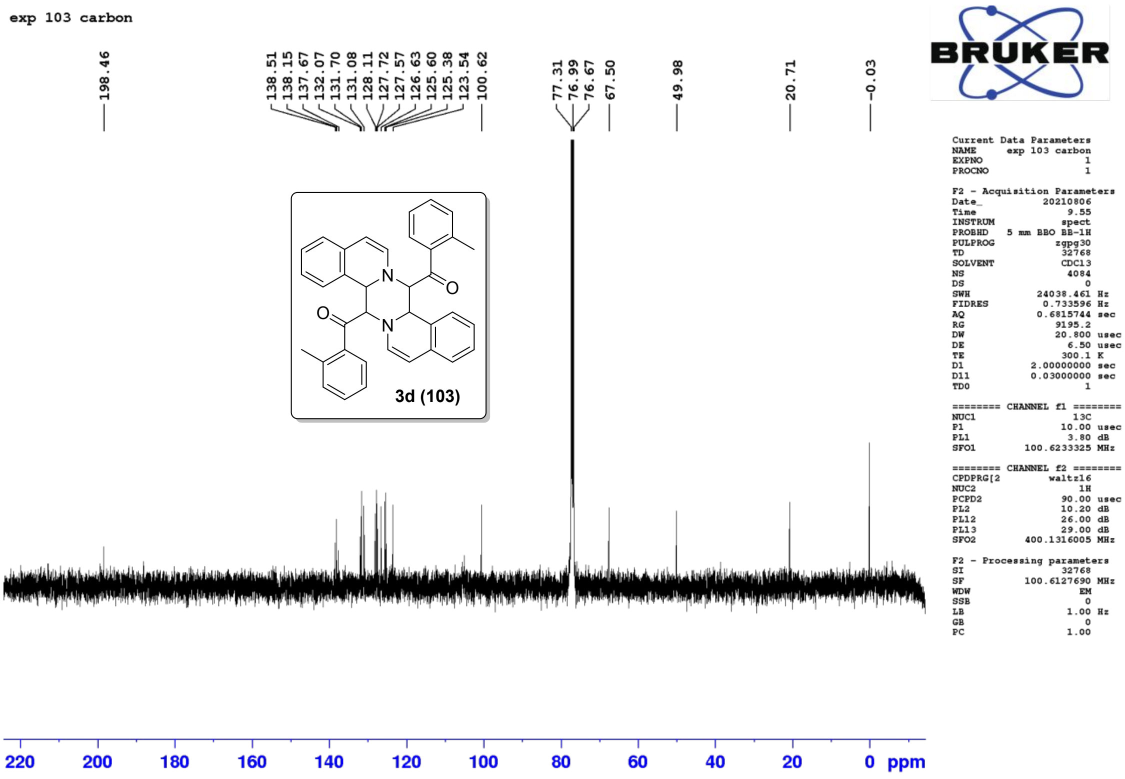
exp 103 carbon
198.46
138.51 138.15 137.67 132.07 131.70 131.08 128.11 127.72 127.57 126.63 125.60 125.38 123.54 100.62
77.31 76.99 76.67 67.50
49.98
20.71
-0.03
BRUKER
3d (103)
ppm
Current Data Parameters
NAME exp 103 carbon
EXPNO 1
PROCNO 1
F2 - Acquisition Parameters
Date_ 20210806
Time 9.55
INSTRUM spect
PROBHD 5 mm BBO BB-1H
PULPROG zgpg30
TD 32768
SOLVENT CDCl3
NS 4084
DS 0
SWH 24038.461 Hz
FIDRES 0.733596 Hz
AQ 0.6815744 sec
RG 9195.2
DW 20.800 usec
DE 6.50 usec
TE 300.1 K
D1 2.00000000 sec
D11 0.03000000 sec
TD0 1
======== CHANNEL f1 ========
NUC1 13C
P1 10.00 usec
PL1 3.80 dB
SFO1 100.6233325 MHz
======== CHANNEL f2 ========
CPDPRG[2 waltz16
NUC2 1H
PCPD2 90.00 usec
PL2 10.20 dB
PL12 26.00 dB
PL13 29.00 dB
SFO2 400.1316005 MHz
F2 - Processing parameters
SI 32768
SF 100.6127690 MHz
WDW EM
SSB 0
LB 1.00 Hz
GB 0
PC 1.00

# 3e (74)

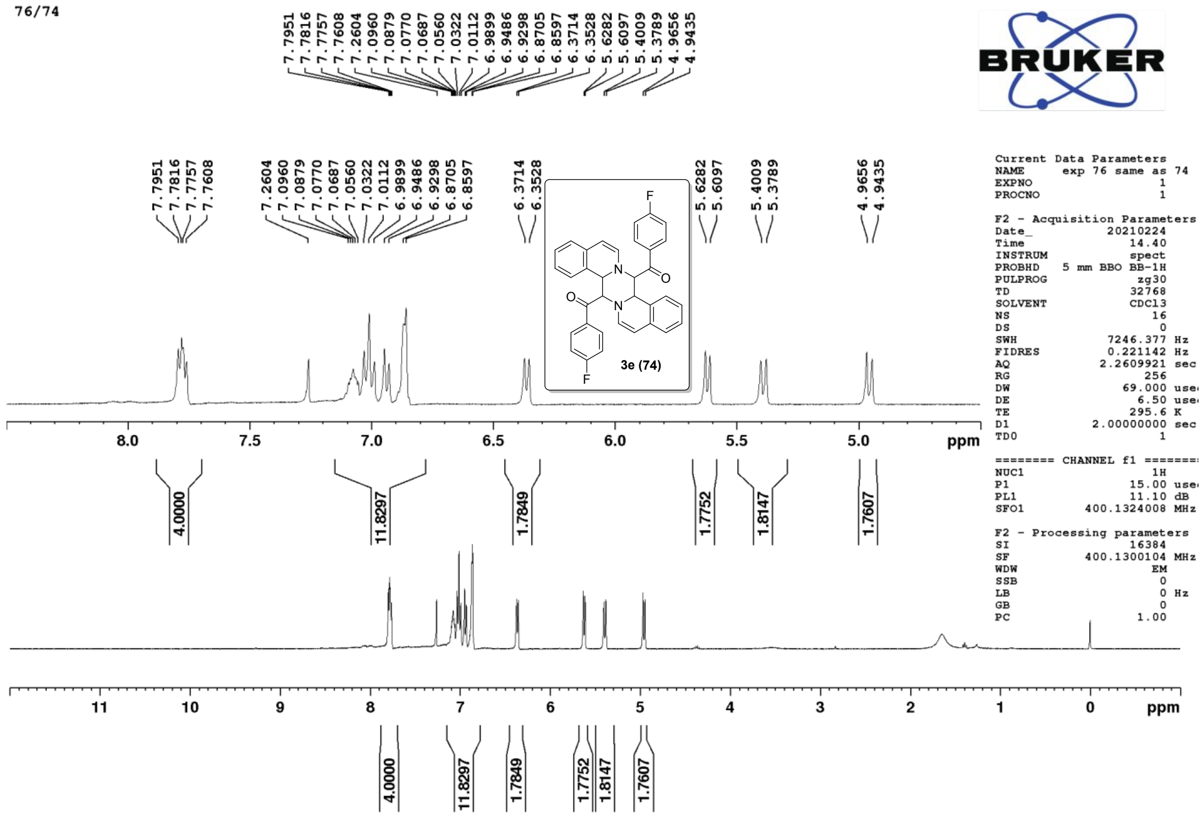
76/74
7.7951
7.7816
7.7757
7.7608
7.2604
7.0960
7.0879
7.0770
7.0687
7.0560
7.0322
7.0112
6.9899
6.9486
6.9298
6.8705
6.8597
6.3714
6.3528
5.6282
5.6097
5.4009
5.3789
4.9656
4.9435
3e (74)
F
O
N
ppm
4.0000
11.8297
1.7849
1.7752
1.8147
1.7607
Current Data Parameters
NAME exp 76 same as 74
EXPNO 1
PROCNO 1
F2 - Acquisition Parameters
Date_ 20210224
Time 14.40
INSTRUM spect
PROBHD 5 mm BBO BB-1H
PULPROG zg30
TD 32768
SOLVENT CDCl3
NS 16
DS 0
SWH 7246.377 Hz
FIDRES 0.221142 Hz
AQ 2.2609921 sec
RG 256
DW 69.000 usec
DE 6.50 usec
TE 295.6 K
D1 2.00000000 sec
TD0 1
======== CHANNEL f1 ========
NUC1 1H
P1 15.00 usec
PL1 11.10 dB
SFO1 400.1324008 MHz
F2 - Processing parameters
SI 16384
SF 400.1300104 MHz
WDW EM
SSB 0
LB 0 Hz
GB 0
PC 1.00

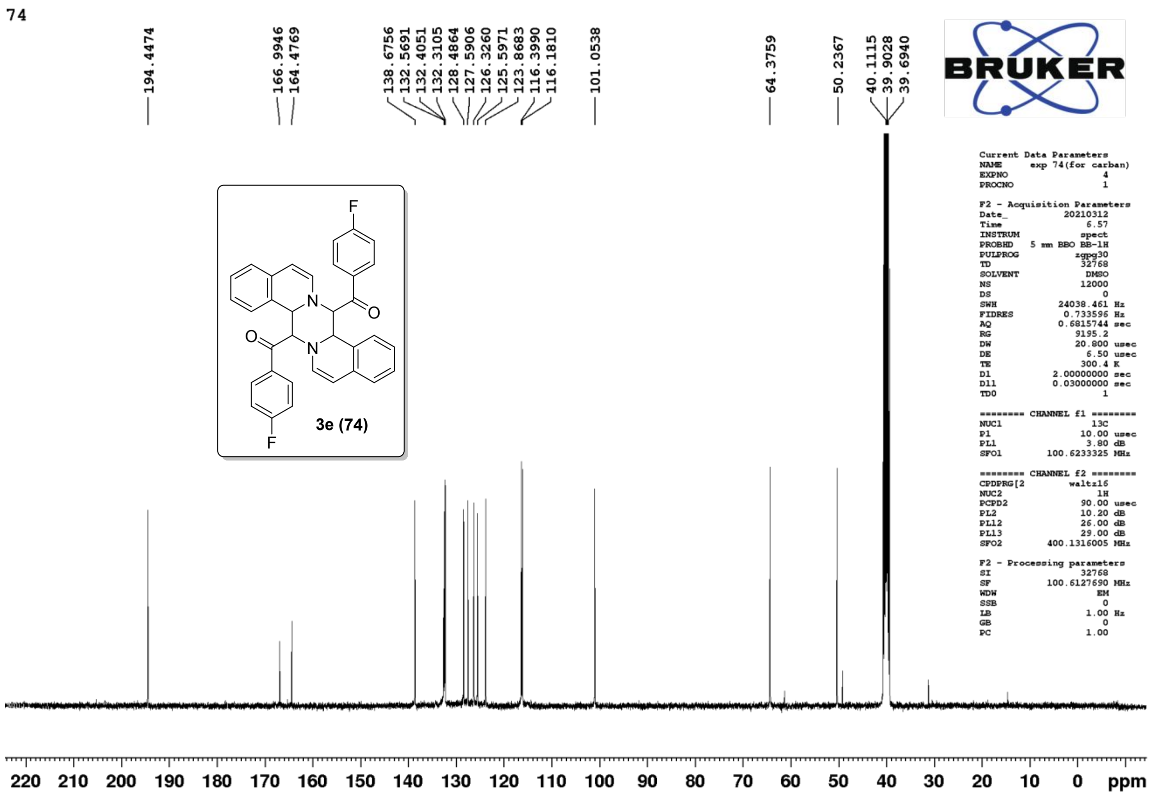
74
194.4474
166.9946
164.4769
138.6756
132.5691
132.4051
132.3105
128.4864
127.5906
126.3260
125.5971
123.8683
116.3990
116.1810
101.0538
64.3759
50.2367
40.1115
39.9028
39.6940
3e (74)
F
O
N
ppm
Current Data Parameters
NAME exp 74(for carban)
EXPNO 4
PROCNO 1
F2 - Acquisition Parameters
Date_ 20210312
Time 6.57
INSTRUM spect
PROBHD 5 mm BBO BB-1H
PULPROG zgpg30
TD 32768
SOLVENT DMSO
NS 12000
DS 0
SWH 24038.461 Hz
FIDRES 0.733596 Hz
AQ 0.6815744 sec
RG 9195.2
DW 20.800 usec
DE 6.50 usec
TE 300.4 K
D1 2.00000000 sec
D11 0.03000000 sec
TD0 1
======== CHANNEL f1 ========
NUC1 13C
P1 10.00 usec
PL1 3.80 dB
SFO1 100.6233325 MHz
======== CHANNEL f2 ========
CPDPRG[2 waltz16
NUC2 1H
PCPD2 90.00 usec
PL2 10.20 dB
PL12 26.00 dB
PL13 29.00 dB
SFO2 400.1316005 MHz
F2 - Processing parameters
SI 32768
SF 100.6127690 MHz
WDW EM
SSB 0
LB 1.00 Hz
GB 0
PC 1.00

**3f (96)**

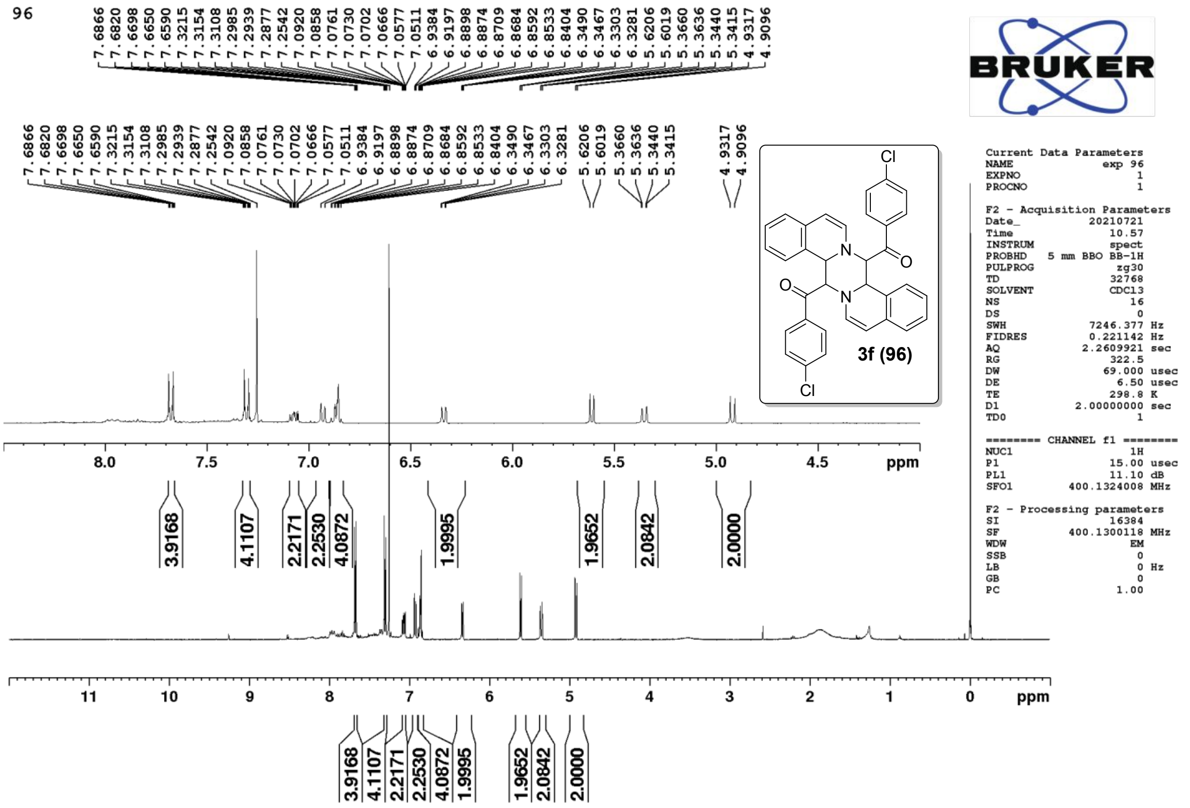

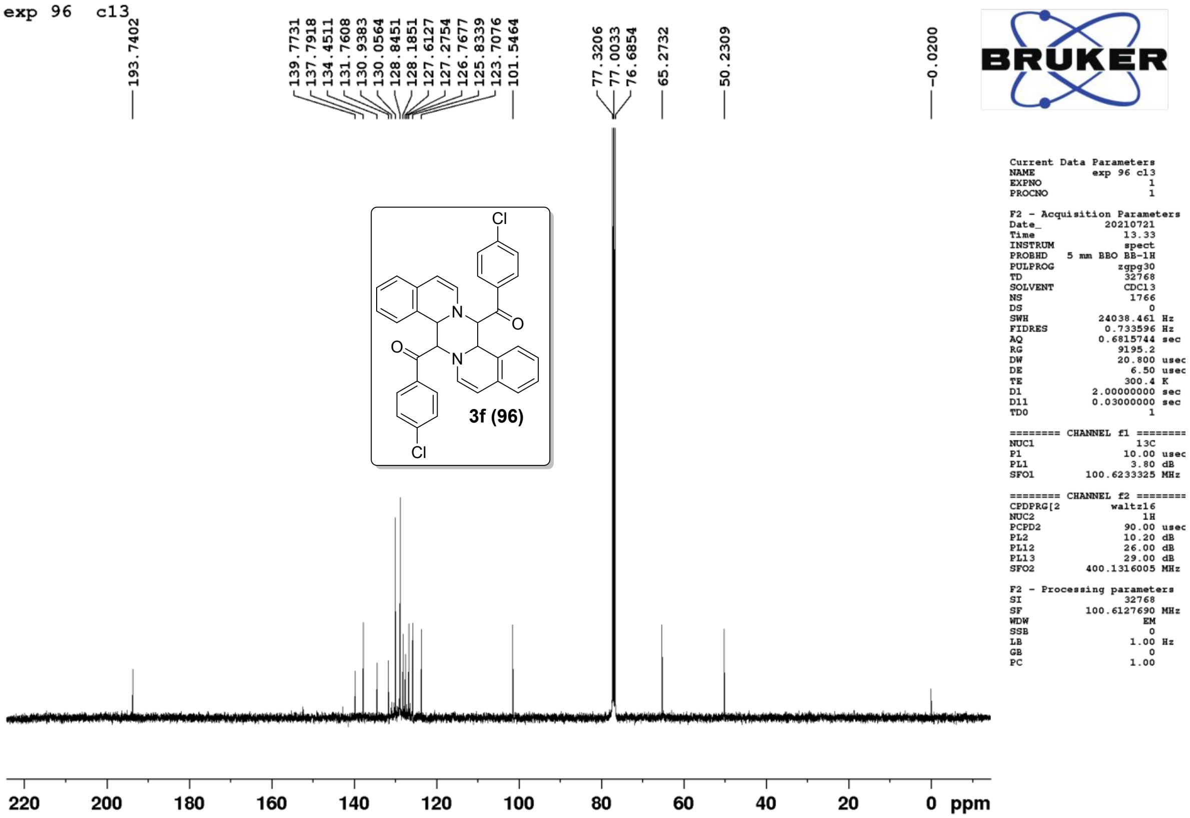

**3g (68)**

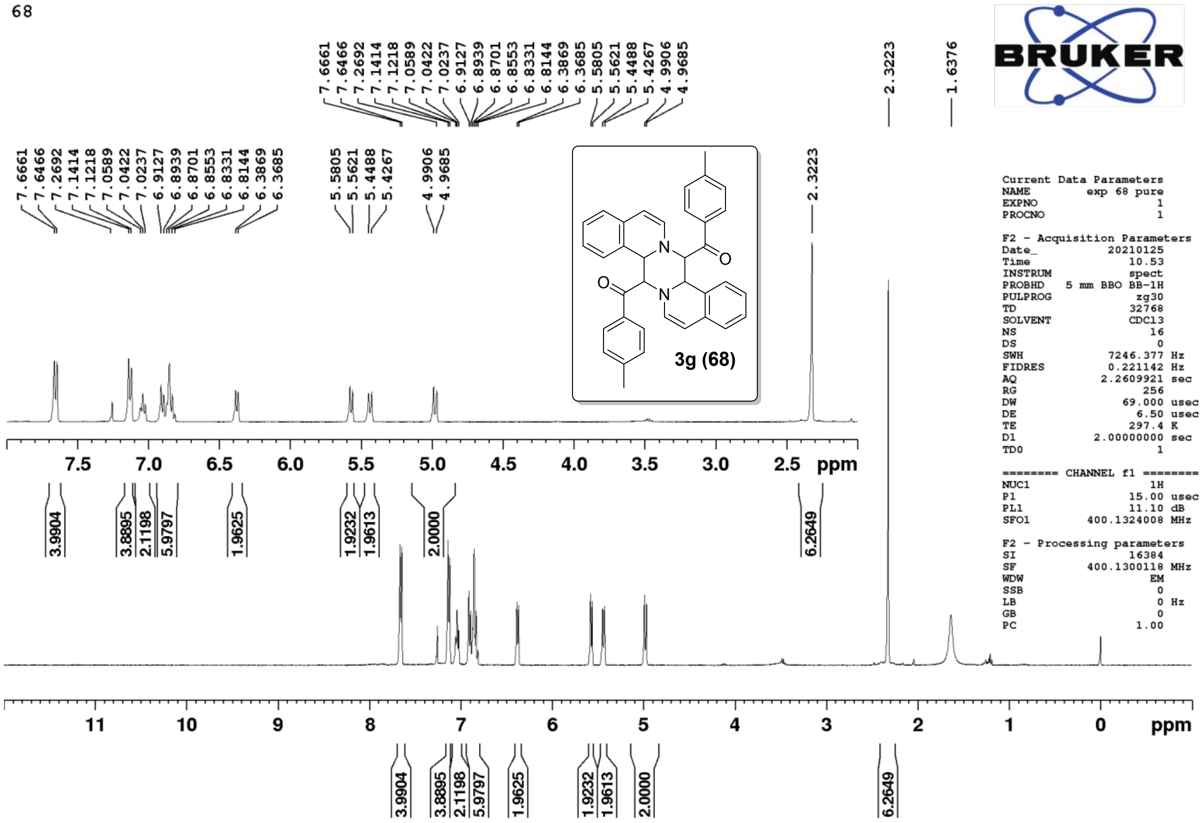
68
7.6661
7.6466
7.2692
7.1414
7.1218
7.0589
7.0422
7.0237
6.9127
6.8939
6.8701
6.8553
6.8331
6.8144
6.3869
6.3685
5.5805
5.5621
5.4488
5.4267
4.9906
4.9685
2.3223
1.6376
BRUKER
3g (68)
7.5 7.0 6.5 6.0 5.5 5.0 4.5 4.0 3.5 3.0 2.5 ppm
3.9904
3.8895
2.1198
5.9797
1.9625
1.9232
1.9613
2.0000
6.2649
11 10 9 8 7 6 5 4 3 2 1 0 ppm
Current Data Parameters
NAME exp 68 pure
EXPNO 1
PROCNO 1
F2 - Acquisition Parameters
Date_ 20210125
Time 10.53
INSTRUM spect
PROBHD 5 mm BBO BB-1H
PULPROG zg30
TD 32768
SOLVENT CDCl3
NS 16
DS 0
SWH 7246.377 Hz
FIDRES 0.221142 Hz
AQ 2.2609921 sec
RG 256
DW 69.000 usec
DE 6.50 usec
TE 297.4 K
D1 2.00000000 sec
TD0 1
======== CHANNEL f1 ========
NUC1 1H
P1 15.00 usec
PL1 11.10 dB
SFO1 400.1324008 MHz
F2 - Processing parameters
SI 16384
SF 400.1300118 MHz
WDW EM
SSB 0
LB 0 Hz
GB 0
PC 1.00

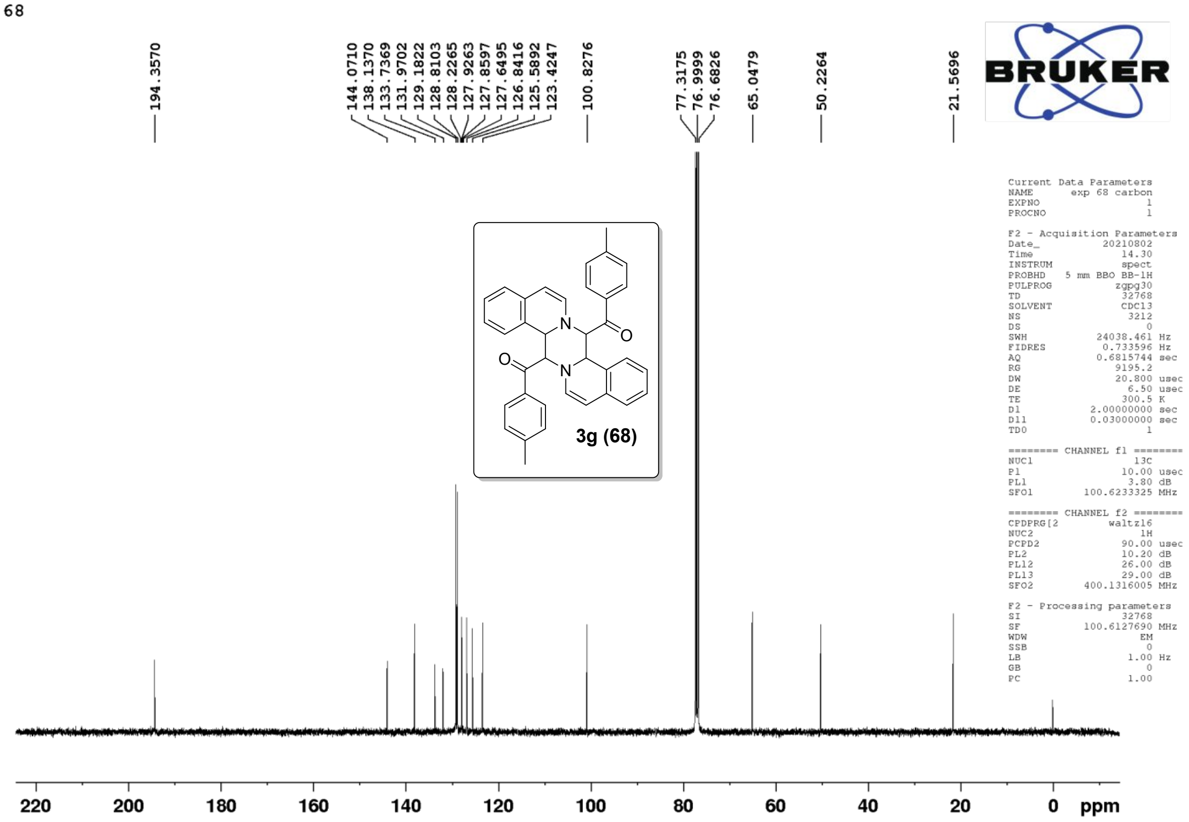
68
194.3570
144.0710
138.1370
133.7369
131.9702
129.1822
128.8103
128.2265
127.9263
127.8597
127.6495
126.8416
125.5892
123.4247
100.8276
77.3175
76.9999
76.6826
65.0479
50.2264
21.5696
BRUKER
3g (68)
Current Data Parameters
NAME exp 68 carbon
EXPNO 1
PROCNO 1
F2 - Acquisition Parameters
Date_ 20210802
Time 14.30
INSTRUM spect
PROBHD 5 mm BBO BB-1H
PULPROG zgpg30
TD 32768
SOLVENT CDCl3
NS 3212
DS 0
SWH 24038.461 Hz
FIDRES 0.733596 Hz
AQ 0.6815744 sec
RG 9195.2
DW 20.800 usec
DE 6.50 usec
TE 300.5 K
D1 2.00000000 sec
D11 0.03000000 sec
TD0 1
======== CHANNEL f1 ========
NUC1 13C
P1 10.00 usec
PL1 3.80 dB
SFO1 100.6233325 MHz
======== CHANNEL f2 ========
CPDPRG[2 waltz16
NUC2 1H
PCPD2 90.00 usec
PL2 10.20 dB
PL12 26.00 dB
PL13 29.00 dB
SFO2 400.1316005 MHz
F2 - Processing parameters
SI 32768
SF 100.6127690 MHz
WDW EM
SSB 0
LB 1.00 Hz
GB 0
PC 1.00
220 200 180 160 140 120 100 80 60 40 20 0 ppm

## 3h (70)

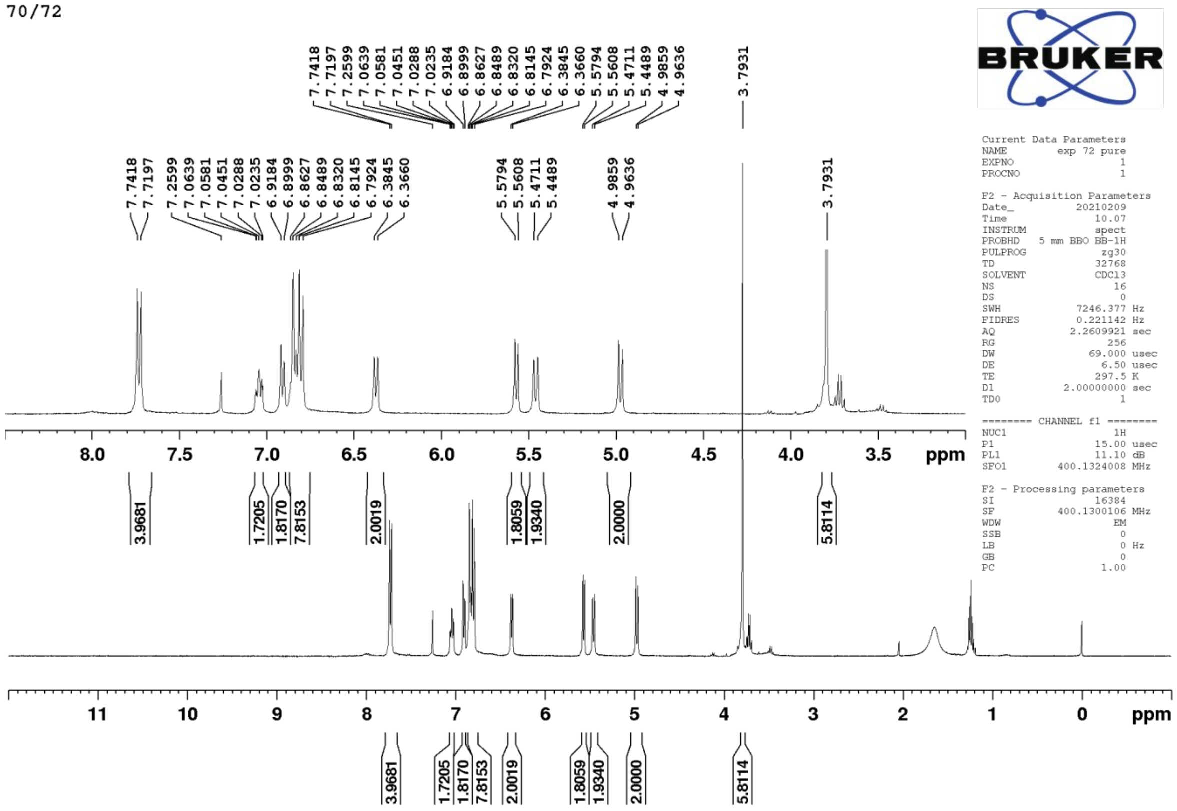
70/72
7.7418 7.7197 7.2599 7.0639 7.0581 7.0451 7.0288 7.0235 6.9184 6.8999 6.8627 6.8489 6.8320 6.8145 6.7924 6.3845 6.3660 5.5794 5.5608 5.4711 5.4489 4.9859 4.9636 3.7931
Current Data Parameters
NAME exp 72 pure
EXPNO 1
PROCNO 1
F2 - Acquisition Parameters
Date_ 20210209
Time 10.07
INSTRUM spect
PROBHD 5 mm BBO BB-1H
PULPROG zg30
TD 32768
SOLVENT CDCl3
NS 16
DS 0
SWH 7246.377 Hz
FIDRES 0.221142 Hz
AQ 2.2609921 sec
RG 256
DW 69.000 usec
DE 6.50 usec
TE 297.5 K
D1 2.00000000 sec
TD0 1
======== CHANNEL f1 ========
NUC1 1H
P1 15.00 usec
PL1 11.10 dB
SFO1 400.1324008 MHz
F2 - Processing parameters
SI 16384
SF 400.1300106 MHz
WDW EM
SSB 0
LB 0 Hz
GB 0
PC 1.00
3.9681 1.7205 1.8170 7.8153 2.0019 1.8059 1.9340 2.0000 5.8114
ppm

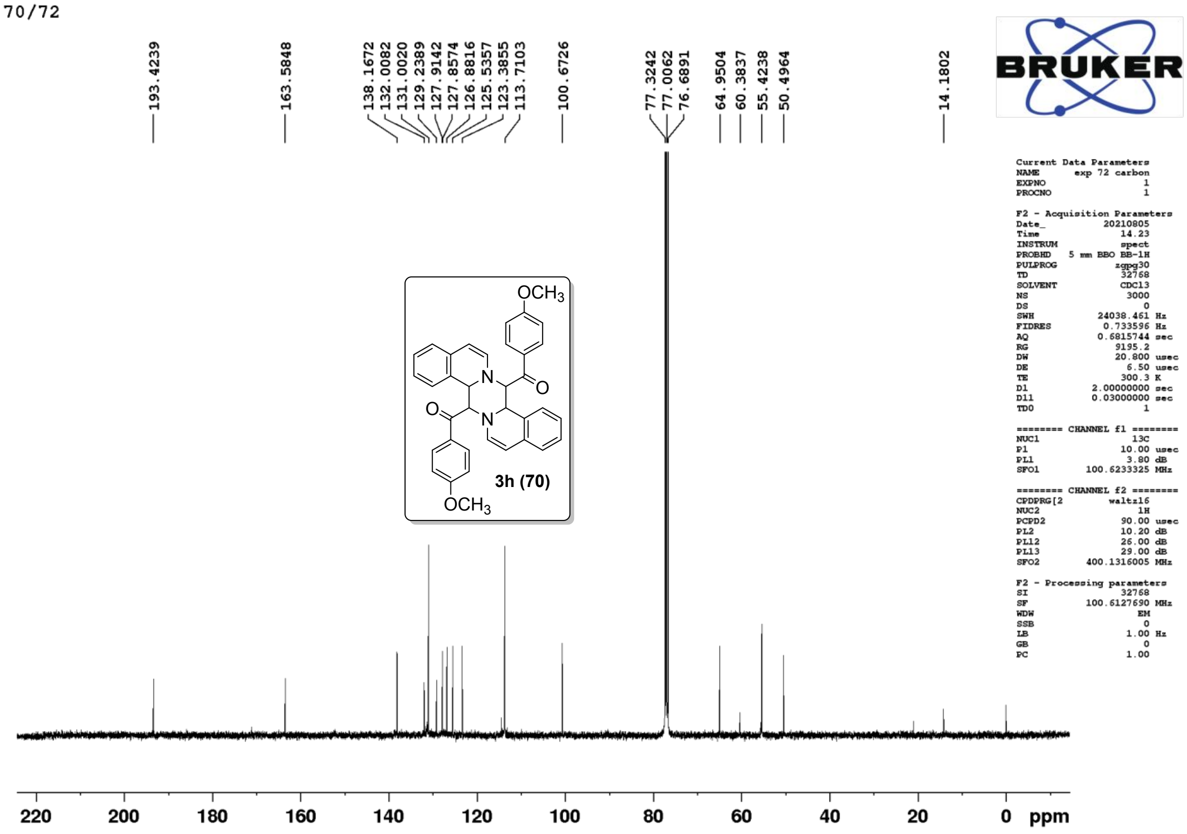
70/72
193.4239 163.5848 138.1672 132.0082 131.0020 129.2389 127.9142 127.8574 126.8816 125.5357 123.3855 113.7103 100.6726 77.3242 77.0062 76.6891 64.9504 60.3837 55.4238 50.4964 14.1802
Current Data Parameters
NAME exp 72 carbon
EXPNO 1
PROCNO 1
F2 - Acquisition Parameters
Date_ 20210805
Time 14.23
INSTRUM spect
PROBHD 5 mm BBO BB-1H
PULPROG zgpg30
TD 32768
SOLVENT CDCl3
NS 3000
DS 0
SWH 24038.461 Hz
FIDRES 0.733596 Hz
AQ 0.6815744 sec
RG 9195.2
DW 20.800 usec
DE 6.50 usec
TE 300.3 K
D1 2.00000000 sec
D11 0.03000000 sec
TD0 1
======== CHANNEL f1 ========
NUC1 13C
P1 10.00 usec
PL1 3.80 dB
SFO1 100.6233325 MHz
======== CHANNEL f2 ========
CPDPRG[2 waltz16
NUC2 1H
PCPD2 90.00 usec
PL2 10.20 dB
PL12 26.00 dB
PL13 29.00 dB
SFO2 400.1316005 MHz
F2 - Processing parameters
SI 32768
SF 100.6127690 MHz
WDW EM
SSB 0
LB 1.00 Hz
GB 0
PC 1.00
OCH3
N
O
3h (70)
ppm

# 3i (115)

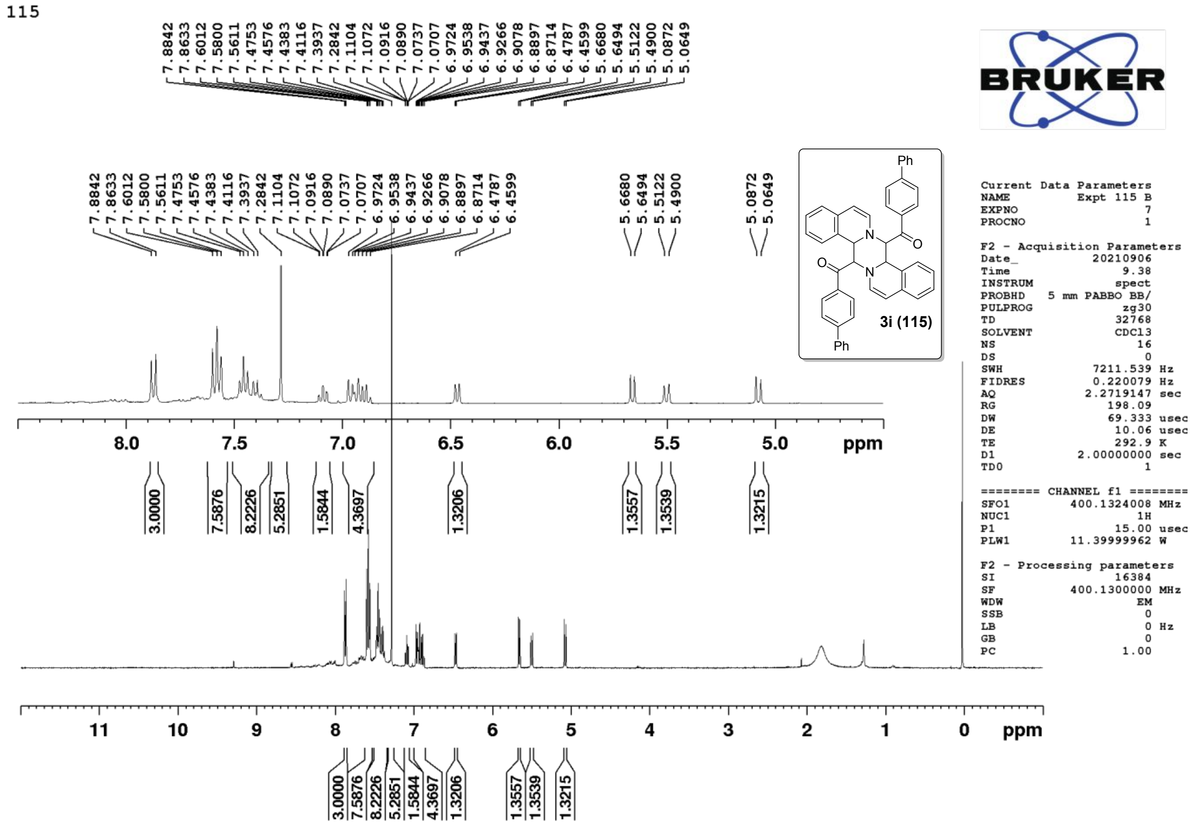
115
7.8842
7.8633
7.6012
7.5800
7.5611
7.4753
7.4576
7.4383
7.4116
7.3937
7.2842
7.1104
7.1072
7.0916
7.0890
7.0737
7.0707
6.9724
6.9538
6.9437
6.9266
6.9078
6.8897
6.8714
6.4787
6.4599
5.6680
5.6494
5.5122
5.4900
5.0872
5.0649
BRUKER
Ph
O
N
N
O
Ph
3i (115)
Current Data Parameters
NAME Expt 115 B
EXPNO 7
PROCNO 1
F2 - Acquisition Parameters
Date_ 20210906
Time 9.38
INSTRUM spect
PROBHD 5 mm PABBO BB/
PULPROG zg30
TD 32768
SOLVENT CDCl3
NS 16
DS 0
SWH 7211.539 Hz
FIDRES 0.220079 Hz
AQ 2.2719147 sec
RG 198.09
DW 69.333 usec
DE 10.06 usec
TE 292.9 K
D1 2.00000000 sec
TD0 1
======== CHANNEL f1 ========
SFO1 400.1324008 MHz
NUC1 1H
P1 15.00 usec
PLW1 11.39999962 W
F2 - Processing parameters
SI 16384
SF 400.1300000 MHz
WDW EM
SSB 0
LB 0 Hz
GB 0
PC 1.00
8.0 7.5 7.0 6.5 6.0 5.5 5.0 ppm
3.0000
7.5876
8.2226
5.2851
1.5844
4.3697
1.3206
1.3557
1.3539
1.3215
11 10 9 8 7 6 5 4 3 2 1 0 ppm

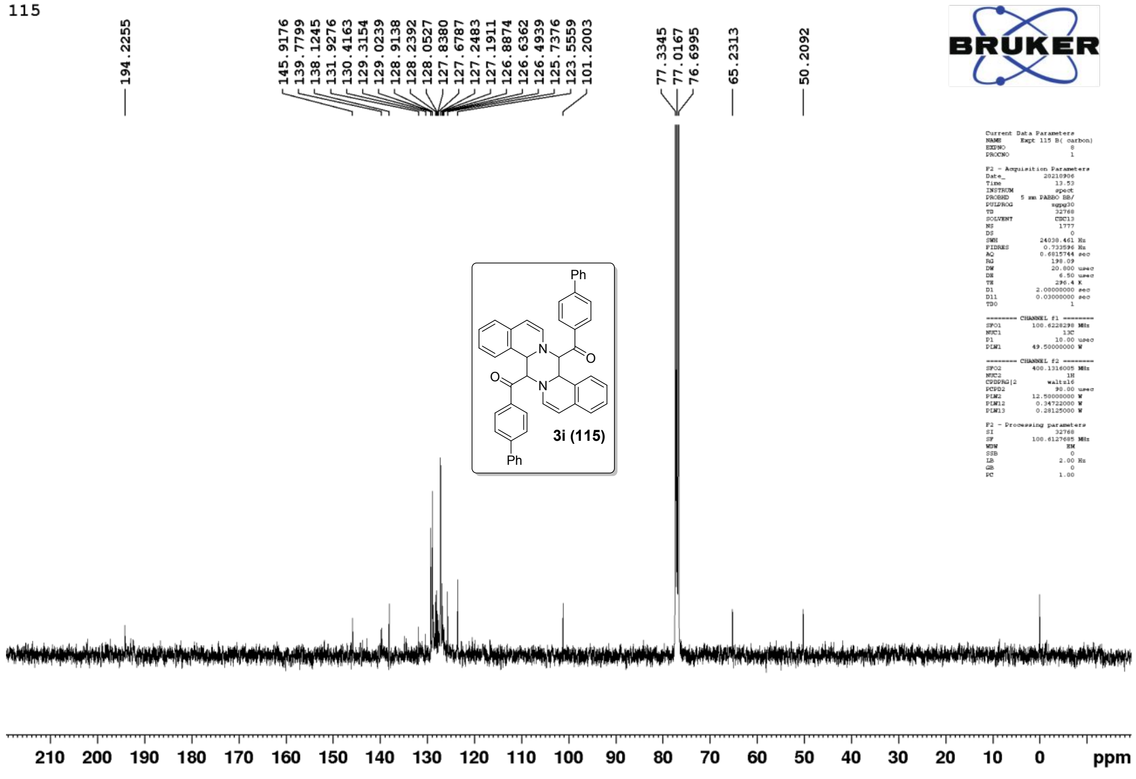
115
194.2255
145.9176
139.7799
138.1245
131.9276
130.4163
129.3154
129.0239
128.9138
128.2392
128.0527
127.8380
127.6787
127.2483
127.1911
126.8874
126.6362
126.4939
125.7376
123.5559
101.2003
77.3345
77.0167
76.6995
65.2313
50.2092
BRUKER
Ph
O
N
N
O
Ph
3i (115)
210 200 190 180 170 160 150 140 130 120 110 100 90 80 70 60 50 40 30 20 10 0 ppm

**3j (106)**

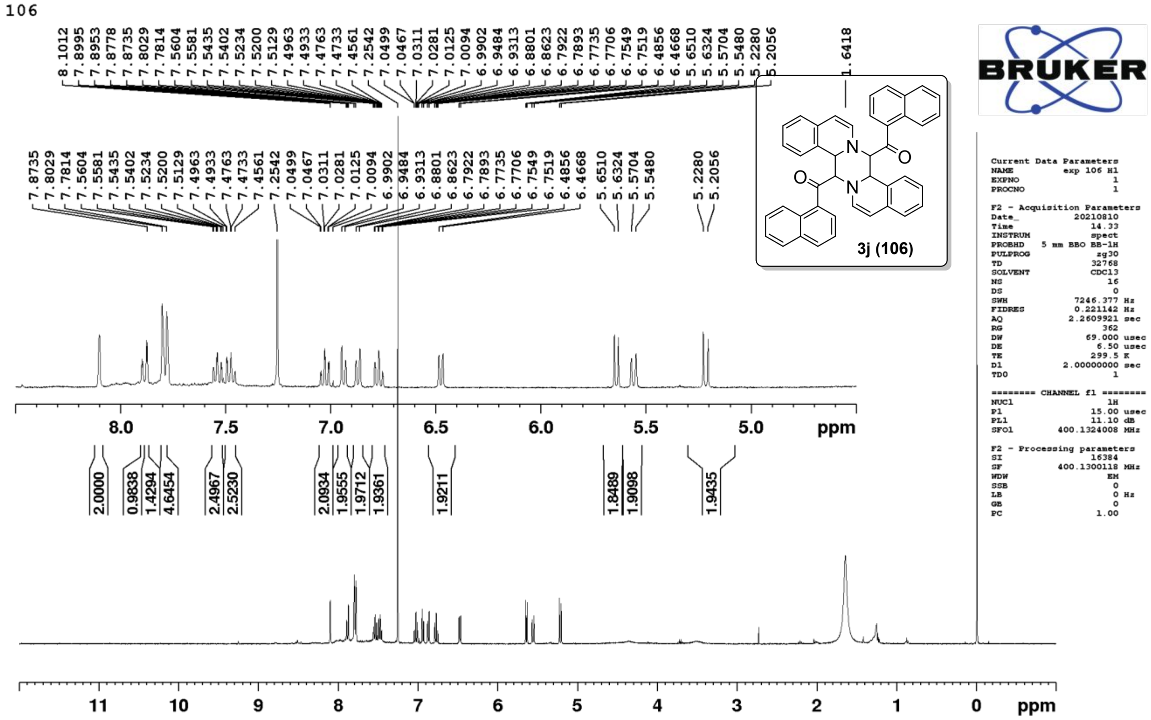

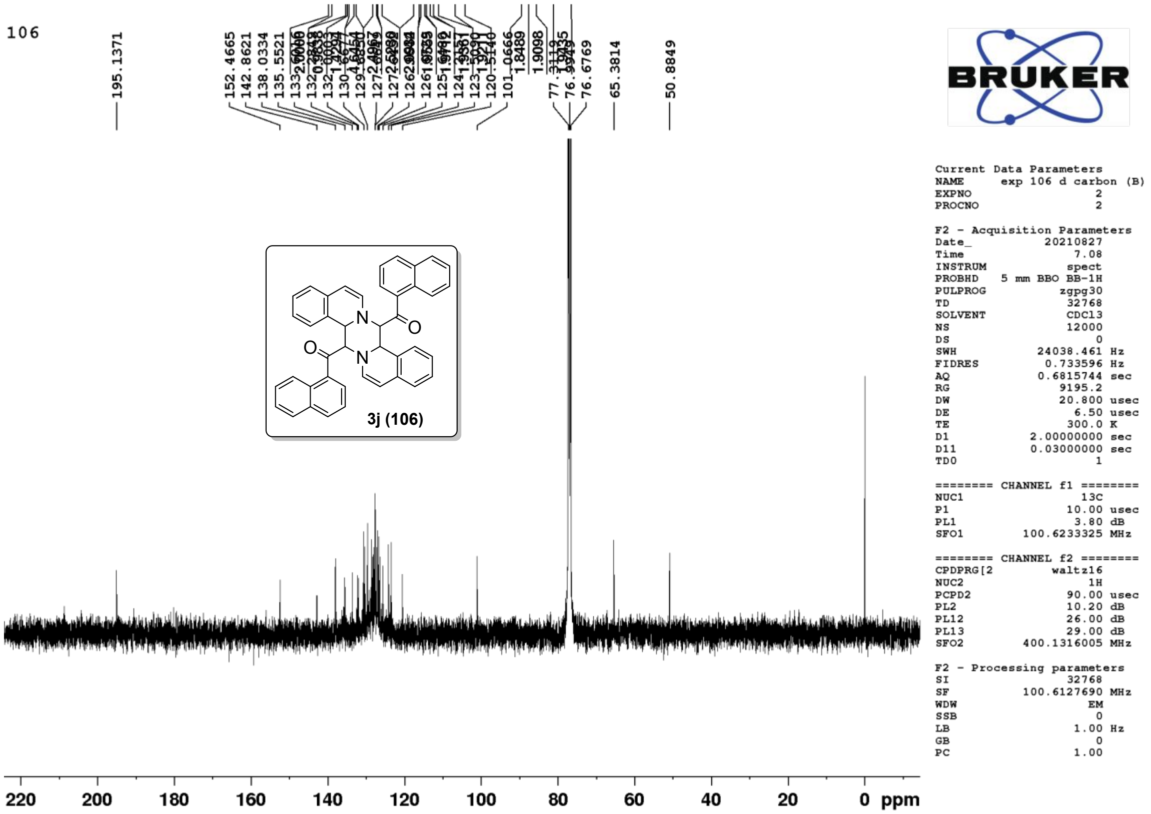

# 3k (111)

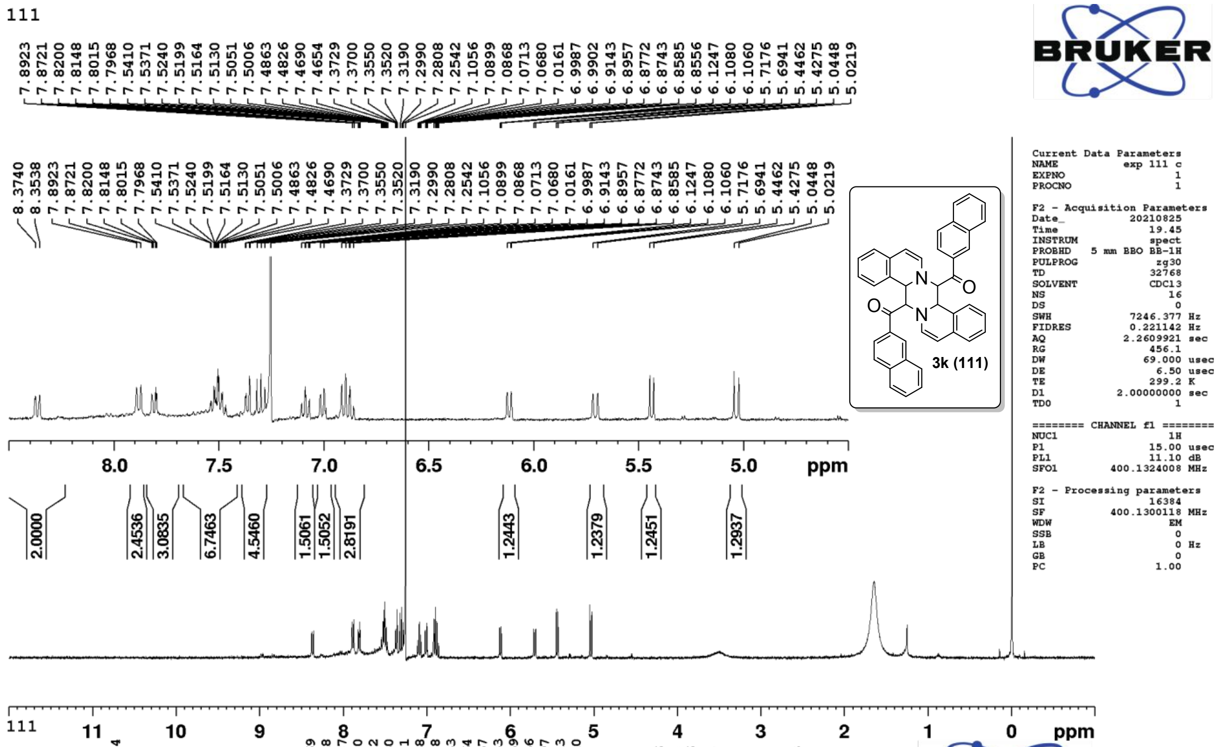
111
8.3740
8.3538
7.8923
7.8721
7.8200
7.8148
7.8015
7.7968
7.5410
7.5371
7.5240
7.5199
7.5164
7.5130
7.5051
7.5006
7.4863
7.4826
7.4690
7.4654
7.3729
7.3700
7.3550
7.3520
7.3190
7.2990
7.2808
7.2542
7.1056
7.0899
7.0868
7.0713
7.0680
7.0161
6.9987
6.9902
6.9143
6.8957
6.8772
6.8743
6.8585
6.8556
6.1247
6.1080
6.1060
5.7176
5.6941
5.4462
5.4275
5.0448
5.0219
3k (111)
8.0
7.5
7.0
6.5
6.0
5.5
5.0
ppm
2.0000
2.4536
3.0835
6.7463
4.5460
1.5061
1.5052
2.8191
1.2443
1.2379
1.2451
1.2937
111
11
10
9
8
7
6
5
4
3
2
1
0
ppm
Current Data Parameters
NAME exp 111 c
EXPNO 1
PROCNO 1
F2 - Acquisition Parameters
Date_ 20210825
Time 19.45
INSTRUM spect
PROBHD 5 mm BBO BB-1H
PULPROG zg30
TD 32768
SOLVENT CDCl3
NS 16
DS 0
SWH 7246.377 Hz
FIDRES 0.221142 Hz
AQ 2.2609921 sec
RG 456.1
DW 69.000 usec
DE 6.50 usec
TE 299.2 K
D1 2.00000000 sec
TD0 1
======== CHANNEL f1 ========
NUC1 1H
P1 15.00 usec
PL1 11.10 dB
SFO1 400.1324008 MHz
F2 - Processing parameters
SI 16384
SF 400.1300118 MHz
WDW EM
SSB 0
LB 0 Hz
GB 0
PC 1.00

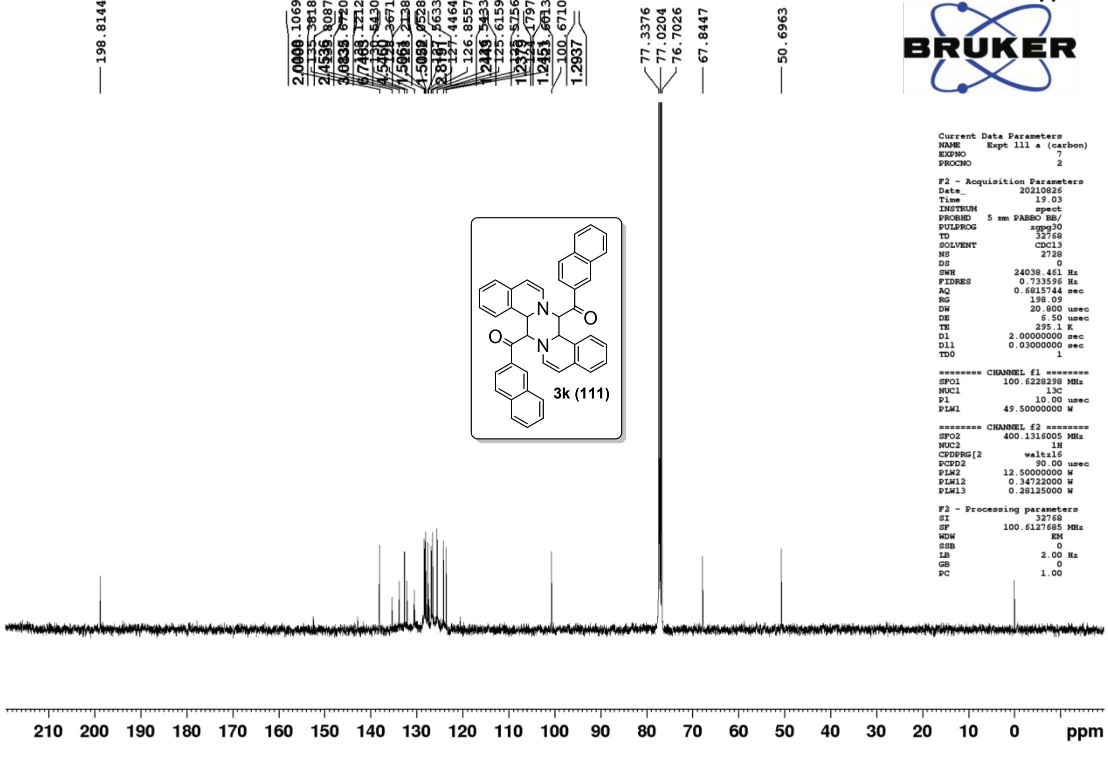
198.8144
77.3376
77.0204
76.7026
67.8447
50.6963
100.6710
3k (111)
Current Data Parameters
NAME Expt 111 a (carbon)
EXPNO 7
PROCNO 2
F2 - Acquisition Parameters
Date_ 20210826
Time 19.03
INSTRUM spect
PROBHD 5 mm PABBO BB/
PULPROG zgpg30
TD 32768
SOLVENT CDCl3
NS 2728
DS 0
SWH 24038.461 Hz
FIDRES 0.733596 Hz
AQ 0.6815744 sec
RG 198.09
DW 20.800 usec
DE 6.50 usec
TE 295.1 K
D1 2.00000000 sec
D11 0.03000000 sec
TD0 1
======== CHANNEL f1 ========
SFO1 100.6228298 MHz
NUC1 13C
P1 10.00 usec
PLW1 49.50000000 W
======== CHANNEL f2 ========
SFO2 400.1316005 MHz
NUC2 1H
CPDPRG[2 waltz16
PCPD2 90.00 usec
PLW2 12.50000000 W
PLW12 0.34722000 W
PLW13 0.28125000 W
F2 - Processing parameters
SI 32768
SF 100.6127685 MHz
WDW EM
SSB 0
LB 2.00 Hz
GB 0
PC 1.00
210
200
190
180
170
160
150
140
130
120
110
100
90
80
70
60
50
40
30
20
10
0
ppm

**3l (88)**

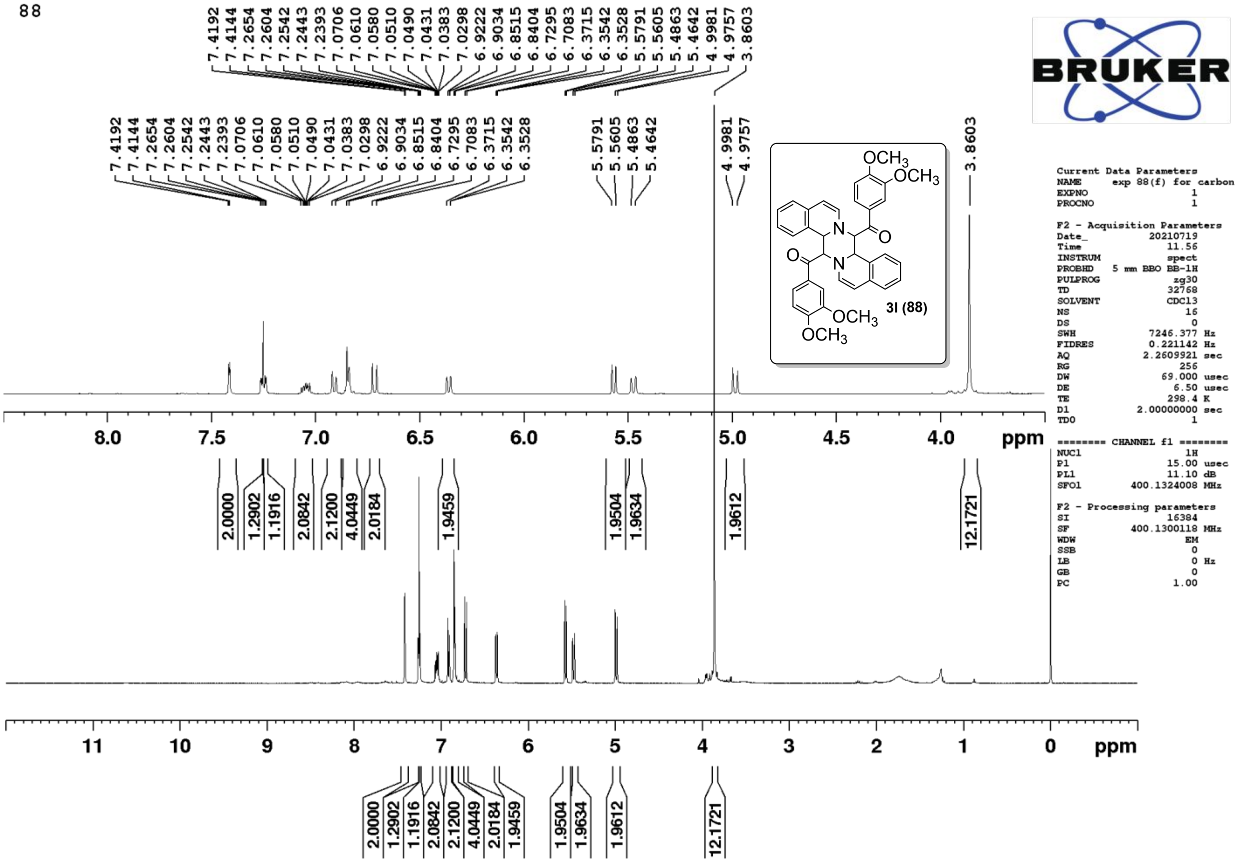
88
7.4192 7.4144 7.2654 7.2604 7.2542 7.2443 7.2393 7.0706 7.0610 7.0580 7.0510 7.0490 7.0431 7.0383 7.0298 6.9222 6.9034 6.8515 6.8404 6.7295 6.7083 6.3715 6.3542 6.3528 5.5791 5.5605 5.4863 5.4642 4.9981 4.9757 3.8603
BRUKER
OCH3
OCH3
N
O
N
O
OCH3
OCH3
3l (88)
Current Data Parameters
NAME exp 88(f) for carbon
EXPNO 1
PROCNO 1
F2 - Acquisition Parameters
Date_ 20210719
Time 11.56
INSTRUM spect
PROBHD 5 mm BBO BB-1H
PULPROG zg30
TD 32768
SOLVENT CDCl3
NS 16
DS 0
SWH 7246.377 Hz
FIDRES 0.221142 Hz
AQ 2.2609921 sec
RG 256
DW 69.000 usec
DE 6.50 usec
TE 298.4 K
D1 2.00000000 sec
TD0 1
======== CHANNEL f1 ========
NUC1 1H
P1 15.00 usec
PL1 11.10 dB
SFO1 400.1324008 MHz
F2 - Processing parameters
SI 16384
SF 400.1300118 MHz
WDW EM
SSB 0
LB 0 Hz
GB 0
PC 1.00
8.0 7.5 7.0 6.5 6.0 5.5 5.0 4.5 4.0 ppm
2.0000 1.2902 1.1916 2.0842 2.1200 4.0449 2.0184 1.9459 1.9504 1.9634 1.9612 12.1721
11 10 9 8 7 6 5 4 3 2 1 0 ppm

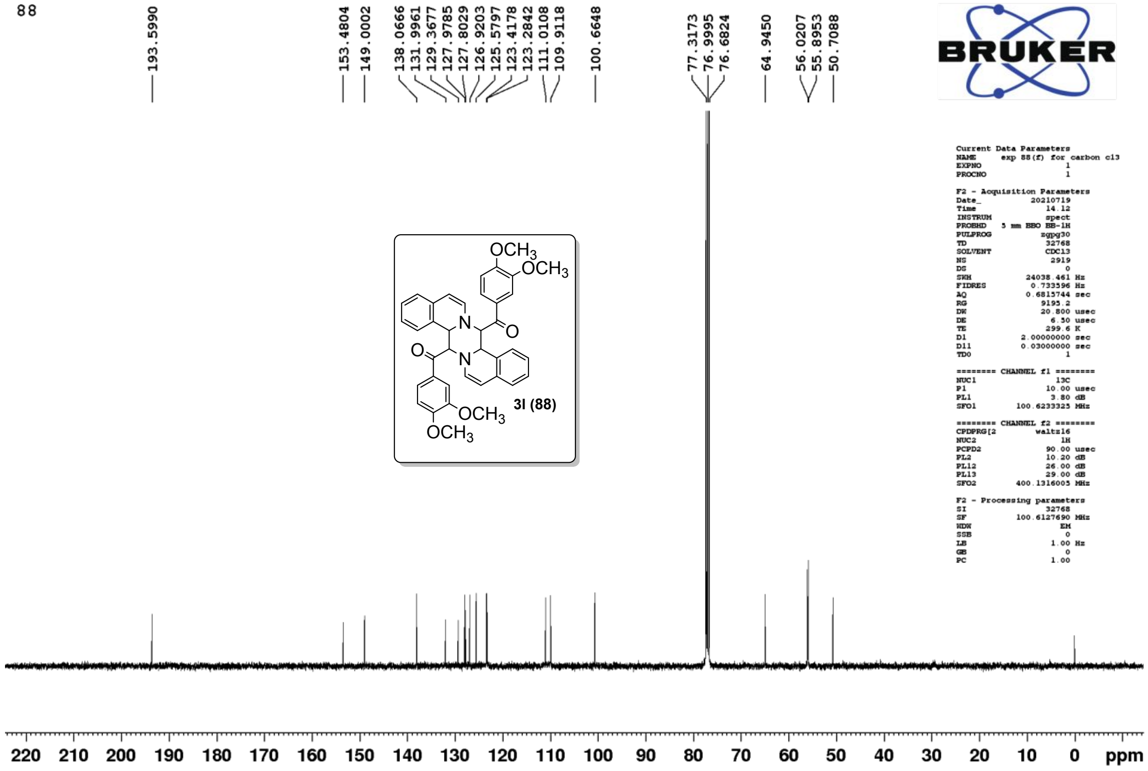
88
193.5990 153.4804 149.0002 138.0666 131.9961 129.3677 127.9785 127.8029 126.9203 125.5797 123.4178 123.2842 111.0108 109.9118 100.6648 77.3173 76.9995 76.6824 64.9450 56.0207 55.8953 50.7088
BRUKER
Current Data Parameters
NAME exp 88(f) for carbon c13
EXPNO 1
PROCNO 1
F2 - Acquisition Parameters
Date_ 20210719
Time 14.12
INSTRUM spect
PROBHD 5 mm BBO BB-1H
PULPROG zgpg30
TD 32768
SOLVENT CDCl3
NS 2919
DS 0
SWH 24038.461 Hz
FIDRES 0.733596 Hz
AQ 0.6815744 sec
RG 9195.2
DW 20.800 usec
DE 6.50 usec
TE 299.6 K
D1 2.00000000 sec
D11 0.03000000 sec
TD0 1
======== CHANNEL f1 ========
NUC1 13C
P1 10.00 usec
PL1 3.80 dB
SFO1 100.6233325 MHz
======== CHANNEL f2 ========
CPDPRG[2 waltz16
NUC2 1H
PCPD2 90.00 usec
PL2 10.20 dB
PL12 26.00 dB
PL13 29.00 dB
SFO2 400.1316005 MHz
F2 - Processing parameters
SI 32768
SF 100.6127690 MHz
WDW EM
SSB 0
LB 1.00 Hz
GB 0
PC 1.00
OCH3
OCH3
N
O
N
O
OCH3
OCH3
3l (88)
220 210 200 190 180 170 160 150 140 130 120 110 100 90 80 70 60 50 40 30 20 10 0 ppm

**3m (98)**

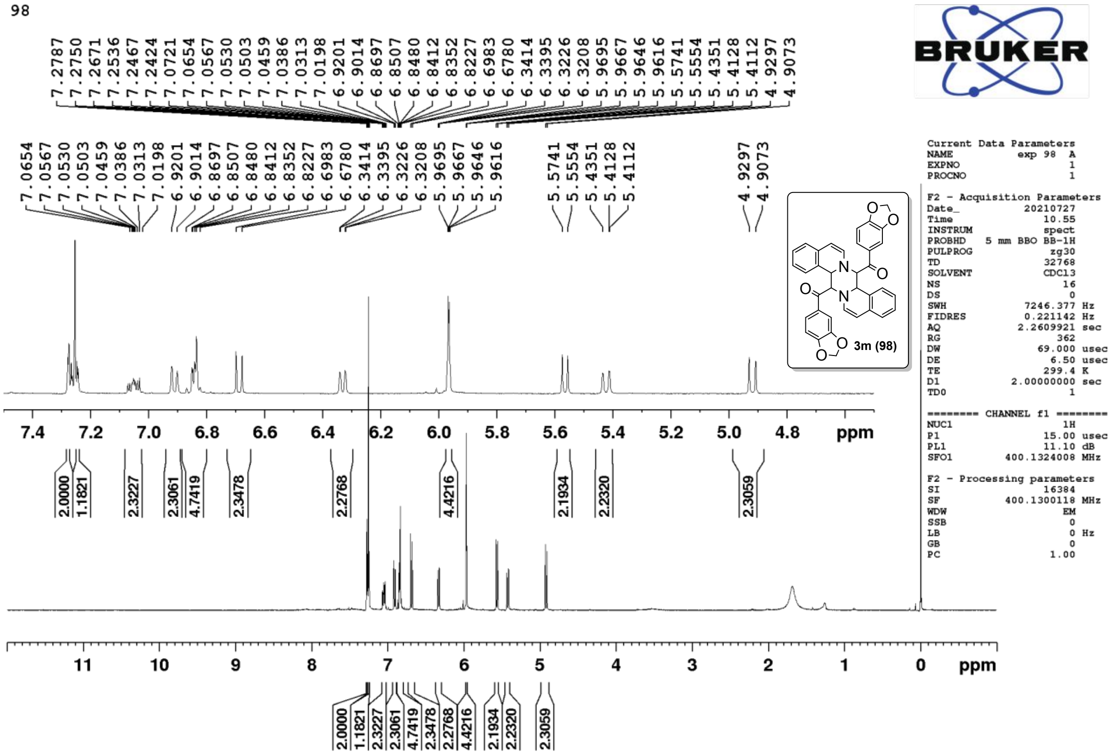
98
7.2787 7.2750 7.2671 7.2536 7.2467 7.2424 7.0721 7.0654 7.0567 7.0530 7.0503 7.0459 7.0386 7.0313 7.0198 6.9201 6.9014 6.8697 6.8507 6.8480 6.8412 6.8352 6.8227 6.6983 6.6780 6.3414 6.3395 6.3226 6.3208 5.9695 5.9667 5.9646 5.9616 5.5741 5.5554 5.4351 5.4128 5.4112 4.9297 4.9073
BRUKER
Current Data Parameters
NAME exp 98 A
EXPNO 1
PROCNO 1
F2 - Acquisition Parameters
Date_ 20210727
Time 10.55
INSTRUM spect
PROBHD 5 mm BBO BB-1H
PULPROG zg30
TD 32768
SOLVENT CDCl3
NS 16
DS 0
SWH 7246.377 Hz
FIDRES 0.221142 Hz
AQ 2.2609921 sec
RG 362
DW 69.000 usec
DE 6.50 usec
TE 299.4 K
D1 2.00000000 sec
TD0 1
======== CHANNEL f1 ========
NUC1 1H
P1 15.00 usec
PL1 11.10 dB
SFO1 400.1324008 MHz
F2 - Processing parameters
SI 16384
SF 400.1300118 MHz
WDW EM
SSB 0
LB 0 Hz
GB 0
PC 1.00
3m (98)
2.0000 1.1821 2.3227 2.3061 4.7419 2.3478 2.2768 4.4216 2.1934 2.2320 2.3059
ppm

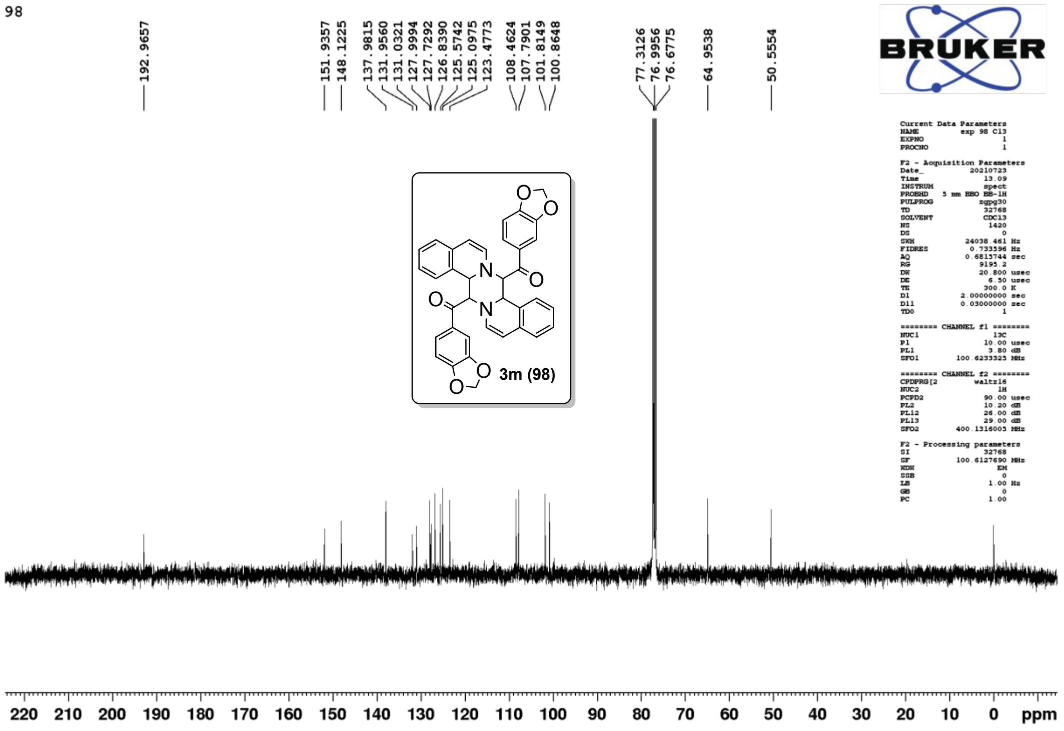
98
192.9657 151.9357 148.1225 137.9815 131.9560 131.0321 127.9994 127.7292 126.8390 125.5742 125.0975 123.4773 108.4624 107.7901 101.8149 100.8648 77.3126 76.9956 76.6775 64.9538 50.5554
BRUKER
3m (98)
ppm

## 3n (114)

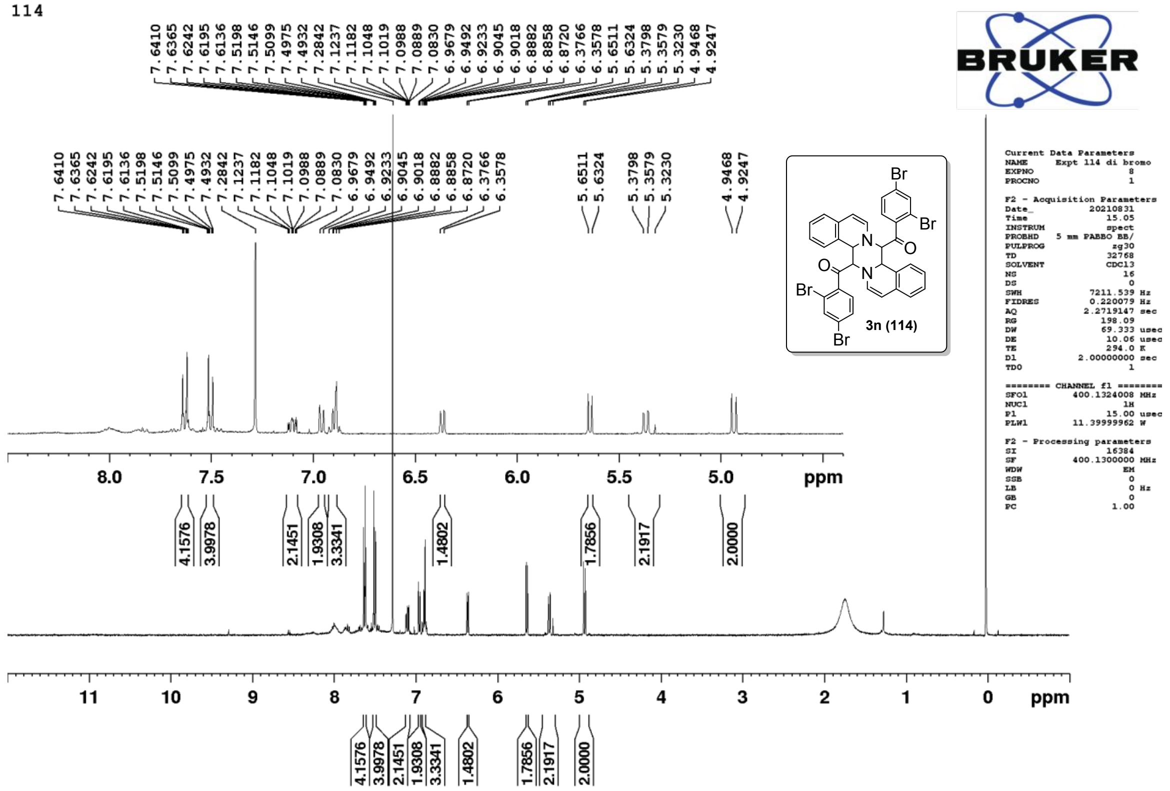
114
7.6410 7.6365 7.6242 7.6195 7.6136 7.5198 7.5146 7.5099 7.4975 7.4932 7.2842 7.1237 7.1182 7.1048 7.1019 7.0988 7.0889 7.0830 6.9679 6.9492 6.9233 6.9045 6.9018 6.8882 6.8858 6.8720 6.3766 6.3578 5.6511 5.6324 5.3798 5.3579 5.3230 4.9468 4.9247
BRUKER
Br
Br
O
N
N
O
Br
Br
3n (114)
8.0 7.5 7.0 6.5 6.0 5.5 5.0 ppm
4.1576 3.9978 2.1451 1.9308 3.3341 1.4802 1.7856 2.1917 2.0000
11 10 9 8 7 6 5 4 3 2 1 0 ppm

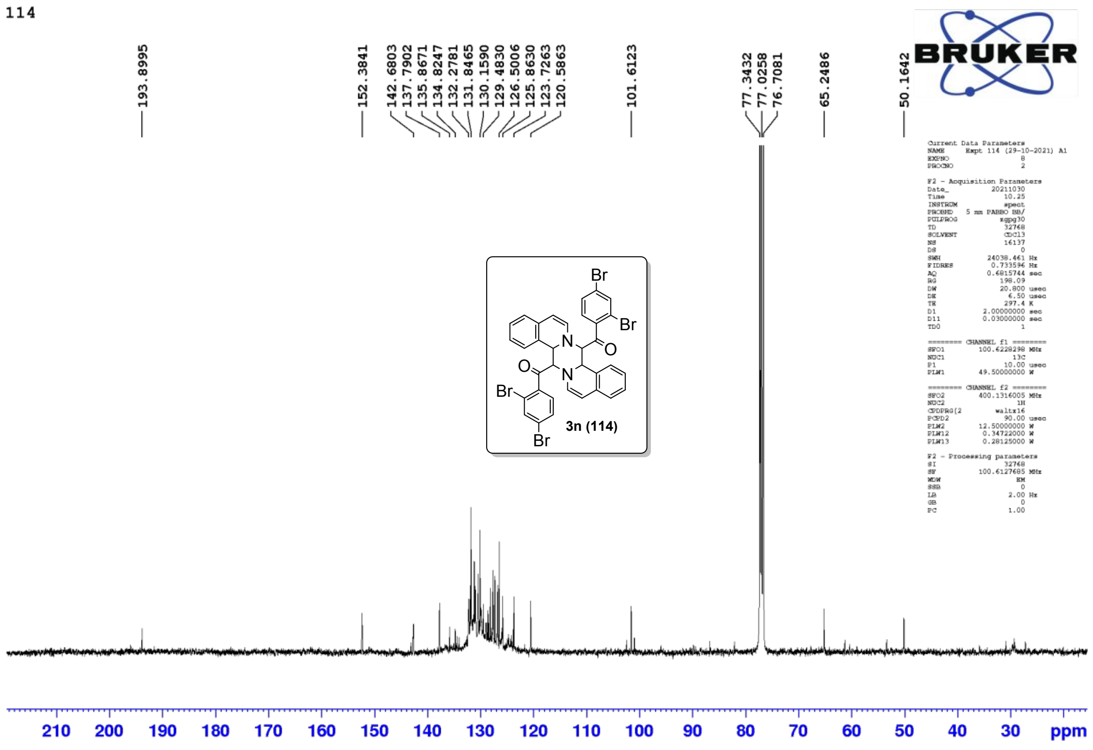
114
193.8995
152.3841 142.6803 137.7902 135.8671 134.8247 132.2781 131.8465 130.1590 129.4830 126.5006 125.8630 123.7263 120.5863
101.6123
77.3432 77.0258 76.7081
65.2486
50.1642
BRUKER
Br
Br
O
N
N
O
Br
Br
3n (114)
210 200 190 180 170 160 150 140 130 120 110 100 90 80 70 60 50 40 30 ppm

# 3o (122)

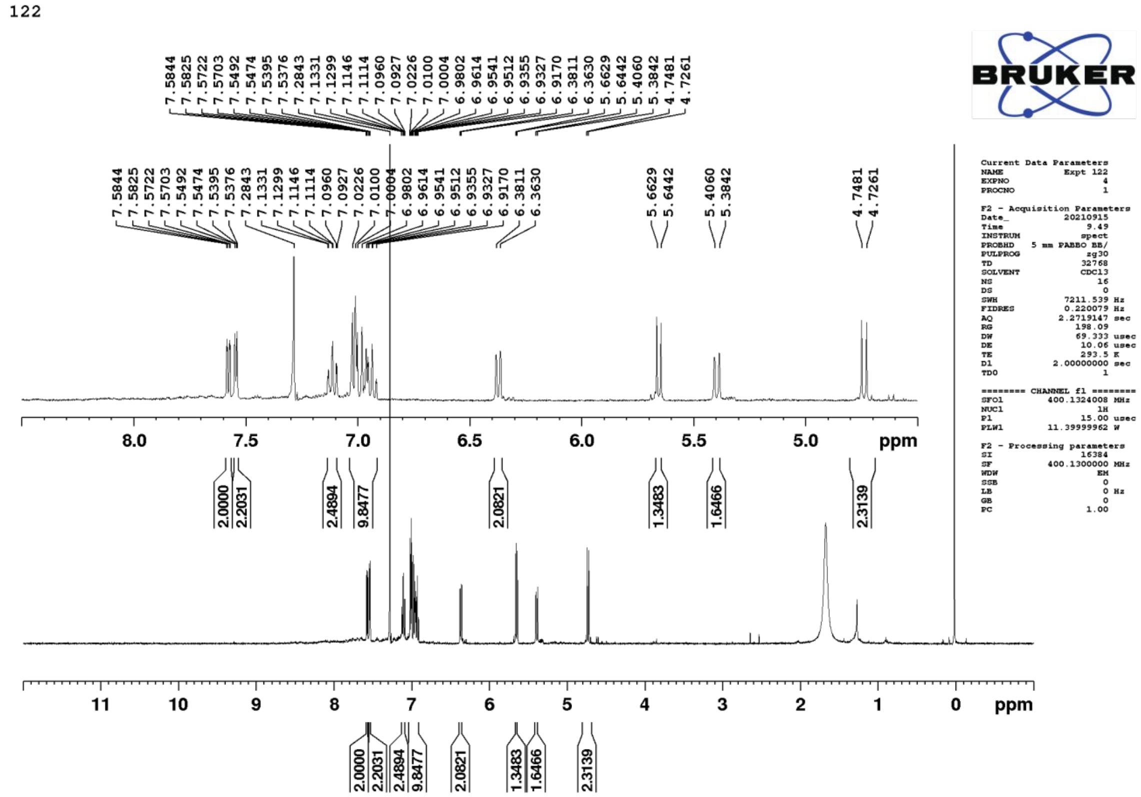

122
7.5844 7.5825 7.5722 7.5703 7.5492 7.5474 7.5395 7.5376 7.2843 7.1331 7.1299 7.1146 7.1114 7.0960 7.0927 7.0226 7.0100 7.0004 6.9802 6.9614 6.9541 6.9512 6.9355 6.9327 6.9170 6.3811 6.3630 5.6629 5.6442 5.4060 5.3842 4.7481 4.7261
2.0000 2.2031 2.4894 9.8477 2.0821 1.3483 1.6466 2.3139
ppm


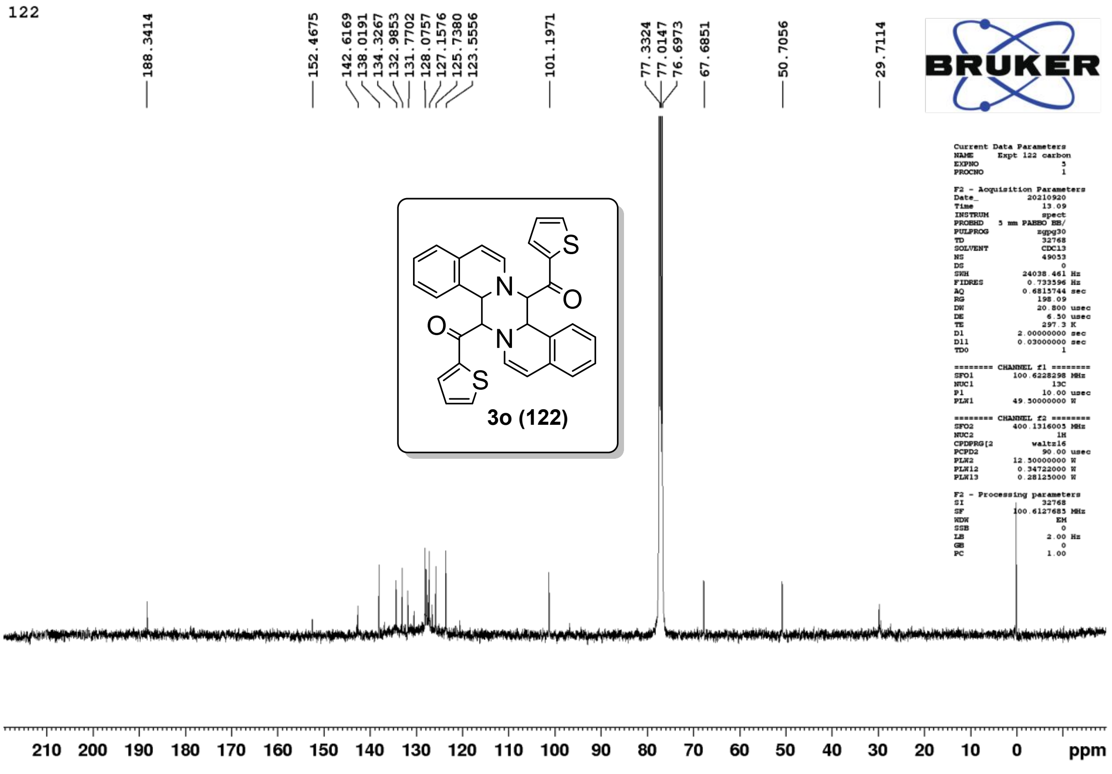

122
188.3414 152.4675 142.6169 138.0191 134.3267 132.9853 131.7702 128.0757 127.1576 125.7380 123.5556 101.1971 77.3324 77.0147 76.6973 67.6851 50.7056 29.7114
3o (122)
ppm

**3p (50Br)**

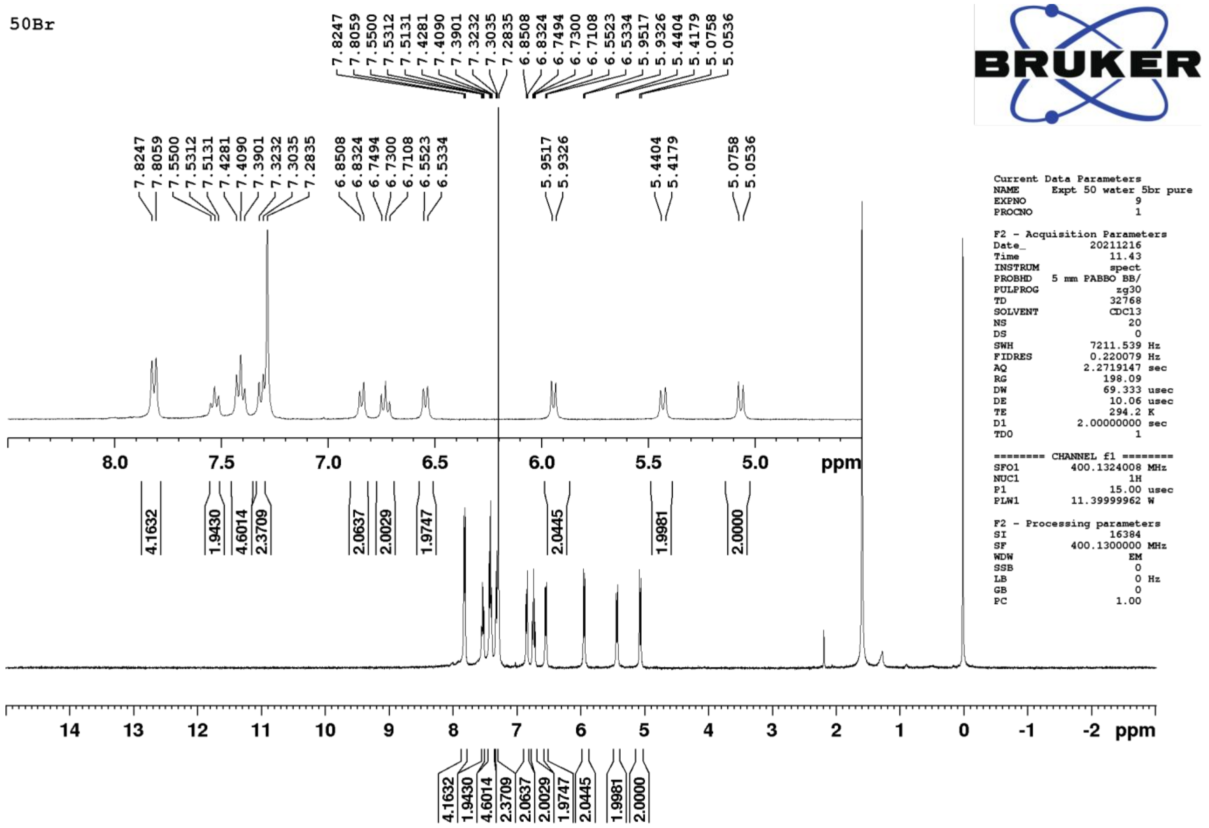

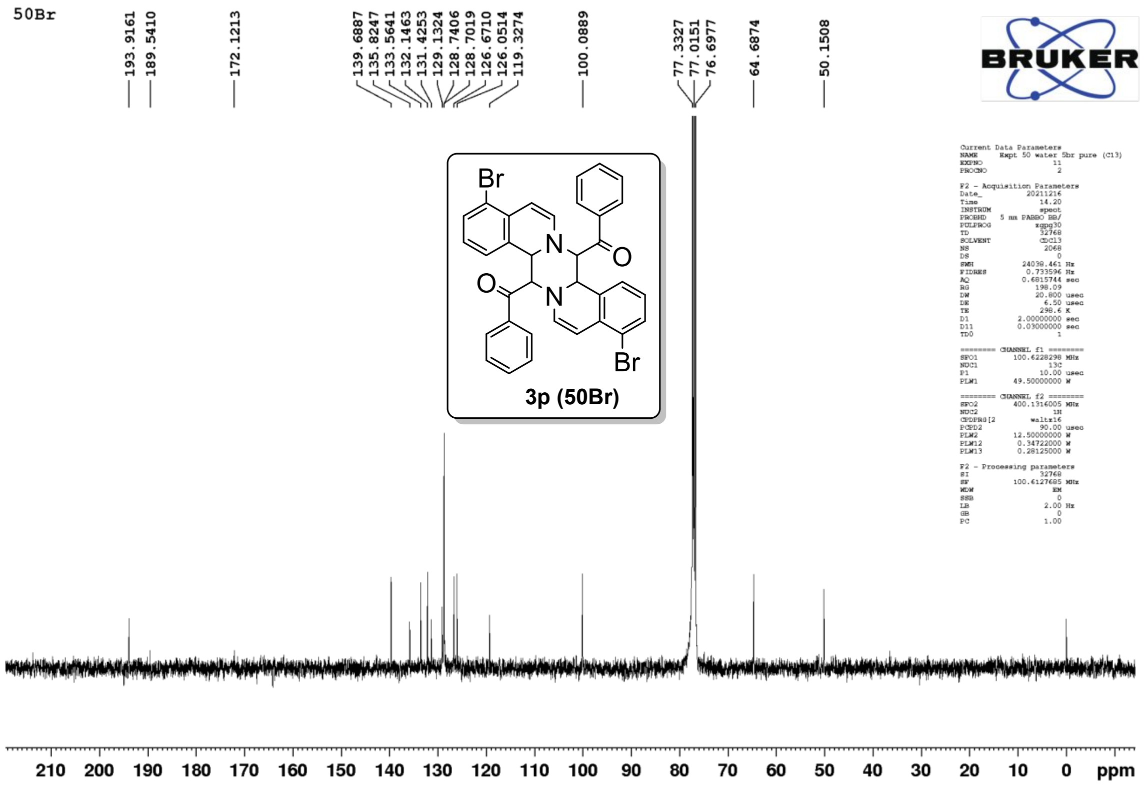

## 3q (103Br)

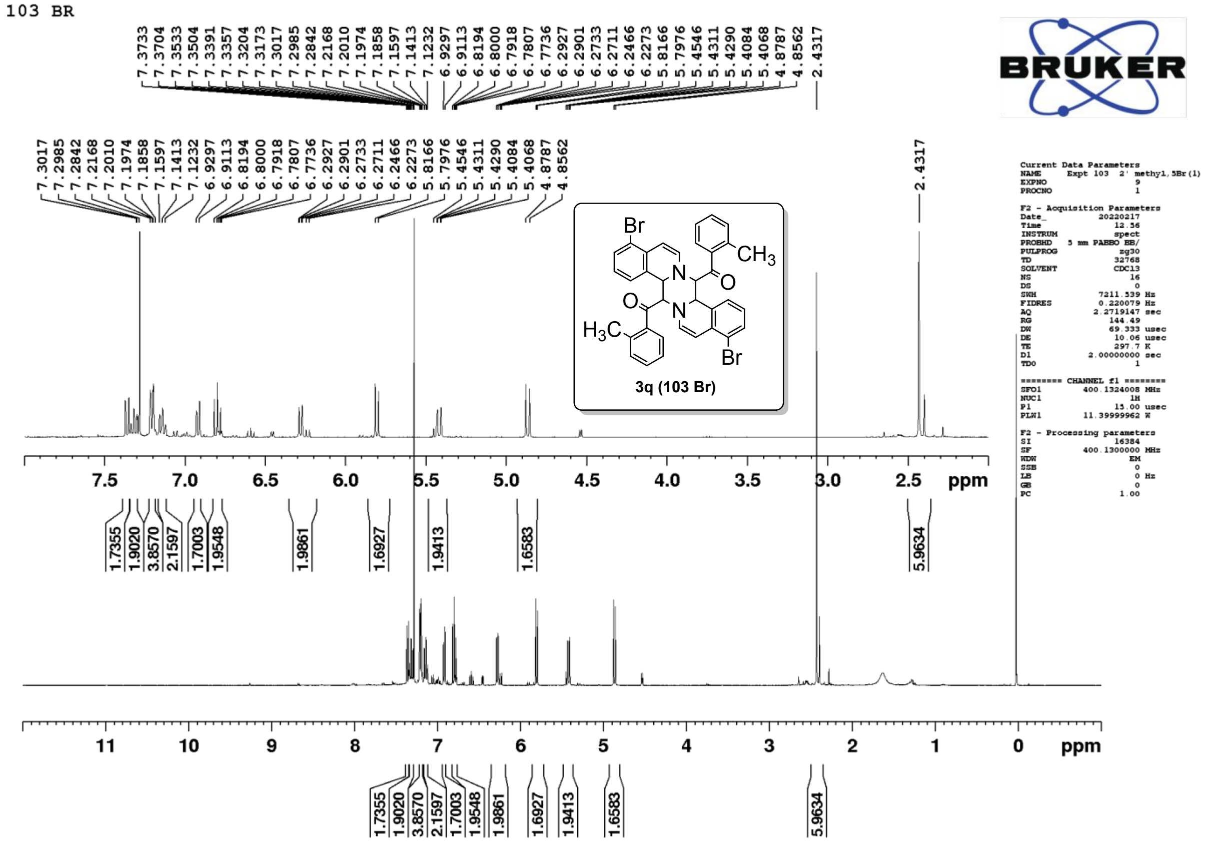
103 BR
7.3733 7.3704 7.3533 7.3504 7.3391 7.3357 7.3204 7.3173 7.3017 7.2985 7.2842 7.2168 7.2010 7.1974 7.1858 7.1597 7.1413 7.1232 6.9297 6.9113 6.8194 6.8000 6.7918 6.7807 6.7736 6.2927 6.2901 6.2733 6.2711 6.2466 6.2273 5.8166 5.7976 5.4546 5.4311 5.4290 5.4084 5.4068 4.8787 4.8562 2.4317
3q (103 Br)
ppm
1.7355 1.9020 3.8570 2.1597 1.7003 1.9548 1.9861 1.6927 1.9413 1.6583 5.9634

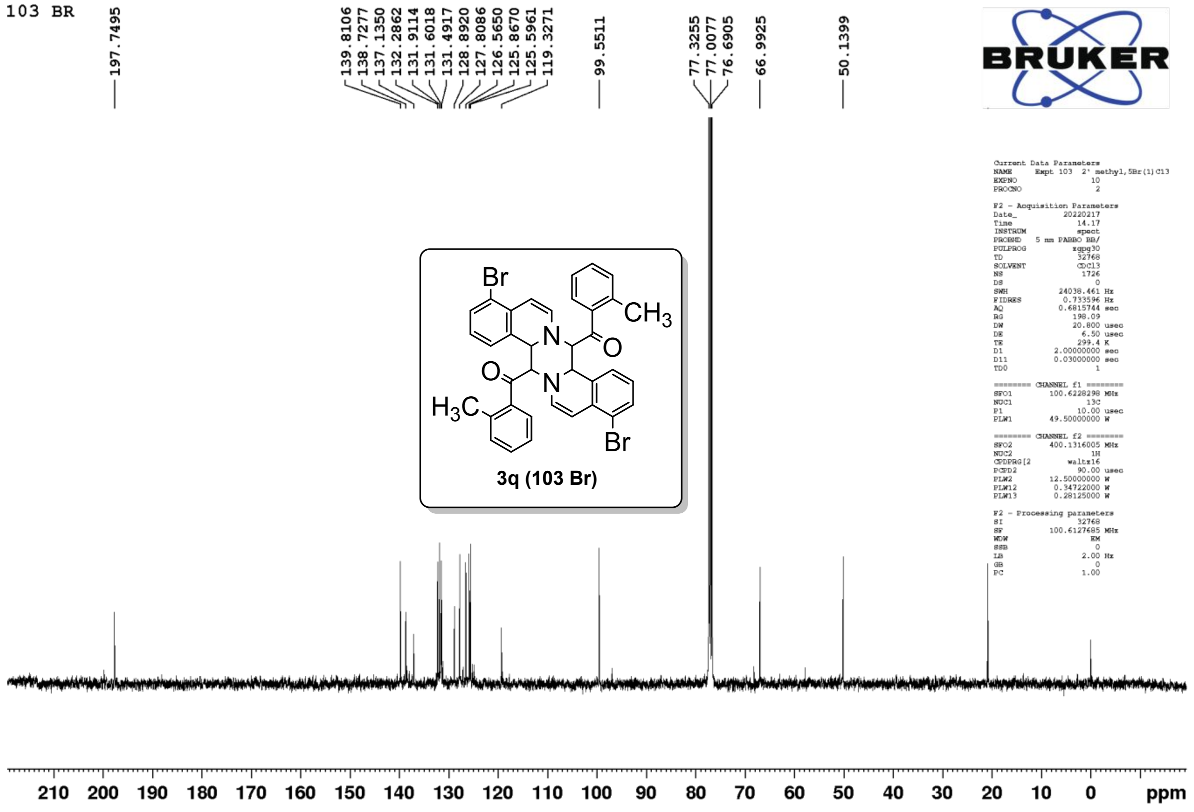
103 BR
197.7495
139.8106 138.7277 137.1350 132.2862 131.9114 131.6018 131.4917 128.8920 127.8086 126.5650 125.8670 125.5961 119.3271
99.5511
77.3255 77.0077 76.6905
66.9925
50.1399
3q (103 Br)
ppm

**3r (91Br)**

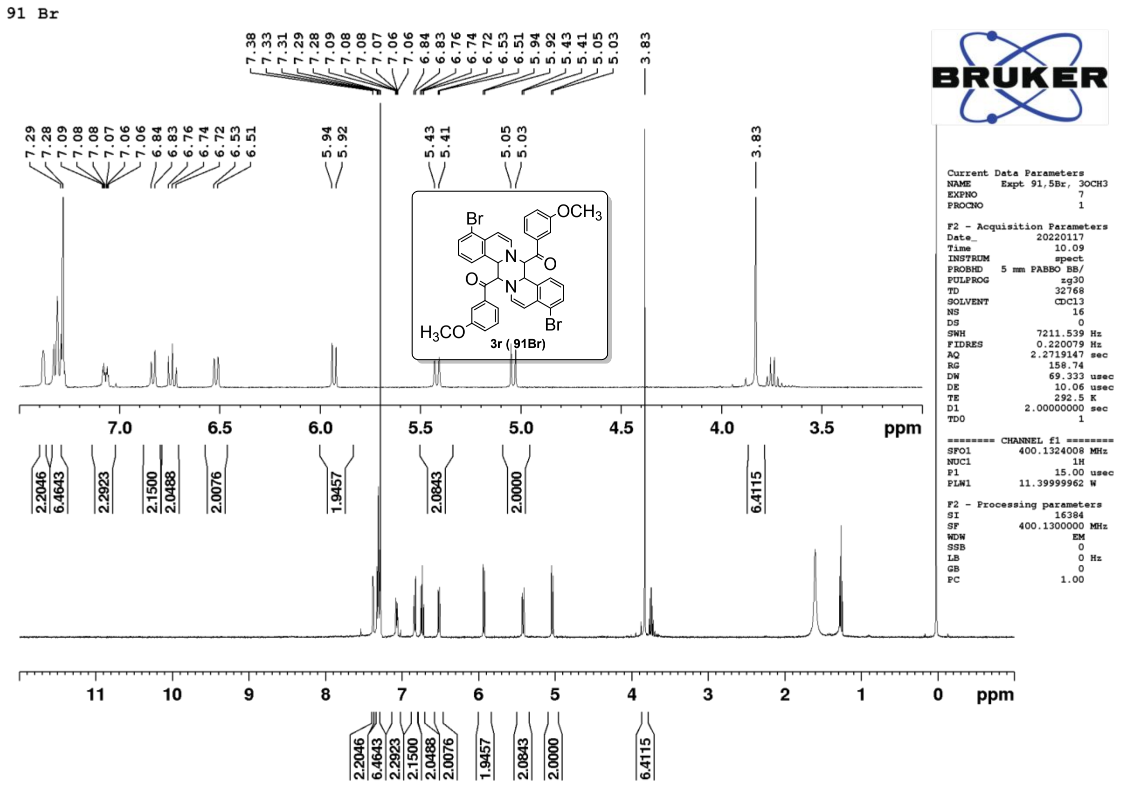
91 Br
7.38 7.33 7.31 7.29 7.28 7.09 7.08 7.08 7.07 7.06 7.06 6.84 6.83 6.76 6.74 6.72 6.53 6.51 5.94 5.92 5.43 5.41 5.05 5.03 3.83
BRUKER
3r ( 91Br)
ppm

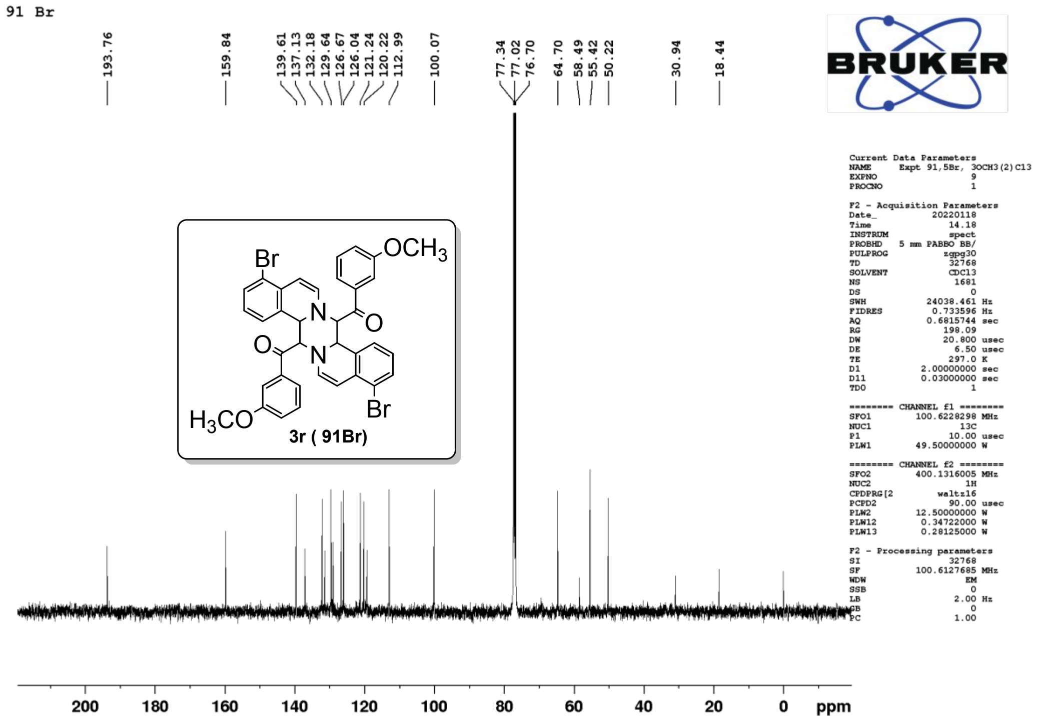
91 Br
193.76 159.84 139.61 137.13 132.18 129.64 126.67 126.04 121.24 120.22 112.99 100.07 77.34 77.02 76.70 64.70 58.49 55.42 50.22 30.94 18.44
BRUKER
3r ( 91Br)
200 180 160 140 120 100 80 60 40 20 0 ppm

## 3s (68Br)

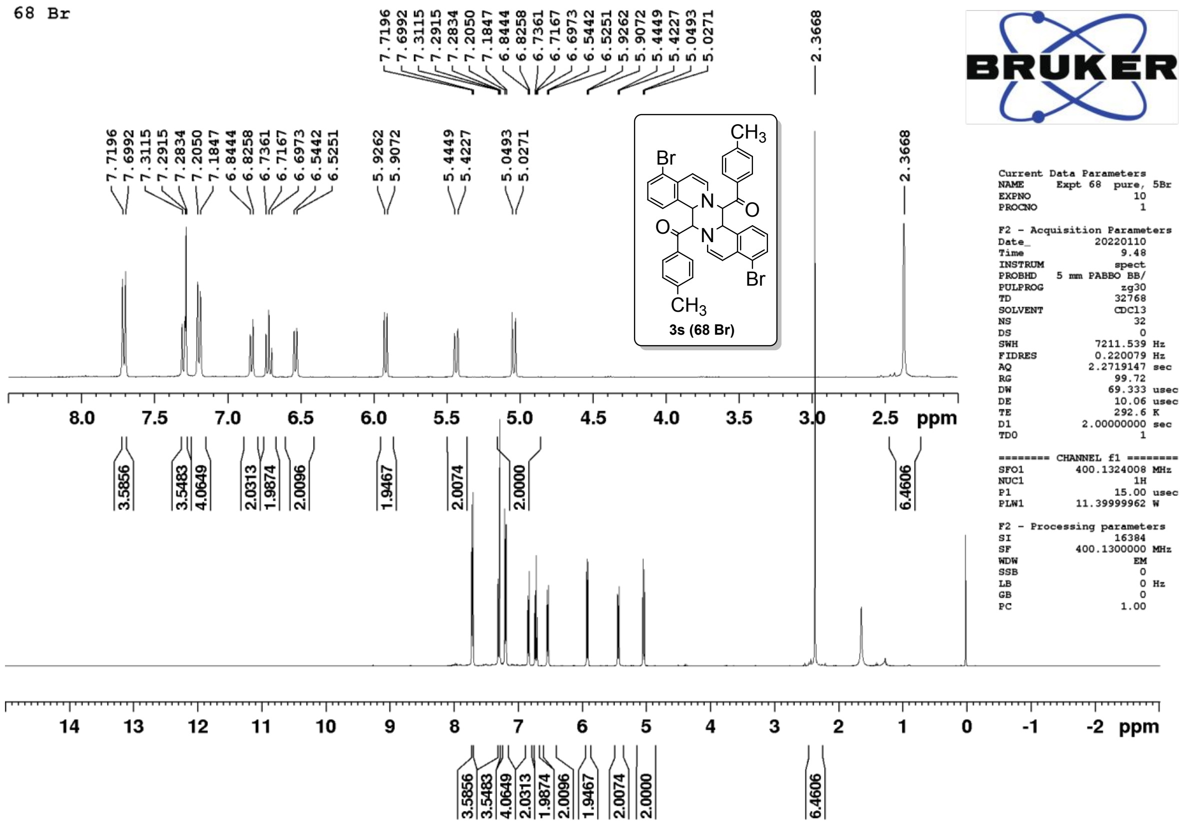
68 Br
7.7196
7.6992
7.3115
7.2915
7.2834
7.2050
7.1847
6.8444
6.8258
6.7361
6.7167
6.6973
6.5442
6.5251
5.9262
5.9072
5.4449
5.4227
5.0493
5.0271
2.3668
BRUKER
Br
N
O
CH3
3s (68 Br)
3.5856
3.5483
4.0649
2.0313
1.9874
2.0096
1.9467
2.0074
2.0000
6.4606
ppm
Current Data Parameters
NAME Expt 68 pure, 5Br
EXPNO 10
PROCNO 1
F2 - Acquisition Parameters
Date_ 20220110
Time 9.48
INSTRUM spect
PROBHD 5 mm PABBO BB/
PULPROG zg30
TD 32768
SOLVENT CDCl3
NS 32
DS 0
SWH 7211.539 Hz
FIDRES 0.220079 Hz
AQ 2.2719147 sec
RG 99.72
DW 69.333 usec
DE 10.06 usec
TE 292.6 K
D1 2.00000000 sec
TD0 1
======== CHANNEL f1 ========
SFO1 400.1324008 MHz
NUC1 1H
P1 15.00 usec
PLW1 11.39999962 W
F2 - Processing parameters
SI 16384
SF 400.1300000 MHz
WDW EM
SSB 0
LB 0 Hz
GB 0
PC 1.00

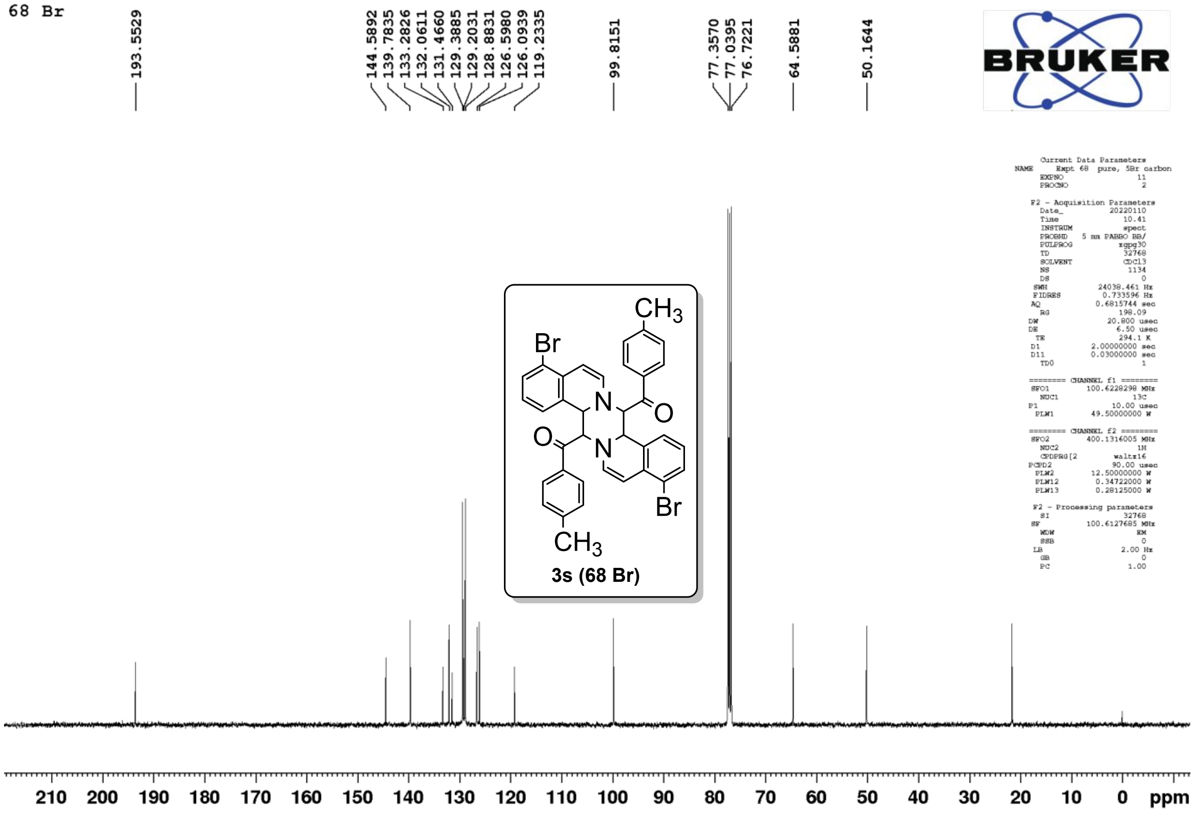
68 Br
193.5529
144.5892
139.7835
133.2826
132.0611
131.4660
129.3885
129.2031
128.8831
126.5980
126.0939
119.2335
99.8151
77.3570
77.0395
76.7221
64.5881
50.1644
BRUKER
Br
N
O
CH3
3s (68 Br)
ppm

**3t (70Br)**

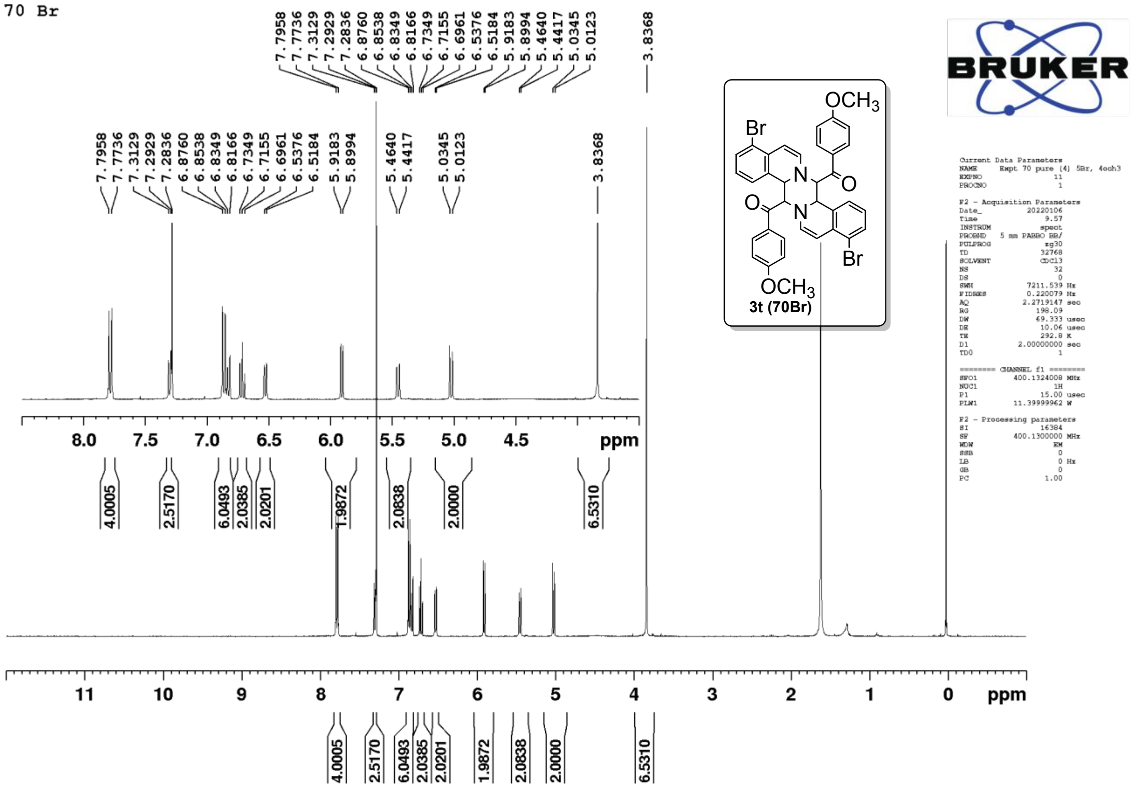
70 Br
7.7958 7.7736 7.3129 7.2929 7.2836 6.8760 6.8538 6.8349 6.8166 6.7349 6.7155 6.6961 6.5376 6.5184 5.9183 5.8994 5.4640 5.4417 5.0345 5.0123
3.8368
4.0005 2.5170 6.0493 2.0385 2.0201 1.9872 2.0838 2.0000 6.5310
3t (70Br)
ppm

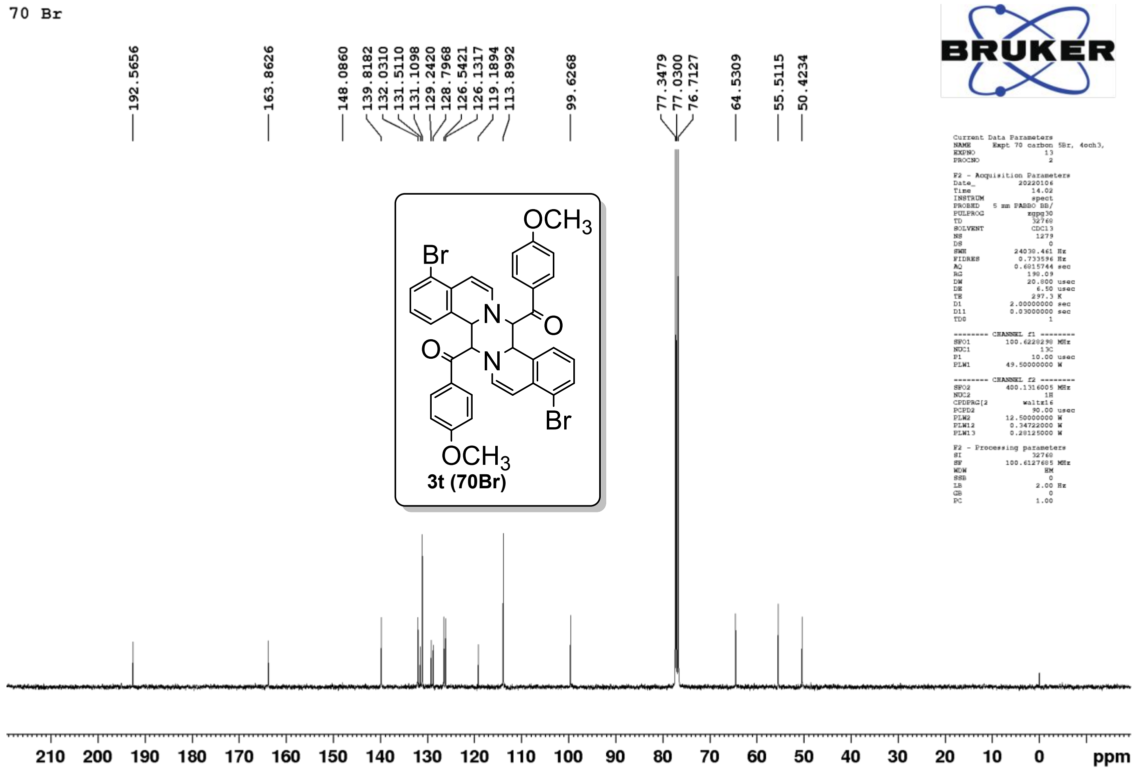
70 Br
192.5656
163.8626
148.0860 139.8182 132.0310 131.5110 131.1098 129.2420 128.7968 126.5421 126.1317 119.1894 113.8992
99.6268
77.3479 77.0300 76.7127
64.5309
55.5115
50.4234
3t (70Br)
ppm

# 3u (122Br)

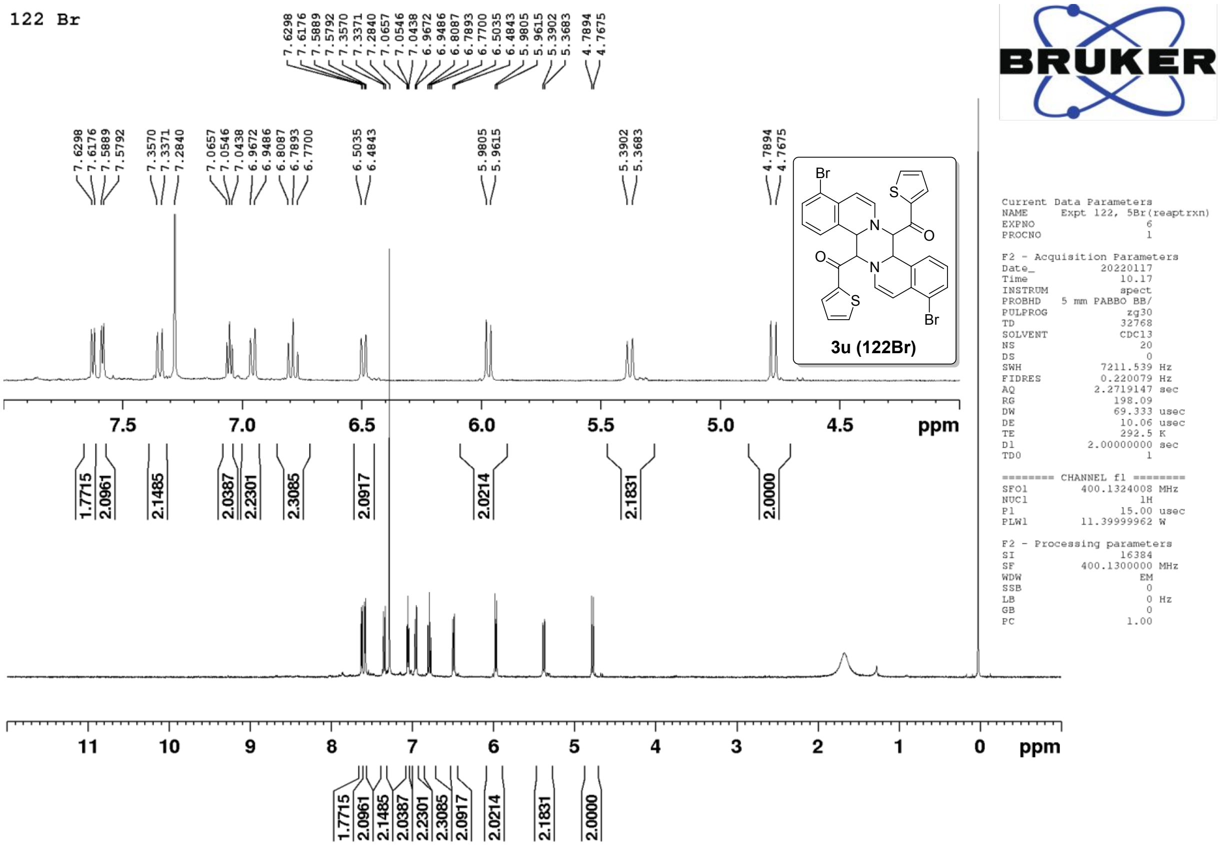
122 Br
7.6298
7.6176
7.5889
7.5792
7.3570
7.3371
7.2840
7.0657
7.0546
7.0438
6.9672
6.9486
6.8087
6.7893
6.7700
6.5035
6.4843
5.9805
5.9615
5.3902
5.3683
4.7894
4.7675
1.7715
2.0961
2.1485
2.0387
2.2301
2.3085
2.0917
2.0214
2.1831
2.0000
ppm
Br
S
N
O
O
N
S
Br
3u (122Br)
Current Data Parameters
NAME Expt 122, 5Br(reaptrxn)
EXPNO 6
PROCNO 1
F2 - Acquisition Parameters
Date_ 20220117
Time 10.17
INSTRUM spect
PROBHD 5 mm PABBO BB/
PULPROG zg30
TD 32768
SOLVENT CDCl3
NS 20
DS 0
SWH 7211.539 Hz
FIDRES 0.220079 Hz
AQ 2.2719147 sec
RG 198.09
DW 69.333 usec
DE 10.06 usec
TE 292.5 K
D1 2.00000000 sec
TD0 1
======== CHANNEL f1 ========
SFO1 400.1324008 MHz
NUC1 1H
P1 15.00 usec
PLW1 11.39999962 W
F2 - Processing parameters
SI 16384
SF 400.1300000 MHz
WDW EM
SSB 0
LB 0 Hz
GB 0
PC 1.00

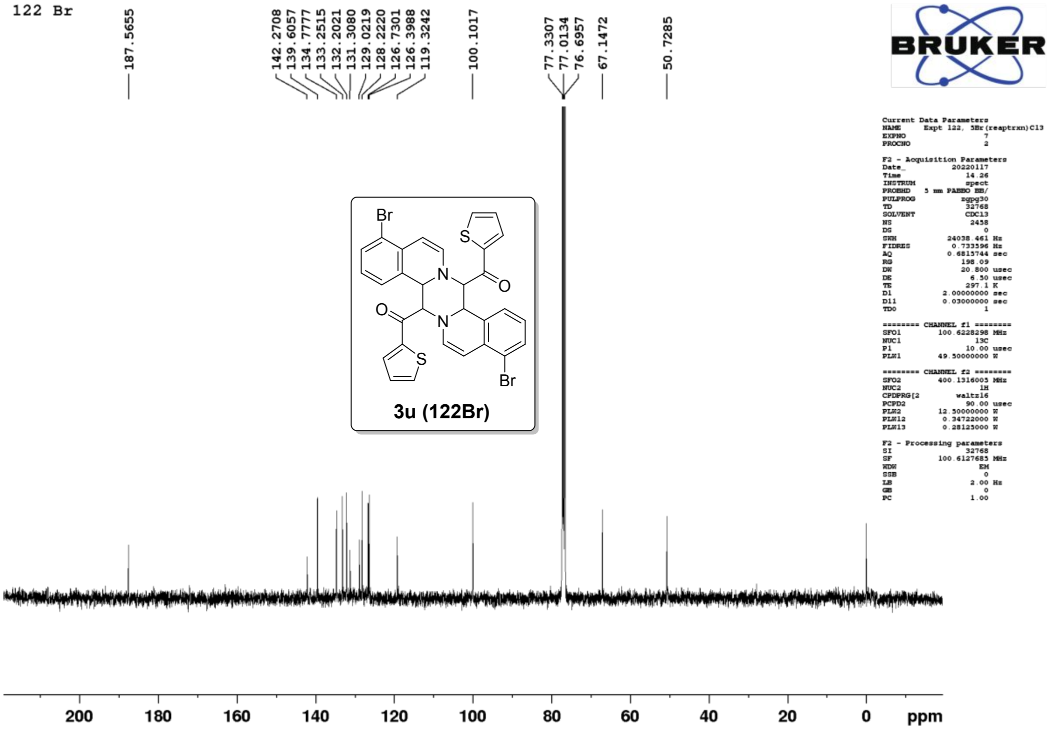
122 Br
187.5655
142.2708
139.6057
134.7777
133.2515
132.2021
131.3080
129.0219
128.2220
126.7301
126.3988
119.3242
100.1017
77.3307
77.0134
76.6957
67.1472
50.7285
Br
S
N
O
O
N
S
Br
3u (122Br)
Current Data Parameters
NAME Expt 122, 5Br(reaptrxn)C13
EXPNO 7
PROCNO 2
F2 - Acquisition Parameters
Date_ 20220117
Time 14.26
INSTRUM spect
PROBHD 5 mm PABBO BB/
PULPROG zgpg30
TD 32768
SOLVENT CDCl3
NS 2458
DS 0
SWH 24038.461 Hz
FIDRES 0.733596 Hz
AQ 0.6815744 sec
RG 198.09
DW 20.800 usec
DE 6.50 usec
TE 297.1 K
D1 2.00000000 sec
D11 0.03000000 sec
TD0 1
======== CHANNEL f1 ========
SFO1 100.6228298 MHz
NUC1 13C
P1 10.00 usec
PLW1 49.50000000 W
======== CHANNEL f2 ========
SFO2 400.1316005 MHz
NUC2 1H
CPDPRG[2 waltz16
PCPD2 90.00 usec
PLW2 12.50000000 W
PLW12 0.34722000 W
PLW13 0.28125000 W
F2 - Processing parameters
SI 32768
SF 100.6127685 MHz
WDW EM
SSB 0
LB 2.00 Hz
GB 0
PC 1.00
ppm

# 3v (115Br)

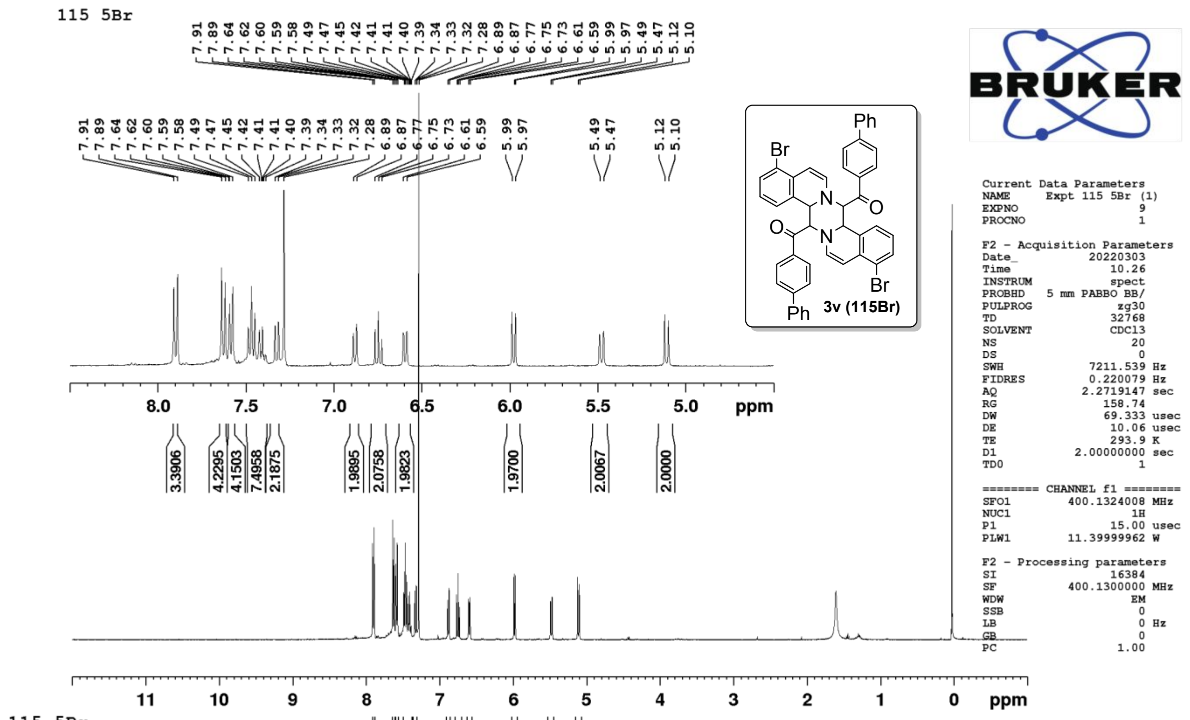
115 5Br
7.91 7.89 7.64 7.62 7.60 7.59 7.58 7.49 7.47 7.45 7.42 7.41 7.41 7.40 7.39 7.34 7.33 7.32 7.28 6.89 6.87 6.77 6.75 6.73 6.61 6.59 5.99 5.97 5.49 5.47 5.12 5.10
3.3906 4.2295 4.1503 7.4958 2.1875 1.9895 2.0758 1.9823 1.9700 2.0067 2.0000
Br
Ph
N
O
3v (115Br)
BRUKER
Current Data Parameters
NAME Expt 115 5Br (1)
EXPNO 9
PROCNO 1
F2 - Acquisition Parameters
Date_ 20220303
Time 10.26
INSTRUM spect
PROBHD 5 mm PABBO BB/
PULPROG zg30
TD 32768
SOLVENT CDCl3
NS 20
DS 0
SWH 7211.539 Hz
FIDRES 0.220079 Hz
AQ 2.2719147 sec
RG 158.74
DW 69.333 usec
DE 10.06 usec
TE 293.9 K
D1 2.00000000 sec
TD0 1
======== CHANNEL f1 ========
SFO1 400.1324008 MHz
NUC1 1H
P1 15.00 usec
PLW1 11.39999962 W
F2 - Processing parameters
SI 16384
SF 400.1300000 MHz
WDW EM
SSB 0
LB 0 Hz
GB 0
PC 1.00
ppm

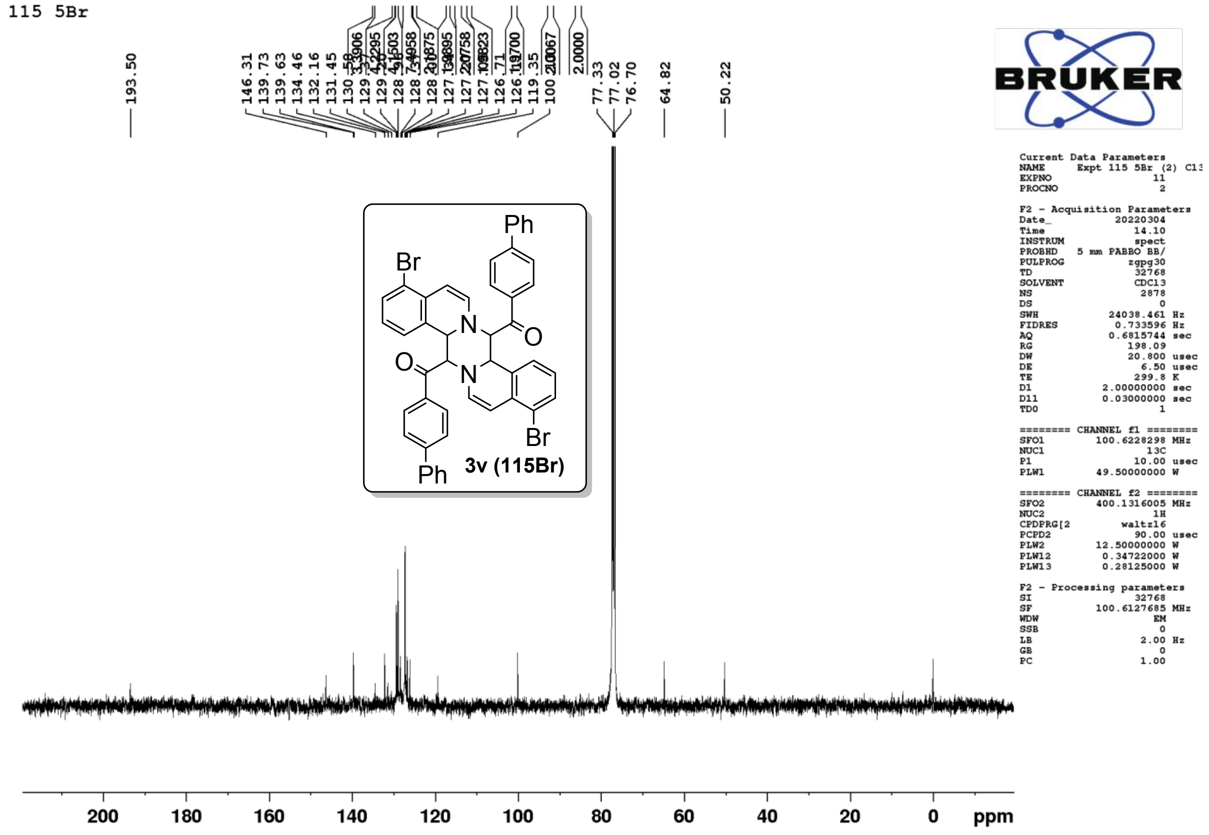
115 5Br
193.50 146.31 139.73 139.63 134.46 132.16 131.45 119.35 77.33 77.02 76.70 64.82 50.22
Br
Ph
N
O
3v (115Br)
BRUKER
Current Data Parameters
NAME Expt 115 5Br (2) C13
EXPNO 11
PROCNO 2
F2 - Acquisition Parameters
Date_ 20220304
Time 14.10
INSTRUM spect
PROBHD 5 mm PABBO BB/
PULPROG zgpg30
TD 32768
SOLVENT CDCl3
NS 2878
DS 0
SWH 24038.461 Hz
FIDRES 0.733596 Hz
AQ 0.6815744 sec
RG 198.09
DW 20.800 usec
DE 6.50 usec
TE 299.8 K
D1 2.00000000 sec
D11 0.03000000 sec
TD0 1
======== CHANNEL f1 ========
SFO1 100.6228298 MHz
NUC1 13C
P1 10.00 usec
PLW1 49.50000000 W
======== CHANNEL f2 ========
SFO2 400.1316005 MHz
NUC2 1H
CPDPRG[2 waltz16
PCPD2 90.00 usec
PLW2 12.50000000 W
PLW12 0.34722000 W
PLW13 0.28125000 W
F2 - Processing parameters
SI 32768
SF 100.6127685 MHz
WDW EM
SSB 0
LB 2.00 Hz
GB 0
PC 1.00
ppm

**Table S1:** The ADMET parameters of compound **50**

| Properties | Predicted value |
|---|---|
| **Absorption** | |
| Water solubility (log mol/L) | -3.176 |
| Caco2 permeability (log Papp in $10^{-6}$ cm/s) | 1.051 |
| Intestinal absorption (human) (% Absorbed) | 95.817 |
| Skin Permeability (log Kp) | -2.735 |
| P-glycoprotein substrate | Yes |
| P-glycoprotein I inhibitor | Yes |
| P-glycoprotein II inhibitor | Yes |
| **Distribution** | |
| VDss (human) (log L/kg) | 0.475 |
| Fraction unbound (human) (Fu) | 0.198 |
| BBB permeability (log BB) | 0.099 |
| CNS permeability (log PS) | 1.074 |
| **Metabolism** | |
| CYP2D6 substrate | Yes |
| CYP3A4 substrate | Yes |
| CYP1A2 inhibitor | No |
| CYP2C19 inhibitor | No |
| CYP2C9 inhibitor | No |
| CYP2D6 inhibitor | No |
| CYP3A4 inhibitor | No |
| **Excretion** | |
| Total Clearance (log ml/min/kg) | 0.305 |
| Renal OCT2 substrate | No |
| **Toxicity** | |
| AMES toxicity | No |
| Max. tolerated dose (human) (log mg/kg/day) | 0.787 |
| hERG I inhibitor | No |
| hERG II inhibitor | Yes |
| Oral Rat Acute Toxicity ($LD_{50}$) (mol/kg) | 2.474 |
| Oral Rat Chronic Toxicity (LOAEL) (log mg/kg_bw/day) | 1.81 |
| Hepatotoxicity | Yes |
| Skin Sensitization | No |
| *T.Pyriformis* toxicity (log ug/L) | 0.285 |
| Minnow toxicity (log mM) | -1.532 |

**Table S2.** Crystal data and structure refinement for **98**

| | | |
|---|---|---|
| Identification code | D23735 | |
| Empirical formula | $C_{36}H_{26}N_2O_6$ | |
| Formula weight | 582.59 | |
| Temperature | 200(2) K | |
| Wavelength | 0.71073 Å | |
| Crystal system | Monoclinic | |
| Space group | C 2/c | |
| Unit cell dimensions | a = 24.4502(16) Å | α= 90° |
| | b = 5.8771(4) Å | β= 96.850(2)° |
| | c = 18.9143(14) Å | γ = 90°. |
| Volume | 2698.5(3) Å3 | |
| Z | 4 | |
| Density (calculated) | 1.434 Mg/m3 | |
| Absorption coefficient | 0.098 mm-1 | |
| F(000) | 1216 | |
| Crystal size | 0.10 x 0.08 x 0.02 mm3 | |
| Theta range for data collection | 2.17 to 25.04° | |
| Index ranges | -28<=h<=26, -6<=k<=6, -22<=l<=22 | |
| Reflections collected | 14413 | |
| Independent reflections | 2372 [R(int) = 0.0601] | |
| Completeness to theta = 25.04° | 99.7 % | |
| Absorption correction | multi-scan | |
| Max. and min. transmission | 0.9980 and 0.9902 | |
| Refinement method | Full-matrix least-squares on F2 | |
| Data/restraints/parameters | 2372/0/199 | |
| Goodness-of-fit on F2 | 1.077 | |
| Final R indices [I>2sigma(I)] | R1 = 0.0419, wR2 = 0.0936 | |
| R indices (all data) | R1 = 0.0626, wR2 = 0.1044 | |
| Largest diff. peak and hole | 0.164 and -0.232 e.Å-3 | |

**Table S3.** Crystal data and structure refinement for **103Br**

| | | |
|---|---|---|
| Identification code | D23846 | |
| Empirical formula | $C_{36}H_{28}Br_2N_2O_2$ | |
| Formula weight | 680.42 | |
| Temperature | 200(2) K | |
| Wavelength | 0.71073 Å | |
| Crystal system | Monoclinic | |
| Space group | C 2/c | |
| Unit cell dimensions | a = 20.461(7) Å | α= 90° |
| | b = 7.501(3) Å | β= 90.528(11)° |
| | c = 19.144(7) Å | γ = 90°. |
| Volume | 2938.0(8) Å3 | |
| Z | 4 | |
| Density (calculated) | 1.538 Mg/m3 | |
| Absorption coefficient | 2.796 mm-1 | |
| F(000) | 1376 | |
| Crystal size | 0.07 x 0.03 x 0.02 mm3 | |
| Theta range for data collection | 2.89 to 25.31° | |
| Index ranges | -24<=h<=20, -8<=k<=9, -22<=l<=22 | |
| Reflections collected | 10441 | |
| Independent reflections | 2636 [R(int) = 0.0810] | |
| Completeness to theta = 25.04° | 98.5 % | |
| Absorption correction | multi-scan | |
| Max. and min. transmission | 0.9462 and 0.8283 | |
| Refinement method | Full-matrix least-squares on F2 | |
| Data/restraints/parameters | 2636/0/191 | |
| Goodness-of-fit on F2 | 0.958 | |
| Final R indices [I>2sigma(I)] | R1 = 0.0479, wR2 = 0.1080 | |
| R indices (all data) | R1 = 0.1030, wR2 = 0.1408 | |
| Largest diff. peak and hole | 0.268 and -0.444 e.Å-3 | |

**Table S4.** Crystal data and structure refinement for **68Br**

| | | |
|---|---|---|
| Identification code | D23821 | |
| Empirical formula | $C_{36}H_{28}Br_2$ | |
| Formula weight | 689.29 | |
| Temperature | 200(2) K | |
| Wavelength | 0.71073 Å | |
| Crystal system | Monoclinic | |
| Space group | P 21/n | |
| Unit cell dimensions | a = 13.556(2) Å | α= 90° |
| | b = 13.243(2) Å | β= 94.349(5)° |
| | c = 18.995(3) Å | γ = 90°. |
| Volume | 3400.1(9) Å3 | |
| Z | 4 | |
| Density (calculated) | 1.347 Mg/m3 | |
| Absorption coefficient | 2.436 mm-1 | |
| F(000) | 1393 | |
| Crystal size | 0.12 x 0.09 x 0.04 mm3 | |
| Theta range for data collection | 2.15 to 25.05° | |
| Index ranges | -16<=h<=15, -15<=k<=15, -22<=l<=21 | |
| Reflections collected | 41666 | |
| Independent reflections | 6013 [R(int) = 0.1016] | |
| Completeness to theta = 25.04° | 99.6 % | |
| Absorption correction | multi-scan | |
| Max. and min. transmission | 0.9089 and 0.7587 | |
| Refinement method | Full-matrix least-squares on F2 | |
| Data/restraints/parameters | 6013/2/418 | |
| Goodness-of-fit on F2 | 1.003 | |
| Final R indices [I>2sigma(I)] | R1 = 0.0609, wR2 = 0.1378 | |
| R indices (all data) | R1 = 0.1074, wR2 = 0.1613 | |
| Largest diff. peak and hole | 0.654 and -0.872 e.Å-3 | |

**Table S5.** Crystal data and structure refinement for **122Br**

| | | |
|---|---|---|
| Identification code | D23823a | |
| Empirical formula | $C_{30}H_{20}Br_2N_2O_2$ | |
| Formula weight | 664.42 | |
| Temperature | 200(2) K | |
| Wavelength | 0.71073 Å | |
| Crystal system | Monoclinic | |
| Space group | C 2/c | |
| Unit cell dimensions | a = 21.261(13) Å | α= 90° |
| | b = 6.095(4) Å | β= 98.49(2)° |
| | c = 20.426(15) Å | γ = 90°. |
| Volume | 2618(3) Å3 | |
| Z | 4 | |
| Density (calculated) | 1.686 Mg/m3 | |
| Absorption coefficient | 3.289 mm-1 | |
| F(000) | 1328 | |
| Crystal size | 0.22 x 0.10 x 0.01 mm3 | |
| Theta range for data collection | 2.58 to 25.22° | |
| Index ranges | -25<=h<=25, -7<=k<=7, -24<=l<=21 | |
| Reflections collected | 10039 | |
| Independent reflections | 2355 [R(int) = 0.0584] | |
| Completeness to theta = 25.04° | 99.1 % | |
| Absorption correction | multi-scan | |
| Max. and min. transmission | 0.9679 and 0.5315 | |
| Refinement method | Full-matrix least-squares on F2 | |
| Data/restraints/parameters | 2355/0/172 | |
| Goodness-of-fit on F2 | 0.981 | |
| Final R indices [I>2sigma(I)] | R1 = 0.0527, wR2 = 0.1405 | |
| R indices (all data) | R1 = 0.0766, wR2 = 0.1577 | |
| Largest diff. peak and hole | 1.242 and -0.783 e.Å-3 | |

**Table S6.** Crystal data and structure refinement for **115Br**

| | | |
|---|---|---|
| Identification code | D23878 | |
| Empirical formula | $C_{46}H_{30}Br_2N_2O_2$ | |
| Formula weight | 802.54 | |
| Temperature | 200(2) K | |
| Wavelength | 0.71073 Å | |
| Crystal system | Monoclinic | |
| Space group | C 2/c | |
| Unit cell dimensions | a = 28.32(3) Å | α= 90° |
| | b = 6.257(5) Å | β= 111.51(6)° |
| | c = 25.26(2) Å | γ = 90°. |
| Volume | 4164(7) Å3 | |
| Z | 4 | |
| Density (calculated) | 1.280 Mg/m3 | |
| Absorption coefficient | 1.984 mm-1 | |
| F(000) | 1624 | |
| Crystal size | 0.26 x 0.04 x 0.01 mm3 | |
| Theta range for data collection | 2.71 to 25.08° | |
| Index ranges | -33<=h<=33, -7<=k<=7, -30<=l<=30 | |
| Reflections collected | 24724 | |
| Independent reflections | 3669 [R(int) = 0.1449] | |
| Completeness to theta = 25.04° | 98.9 % | |
| Absorption correction | multi-scan | |
| Max. and min. transmission | 0.9804 and 0.6265 | |
| Refinement method | Full-matrix least-squares on F2 | |
| Data/restraints/parameters | 3669/0/245 | |
| Goodness-of-fit on F2 | 1.060 | |
| Final R indices [I>2sigma(I)] | R1 = 0.1016, wR2 = 0.2146 | |
| R indices (all data) | R1 = 0.1962, wR2 = 0.2578 | |
| Largest diff. peak and hole | 0.438 and -0.435 e.Å-3 | |
